# Supplementary material for: Factors influencing physical distancing compliance among young adults during COVID-19 pandemic in Indonesia: A photovoice mixed methods study
Source: PLOS Glob Public Health. 2022 Jan 13;2(1):e0000035. doi: 10.1371/journal.pgph.0000035 (PMC10021510; doi:10.1371/journal.pgph.0000035)
Supplement: S2 Checklist — (DOCX) [file pgph.0000035.s002.docx]

S2 Checklist. Consolidated criteria for reporting qualitative studies (COREQ): 32-item checklist

| Topic and Item No. | Guide Questions/Description | Reported on Page No. |
| --- | --- | --- |
| Domain 1: research team and reflexivity | |  |
| Personal Characteristics |  |  |
| 1. Interviewer/facilitator | Which author/s conducted the interview or focus  group? | Ahmad Junaedi (Facilitator)  Fauzan Rachmatullah (Facilitator)  Ken Ing Cherng Ong (Observer) |
| 2. Credentials | What were the researcher’s credentials? E.g. PhD, MD | Ahmad Junaedi, MHS  Ken Ing Cherng Ong, PhD  Fauzan Rachmatullah,BSc (Public Health)  Akira Shibanuma, PhD  Junko Kiriya, PhD  Masamine Jimba, PhD |
| 3. Occupation | What was their occupation at the time of the  study? | AJ-Master student, KICO-Assistant Professor, FR-Master student, AS-Assistant Professor, JK-Assistant Professor, MJ-Professor |
| 4. Gender | Was the researcher male or female? | AJ-Male, KICO-Male, FR-Male, AS-Male, JK-Female, MJ-Male |
| 5. Experience and training | What experience or training did the researcher have? | AJ-experienced FGD moderator, KICO-qualitative researcher, FR-experienced FGD moderator |
| Relationship with participants | |  |
| 6. Relationship established | Was a relationship established prior to study commencement? | Page 8 |
| 7. Participant knowledge of the interviewer | What did the participants know about the researcher? e.g. personal goals, reasons for doing the  research | Participants were provided with an information sheet and consent form which outlined the aim of the study |
| 8. Interviewer characteristics | What characteristics were reported about the interviewer/facilitator? e.g. Bias, assumptions,  reasons and interests in the research topic | Participants knew the researcher was a master student |
| Domain 2: study design | |  |
| Theoretical framework |  |  |
| 9. Methodological orientation and Theory | What methodological orientation was stated to underpin the study? e.g. grounded theory, discourse analysis, ethnography, phenomenology,  content analysis | Page 8 |
| Participant selection | |  |
| 10. Sampling | How were participants selected? e.g. purposive,  convenience, consecutive, snowball | Page 5 and Page 7 |
| 11. Method of approach | How were participants approached? e.g. face-to-face, telephone, mail, email | Page 7 - 8 |
| 12. Sample size | How many participants were in the study? | Page 13 |
| 13. Non-participation | How many people refused to participate or  dropped out? Reasons? | 1 people, no response |

| Setting | |  |
| --- | --- | --- |
| 14. Setting of data collection | Where was the data collected? e.g. home, clinic,  workplace | Page 8 (online from home) |
| 15. Presence of non-participants | Was anyone else present besides the participants  and researchers? | No |
| 16. Description of sample | What are the important characteristics of the sample? e.g. demographic data, date | Page 13 – 14 |
| Data collection | |  |
| 17. Interview guide | Were questions, prompts, guides provided by the authors? Was it pilot tested? | Page 7 - 8 |
| 18. Repeat interviews | Were repeat interviews carried out? If yes, how many? | No |
| 19. Audio/visual recording | Did the research use audio or visual recording to collect the data? | Page 8 |
| 20. Field notes | Were field notes made during and/or after the interview or focus group? | Yes, Page 8 |
| 21. Duration | What was the duration of the interviews or focus group? | Page 8 |
| 22. Data saturation | Was data saturation discussed? | Yes |
| 23. Transcripts returned | Were transcripts returned to participants for  comment and/or correction? | No |
| Domain 3: analysis and findings | |  |
| Data analysis | |  |
| 24. Number of data coders | How many data coders coded the data? | 3 People (AJ, KICO, and FR). |
| 25. Description of the coding tree | Did authors provide a description of the coding tree? | Yes |
| 26. Derivation of themes | Were themes identified in advance or derived from the data? | Themes were derived from the data |
| 27. Software | What software, if applicable, was used to manage the data? | Microsoft Excel |
| 28. Participant checking | Did participants provide feedback on the findings? | No |
| Reporting | |  |
| 29. Quotations presented | Were participant quotations presented to illustrate the themes / findings? Was each  quotation identified? e.g. participant number | Page 14 - 17, Figure 2-4 |
| 30. Data and findings consistent | Was there consistency between the data  presented and the findings? | Yes, Page 14 – 22 |
| 31. Clarity of major themes | Were major themes clearly presented in the findings? | Yes, Page 18 – 22 |
| 32. Clarity of minor themes | Is there a description of diverse cases or discussion of minor themes? | Page 18 – 22 |
