# Supplementary material for: Factors influencing physical distancing compliance among young adults during COVID-19 pandemic in Indonesia: A photovoice mixed methods study
Source: PLOS Glob Public Health. 2022 Jan 13;2(1):e0000035. doi: 10.1371/journal.pgph.0000035 (PMC10021510; doi:10.1371/journal.pgph.0000035)
Supplement: S1 Table — (DOCX) [file pgph.0000035.s003.docx]

**S1 Table. Descriptive statistics of COVID-19-related variables and religious and tradition activities of 330 young adults**

| **COVID-19 variables** | **n** | **%** | |  |  |
| --- | --- | --- | --- | --- | --- |
| **COVID-19 testing** |  |  | |  |  |
| Get tested | 115 | 34.8 | |  |  |
| Not tested yet | 215 | 65.2 | |  |  |
| **Perceived risk** | | |  | |  |
| Unlikely | 10 | 3.0 | |  |  |
| Neither likely nor unlikely | 131 | 39.7 | |  |  |
| Likely | 189 | 57.3 | |  |  |
| **Perceived severity** | | |  | |  |
| Not severe | 117 | 35.5 | |  |  |
| Neither severe nor not severe | 175 | 53.0 | |  |  |
| Severe | 38 | 11.5 | |  |  |
| **Perceived susceptibility** | | |  | |  |
| Unlikely | 59 | 17.9 | |  |  |
| Neither likely nor unlikely | 141 | 42.7 | |  |  |
| Likely | 130 | 39.4 | |  |  |
| **Duration of restriction compliance** | | |  |  |  |
| Less than 1 week and never | 30 | 9.1 | |  |  |
| 1 week to 1 month | 107 | 32.4 | |  | |
| More than 1 month | 193 | 58.5 | |  | |
| **Receiving workplace/school support** | | |  | |  |
| Less than 3 type supports | 129 | 39.1 | |  |  |
| 3 type supports or more | 201 | 60.9 | |  |  |
| **Receiving government support** | | |  | |  |
| Not received | 109 | 33.0 | |  |  |
| Received | 221 | 67.0 | |  |  |
| **Reason to comply: Family and neighborhood** | | |  | |  |
| Not agree | 150 | 45.5 | |  |  |
| Agree | 180 | 54.5 | |  |  |
| **Reason to comply: Urge from some authorities and public figure** | | |  | |  |
| Not agree | 183 | 55.5 | |  |  |
| Agree | 147 | 44.5 | |  |  |
| **Reason to comply: Responsibility and fear** | | |  | |  |
| Not agree | 85 | 25.8 | |  |  |
| Agree | 245 | 74.2 | |  |  |
| **Knowledge of COVID-19 score**, mean [SD] | 12.5 [1.8] |  | |  |  |
| **Knowledge of COVID-19 prevention score**, mean [SD] | 12.0 [1.1] |  | |  |  |
| **Understanding physical distancing guideline score**, mean [SD] | 7.5 [0.9] |  | |  | |
| **Feasibility to comply with physical distancing score**, mean [SD] | 15.9 [2.8] |  | |  | |
| **Perceived benefit score**, mean [SD] | 17.1 [3.3] |  | |  | |
| **Religious and tradition activities** | **n** | **%** | |  | |
| **Special praying together outside during pandemic** | | |  | |  |
| No, at home with family | 230 | 69.7 | |  |  |
| Yes | 81 | 24.5 | |  |  |
| Never performed special praying | 19 | 5.8 | |  |  |
| **Break Ramadan fasting together outside during pandemic** | | |  | |  |
| No, at home with family | 264 | 80.0 | |  |  |
| Yes | 13 | 3.9 | |  |  |
| Never performed fasting and not joined break fasting with others | 53 | 16.1 | |  |  |
| **Religious celebration during pandemic** | | |  | |  |
| No, at home with family only | 200 | 60.6 | |  |  |
| Yes | 92 | 27.9 | |  |  |
| Never celebrate and not joined celebration with others | 38 | 11.5 | |  |  |
| ***Mudik* tradition during pandemic** | | |  | |  |
| Not joined *mudik* this year | 236 | 71.5 | |  |  |
| Yes | 15 | 4.6 | |  |  |
| Having hometown in the Jakarta Metropolitan Area | 79 | 23.9 | |  |  |
