# Supplementary material for: Factors influencing physical distancing compliance among young adults during COVID-19 pandemic in Indonesia: A photovoice mixed methods study
Source: PLOS Glob Public Health. 2022 Jan 13;2(1):e0000035. doi: 10.1371/journal.pgph.0000035 (PMC10021510; doi:10.1371/journal.pgph.0000035)
Supplement: S2 Table — (DOCX) [file pgph.0000035.s004.docx]

**S2 Table. Hierarchical linear regression analysis with physical distancing compliance score (full version)**

|  | | | | | |  | | | | | |
| --- | --- | --- | --- | --- | --- | --- | --- | --- | --- | --- | --- |
| Variables | Model 1  *β* | | Model 2  *β* | | Model 3  *β* | | | | | |  |
| **STEP 1**  **Covariates: Sociodemographic characteristics** |  | |  | |  | | | | | |  |
| **Age** | 0.07 | | 0.05 | | 0.07 | | | | | |  |
| **Place of working/studying** (vs. Jakarta Pusat) | | | | | | | | | |  |  |
| Jakarta Utara | -0.09 | -0.08 | | -0.07 | | | | |  |  |  |
| Jakarta Selatan | 0.01 | -0.03 | | -0.06 | | | | |  |  |  |
| Jakarta Barat | -0.11 | -0.09 | | -0.09 | | | | |  |  |  |
| Jakarta Timur | 0.02 | 0.03 | | 0.06 | | | | |  |  |  |
| **Place of living** (vs. Jakarta Pusat) | | | | | | |  |  |  |  |  |
| Jakarta Utara | 0.04 | 0.05 | | 0.04 | | | | |  |  |  |
| Jakarta Selatan | 0.03 | 0.09 | | 0.09 | | | | |  |  |  |
| Jakarta Barat | 0.12 | 0.14 | | 0.11 | | | | |  |  |  |
| Jakarta Timur | -0.02 | 0.01 | | -0.01 | | | | |  |  |  |
| Kabupaten Bogor | -0.05 | -0.08 | | -0.06 | | | | |  |  |  |
| Kota Bogor | -0.07 | 0.02 | | 0.02 | | | | |  |  |  |
| Kota Depok | -0.03 | 0.05 | | 0.03 | | | | |  |  |  |
| Kota Tangerang | -0.04 | -0.01 | | 0.03 | | | | |  |  |  |
| Kota Tangerang Selatan | 0.00 | 0.05 | | 0.05 | | | | |  |  |  |
| Kabupaten Bekasi | 0.00 | 0.03 | | 0.01 | | | | |  |  |  |
| Kota Bekasi | 0.00 | 0.06 | | 0.04 | | | | |  |  |  |
| **Sex** (vs. Men) | 0.11 | 0.05 | | -0.01 | | | | |  |  |  |
| **Education level** (vs. High school) | | | | | | |  |  |  |  |  |
| Diploma | -0.02 | -0.02 | | -0.05 | | | | |  |  |  |
| University | -0.10 | -0.01 | | -0.05 | | | | |  |  |  |
| Graduate and Postgraduate | -0.09 | -0.06 | | -0.11 | | | | |  |  |  |
| **Occupation** (vs Public Sector) | | | | | | |  |  |  |  |  |
| Private sector | -0.13 | -0.11 | | -0.10 | | | | |  |  |  |
| Health sector | **0.11*** | 0.05 | | 0.05 | | | | |  |  |  |
| College student | -0.14 | -0.07 | | -0.06 | | | | |  |  |  |
| Others | -0.04 | -0.02 | | 0.02 | | | | |  |  |  |
| **Resuming work at office/study at school (as of July 21, 2020)** (vs. Yes) | | | | | | |  |  |  |  |  |
| Working/studying from home | **0.20**** | **0.14*** | | **0.14*** | | | | |  |  |  |
| Never work/study from home | **-0.12*** | -0.06 | | -0.08 | | | | |  |  |  |
| **Living situation** (vs. Alone) |  |  | |  | | | | |  |  |  |
| With one person or more | -0.05 | -0.01 | | -0.00 | | | | |  |  |  |
| **Monthly income/allowance before pandemic in Indonesia rupiah (in US dollar)** (vs. Less than 3 million/212) | | | | | | |  |  |  |  |  |
| 3 to 6 million (212 to 424) | -0.00 | 0.05 | | 0.12 | | | | |  |  |  |
| 6 to 9 million (424 to 636) | 0.05 | 0.03 | | 0.08 | | | | |  |  |  |
| More than 9 million (636) | 0.04 | 0.00 | | 0.05 | | | | |  |  |  |
| **STEP 2**  **Predictor variables: COVID-19 related variables** | | | | | | |  |  |  |  |  |
| **COVID testing** (vs. Get tested) | | | | | | |  |  |  |  |  |
| Not tested yet |  | 0.02 | | 0.00 | | | | |  |  |  |
| **Knowledge of COVID score** |  | -0.02 | | -0.04 | | | | |  |  |  |
| **Knowledge of COVID prevention score** |  | -0.01 | | -0.01 | | | | |  |  |  |
| **Perceived severity** (vs. Not severe) | | | | | | |  |  |  |  |  |
| Neither severe nor not severe |  | 0.03 | | 0.02 | | | | |  |  |  |
| Severe |  | 0.04 | | 0.02 | | | | |  |  |  |
| **Perceived susceptibility** (vs. Unlikely) | | | | | | |  |  |  |  |  |
| Neither likely nor unlikely |  | -0.04 | | -0.05 | | | | |  |  |  |
| Likely |  | -0.06 | | -0.04 | | | | |  |  |  |
| **Reason compliance: Family and neighborhood** (vs. Not agree) | | | | | | | |  |  |  |  |
| Agree |  | 0.04 | | 0.06 | | | | |  |  |  |
| **Reason compliance: Urge from some authorities and public figure** (vs. Not agree) | | | | | | | |  |  |  |  |
| Agree |  | 0.05 | | 0.03 | | | | |  |  |  |
| **Reason compliance: responsibility and fear** (vs. Not agree) | | | | | | | |  |  |  |  |
| Agree |  | 0.03 | | 0.04 | | | | |  |  |  |
| **Feasibility to comply with physical distancing score** |  | **0.23***** | | **0.24***** | | | | |  |  |  |
| **Duration of restriction compliance** (vs. Never and less than 1 week) | | | | | | |  |  |  |  |  |
| 1 week to 1 month |  | **0.26**** | | **0.24**** | | | | |  |  |  |
| More than 1 month |  | **0.39***** | | **0.35***** | | | | |  |  |  |
| **Understanding physical distancing guideline score** |  | **0.27***** | | **0.21***** | | | | |  |  |  |
| **Perceived benefit score** |  | 0.02 | | 0.02 | | | | |  |  |  |
| **Receiving workplace/School support** (vs. Less than 3 type of supports) | | | | | | |  |  |  |  |  |
| 3 type supports or more |  | -0.01 | | -0.00 | | | | |  |  |  |
| **Receiving government support** (vs. Not received) | | | | | | |  |  |  |  |  |
| Received |  | -0.00 | | 0.01 | | | | |  |  |  |
| **STEP 3**  **Predictor variables: Religious and tradition-related variables** | | | | | | |  |  |  |  |  |
| **Special praying together outside during pandemic** (vs. Not, at home with family) | | | | | | |  |  |  |  |  |
| Yes |  |  | | -0.09 | | | | |  |  |  |
| Never performed special praying |  |  | | -0.04 | | | | |  |  |  |
| **Break Ramadan fasting together outside during pandemic** (vs. No, at home with family) | | | | | | |  |  |  |  |  |
| Yes |  |  | | -0.11 | | | | |  |  |  |
| Never performed fasting and not joined break fasting with others |  |  | | -0.04 | | | | |  |  |  |
| **Religious celebration during pandemic** (vs. No, at home with family only) | | | | | | |  |  |  |  |  |
| Yes |  |  | | **-0.15*** | | | | |  |  |  |
| Never celebrate and not joined celebration with others |  |  | | 0.13 | | | | |  |  |  |
| ***Mudik* tradition during pandemic** (vs. Not joined *mudik* this year) | | | | | | |  |  |  |  |  |
| Yes |  |  | | 0.04 | | | | |  |  |  |
| Having hometown in the Jakarta Metropolitan Area |  |  | | -**0.12*** | | | | |  |  |  |
| R^2^ | 0.15 | 0.40 | | 0.45 | | | | |  |  |  |
| ΔR^2^ | 0.15 | **0.25***** | | **0.05***** | | | | |  |  |  |

R^2^–variance; ΔR^2^–change in variance. Statistical significance indicated by * *p* <0.05; ** *p* <0.01; *** *p* <0.001.

Model 1 was adjusted for socio-demographic characteristics

Model 2 was adjusted for socio-demographic characteristics and COVID-19-related variables

Model 3 was adjusted for socio-demographic characteristics, COVID-19-related variables, and religious- and tradition- related variables
