# Supplementary material for: Factors influencing physical distancing compliance among young adults during COVID-19 pandemic in Indonesia: A photovoice mixed methods study
Source: PLOS Glob Public Health. 2022 Jan 13;2(1):e0000035. doi: 10.1371/journal.pgph.0000035 (PMC10021510; doi:10.1371/journal.pgph.0000035)
Supplement: S1 Questionnaire — (DOCX) [file pgph.0000035.s005.docx]

**S1 Questionnaire. Compliance questionnaire (English)**

**Compliance Questionnaire**

Hello, my name is Ahmad Junaedi. I am from the University of Tokyo. Thank you for your interest to participate in this research. I am conducting research titled “Coronavirus disease (COVID-19) pandemic: Barriers and facilitators to physical distancing among young adults in Jakarta Metropolitan area, Indonesia." Please click next button to continue.

**Part I : Screening questions**

Q1a. Your birth month :

January = 1

February = 2

March = 3

April = 4

May = 5

June = 6

July = 7

August = 8

September = 9

October = 10

November = 11

December = 12

Q1b. Your birth year :

*[If Q1 is not between 1985 - 2000, skip to END of questionnaire]*

Q2. Are you belong to employee/self-employed or student who work/study in Jakarta since January until now? : No = 0 Yes = 1

*[If Q2 = 0, skip to END of questionnaire]*

Q3. Where do you work : Jakarta Pusat = 0

Jakarta Utara = 1

Jakarta Selatan = 2

Jakarta Barat = 3

Jakarta Timur = 4

Others = 5

*[If Q3 = 5, skip to END of questionnaire]*

Q4. Where do you live :

Q4a. City Jakarta Pusat = 0

Jakarta Utara = 1

Jakarta Selatan = 2

Jakarta Barat = 3

Jakarta Timur = 4

Kabupaten Bogor = 5

Kota Bogor = 6

Kota Depok = 7

Kota Tangerang = 8

Kota Tangerang Selatan = 9

Kabupaten Bekasi = 10

Others = 11 (specify):___________

*[If Q4a = 11, skip to END of questionnaire]*

Q4b. Sub-district: ________________ (pop-up list is provided based on the answer of Q4a)

**Part II : Main Section**

Q5. Sex : Male = 0 Female = 1

Q6. What is the highest level of education that you have completed?

No formal education = 1

Primary school = 2

Lower secondary school = 3

Upper secondary school = 4

Vocational School (D1/D2/D3) = 5

University (S1) = 6

Graduate/Post-Graduate (S2/S3) = 7

Q7. What is your occupation (specify): ______________________

Q7a. Is your workplace/school ask you to work from office/study at school/allow you to work there?

Yes = 1

Not yet = 2

Never have chance to work/study from home = 3

*[If Q7a = 2 or 3, skip to Q8]*

*Q7b. When did you back to work from office/study from school?*

June = 1, date:____

July = 2, date:____

Q8. How many people who live with you in your place?

Living alone = 1 Living with one or more people = 2

Q9. Are you or have you been infected with the novel coronavirus?

Yes, confirmed (positive) = 1 Yes, but not yet confirmed (waiting result) = 2 No, the result was negative = 0 Don’t know = 3

Q10. Do you know people in your immediate social environment who are or have been infected with the novel coronavirus?

Yes, there is people with confirmed (positive) = 1 Yes, but not yet confirmed (waiting result) = 2 No, the result was negative = 0 Don’t know = 3

**Knowledge of COVID-19**

Q11. What are common signs or symptoms of an infection with the new coronavirus?

Please select ‘True or ‘False’ for each option.

1. Nose bleeds True = 1 False = 0
2. Cough True = 1 False = 0
3. Fever True = 1 False = 0
4. Skin rash True = 1 False = 0
5. Constipation True = 1 False = 0
6. Shortness of breath True = 1 False = 0
7. Frequent urination True = 1 False = 0
8. Tiredness True = 1 False = 0

Q12. Do you think people who don’t develop any symptoms and don't feel unwell can be positive of the new coronavirus?

Yes = 1 No = 0 Don’t know = 2

Q13. When people have been infected, what age groups are most likely to die from the illness caused by the new coronavirus?

Please select all options that you think are correct.

1. Baby Yes = 1 No = 0
2. Children Yes = 1 No = 0
3. Adolescent Yes = 1 No = 0
4. Young adults Yes = 1 No = 0
5. Adult Yes = 1 No = 0
6. Elderly Yes = 1 No = 0

Q14. Are those people with other health problems more likely to die from an infection with the new coronavirus disease than those without any other health problems?

Yes = 1 No = 0 Don’t know = 2

Q15. What is the main way in which people are currently getting infected with the new coronavirus?

Please select one response option only.

Eating or touching bats = 1

Fecal contaminants in drinking water = 2

Unhygienic preparation of food = 3

Sexual intercourse or sharing of needles for drug use = 4

Mosquito bites = 5

Droplets of saliva that land in the mouths or noses of people who are nearby when an infected person sneezes or coughs = 6

Eating undercooked meat products = 7

Directly coming into touch with someone's bodily fluids like blood, vomit, or sweat = 8

Snake bites or touching snakes = 9

Q16. Approximately how far do you think the new coronavirus can travel through the air to transmit the infection from one person to another?

0 to 1 meter = 1 1 to 2 meter = 2 2 to 3 meter = 3 3 to 4 meter = 4

4 to 5 meter = 5 more than 5 meter = 6

**Knowledge of COVID-19 prevention**

Q17. Which of the following actions help prevent catching an infection with the new coronavirus?

Please select 'True' or 'False' for each option

1. Wear a face mask True = 1 False = 0
2. Getting a vaccination against pneumonia True = 1 False = 0
3. Gargling mouthwash True = 1 False = 0
4. Washing your hands True = 1 False = 0
5. Eating garlic True = 1 False = 0
6. Avoid close contact with people who are sick True = 1 False = 0
7. Taking antibiotics True = 1 False = 0
8. Drink *Jamu*/*Tolak Angin* True = 1 False = 0
9. Putting sesame oil on your skin True = 1 False = 0
10. Avoiding touching your eyes, nose, and mouth with unwashed hands

True = 1 False = 0

1. Regularly rinsing your nose with saline True = 1 False = 0
2. Cleaning hand using hand sanitizer True = 1 False = 0
3. Cover nose and mouth with arm or tissue when coughing

True = 1 False = 0

**Perceived risk of infection**

Q18. What is your probability of getting infected with the novel coronavirus?

Extremely unlikely = 1

Unlikely = 2

Somewhat unlikely= 3

Neither likely nor unlikely= 4

Somewhat likely= 5

Likely= 6

Extremely likely = 7

Q19. How severe would contracting the novel coronavirus be for you?

Not very severe = 1

Not severe = 2

Somewhat not severe = 3

Neither severe nor not severe = 4

Somewhat severe= 5

Severe= 6

Very Severe = 7

Q20. How susceptible do you consider yourself to an infection with the novel coronavirus?

Not at all susceptible = 1

Not susceptible = 2

Somewhat not susceptible = 3

Neither susceptible nor not susceptible = 4

Somewhat susceptible = 5

Susceptible= 6

Very Susceptible = 7

*[PSBB Transition]*

*[the large-scale social restriction has been relaxed by the DKI Jakarta governor, and starting to enter transition phase since 5 June 2020 in DKI Jakarta area]*

**Physical Distancing Compliance**

Q21. Which of the following physical distancing measures have you taken to prevent infection from the novel coronavirus?

Please indicate for all measures below whether you have already taken them.

1. Maintaining minimum 1 meter from other people

Always = 1 Sometimes = 2 Never = 3

1. Avoiding handshaking

Always = 1 Sometimes = 2 Never = 3

1. Avoiding hugging

Always = 1 Sometimes = 2 Never = 3

1. Avoiding taking public transportation/Avoiding rush hours

Always = 1 Sometimes = 2 Never = 3

1. Working from home

Always = 1 Sometimes = 2 Never = 3

1. Avoiding mass gathering

Always = 1 Sometimes = 2 Never = 3

1. Postponing meet in person with friends or other family member who are not live in the same house

Always = 1 Sometimes = 2 Never = 3

1. When you are sick, not visiting elderly/minimize direct interaction with elderly if you live in the same house.

Always = 1 Sometimes = 2 Never = 3 Not sick = 4

1. For time being, praying at home

Always = 1 Sometimes = 2 Never = 3

Q22. Please indicate how much you disagree or agree with the following statements

*[The physical distancing means avoiding use of public transportation/use it during rush hours, avoiding physical contact such as salam/handshake, hug, maintain distance, avoiding meeting other people such as remain working/studying from home, avoiding crowd, and avoiding meeting other people who are not live in the same house.]*

I do physical distancing because:

1. My family and friends also do physical distancing.

Strongly disagree = 1

Disagree = 2

Somewhat disagree = 3

Neither agree not disagree = 4

Somewhat agree = 5

Agree = 6

Strongly agree = 7

1. People in your immediate social environment also do physical distancing.

Strongly disagree = 1

Disagree = 2

Somewhat disagree = 3

Neither agree not disagree = 4

Somewhat agree = 5

Agree = 6

Strongly agree = 7

1. Local authorities (RT/RW/Lurah/Camat) urge me to do physical distancing

Strongly disagree = 1

Disagree = 2

Somewhat disagree = 3

Neither agree not disagree = 4

Somewhat agree = 5

Agree = 6

Strongly agree = 7

1. Government (President/Mayor/Governor/Ministry of Health) urge me to do physical distancing

Strongly disagree = 1

Disagree = 2

Somewhat disagree = 3

Neither agree not disagree = 4

Somewhat agree = 5

Agree = 6

Strongly agree = 7

1. Health authorities (Puskesmas) urge me to do physical distancing

Strongly disagree = 1

Disagree = 2

Somewhat disagree = 3

Neither agree not disagree = 4

Somewhat agree = 5

Agree = 6

Strongly agree = 7

1. Public figures (Religious leader/Ethnic Leader/Actor/Singer/etc.) urge me to do physical distancing

Strongly disagree = 1

Disagree = 2

Somewhat disagree = 3

Neither agree not disagree = 4

Somewhat agree = 5

Agree = 6

Strongly agree = 7

1. I want to protect others by doing physical distancing.

Strongly disagree = 1

Disagree = 2

Somewhat disagree = 3

Neither agree not disagree = 4

Somewhat agree = 5

Agree = 6

Strongly agree = 7

1. Doing physical distancing is my civic duty and I want to be a good citizen

Strongly disagree = 1

Disagree = 2

Somewhat disagree = 3

Neither agree not disagree = 4

Somewhat agree = 5

Agree = 6

Strongly agree = 7

1. I afraid to be infected if I don’t do physical distancing

Strongly disagree = 1

Disagree = 2

Somewhat disagree = 3

Neither agree not disagree = 4

Somewhat agree = 5

Agree = 6

Strongly agree = 7

1. My employer/my school urges me to do physical distancing.

Strongly disagree = 1

Disagree = 2

Somewhat disagree = 3

Neither agree not disagree = 4

Somewhat agree = 5

Agree = 6

Strongly agree = 7

**Practicalities of Physical Distancing**

Q23. How would you rate this following physical distancing measures are easy to practice?

1. Maintaining minimum 1 meter from other people

Extremely difficult = 1

Difficult = 2

Somewhat difficult = 3

Neither easy to do nor difficult = 4

Somewhat easy to do = 5

Easy to do = 6

Very easy to do = 7

1. Avoiding handshaking

Extremely difficult = 1

Difficult = 2

Somewhat difficult = 3

Neither easy to do nor difficult = 4

Somewhat easy to do = 5

Easy to do = 6

Very easy to do = 7

1. Avoiding hugging

Extremely difficult = 1

Difficult = 2

Somewhat difficult = 3

Neither easy to do nor difficult = 4

Somewhat easy to do = 5

Easy to do = 6

Very easy to do = 7

1. Avoiding taking public transportation/Avoiding rush hours

Extremely difficult = 1

Difficult = 2

Somewhat difficult = 3

Neither easy to do nor difficult = 4

Somewhat easy to do = 5

Easy to do = 6

Very easy to do = 7

1. Working from home

Extremely difficult = 1

Difficult = 2

Somewhat difficult = 3

Neither easy to do nor difficult = 4

Somewhat easy to do = 5

Easy to do = 6

Very easy to do = 7

1. Avoiding mass gathering

Extremely difficult = 1

Difficult = 2

Somewhat difficult = 3

Neither easy to do nor difficult = 4

Somewhat easy to do = 5

Easy to do = 6

Very easy to do = 7

1. Postponing meet in person with friends or other family member who are not live in the same house

Extremely difficult = 1

Difficult = 2

Somewhat difficult = 3

Neither easy to do nor difficult = 4

Somewhat easy to do = 5

Easy to do = 6

Very easy to do = 7

1. When you are sick, not visiting elderly/minimize direct interaction with elderly if you live in the same house.

Extremely difficult = 1

Difficult = 2

Somewhat difficult = 3

Neither easy to do nor difficult = 4

Somewhat easy to do = 5

Easy to do = 6

Very easy to do = 7

1. For time being, praying at home

Extremely difficult = 1

Difficult = 2

Somewhat difficult = 3

Neither easy to do nor difficult = 4

Somewhat easy to do = 5

Easy to do = 6

Very easy to do = 7

**Length of Physical Distancing**

*[The physical distancing means avoiding use of public transportation/use it during rush hours, avoiding physical contact such as salam/handshake, hug, maintain distance, avoiding meeting other people such as remain working/studying from home, avoiding crowd, and avoiding meeting other people who are not live in the same house.]*

Q24. From PSBB transition, 5 June 2020, until now, how many days did you do physical distancing?

Never = 1 Less than 1 week = 2 1 week to 2 weeks = 3

2 weeks to 3 weeks = 4 3 weeks to 1 month = 5 more than 1 month = 6

Q25. After entering PSBB transition phase on 5 June 2020, how long ideally physical distancing should be conducted from that day?

No need to implement = 1 Less than 1 week = 2 1 week to 2 weeks = 3

2 weeks to 3 weeks = 4 3 weeks to 1 month = 5 1 month to 2 months = 6

more than 2 months = 7

**Knowledge of Physical Distancing Protocol**

Q26. Which of the following physical distancing measures are true or false?

1. It is okay to physically close as usual with others

True = 1 False = 0

1. It is okay to handshake with others

True = 1 False = 0

1. It is okay to hug with others

True = 1 False = 0

1. It is okay to take crowded public transportation

True = 1 False = 0

1. It is okay to keep go to work

True = 1 False = 0

1. It is okay to join mass gathering or public facility

True = 1 False = 0

1. It is okay to gather with friends or other family member who are not live in the same house

True = 1 False = 0

1. It is okay when you are sick to visit elderly/keep direct interaction as usual with elderly if you live in the same house

True = 1 False = 0

1. It is okay to pray together with others as usual in Mosque/Church/Pura/Temple/Pagoda/other places that usually you do for praying

True = 1 False = 0

Q27. Based on Indonesia COVID-19 guideline, which answer in each following statement is correct?

1. Asymptomatic people who just go back to Indonesia/their home after visiting a country/city with imported the new coronavirus case should:

self-monitoring for 14 days = 1

self-quarantine for 14 days = 2

get RT PCR tested and home-quarantine regardless the result = 3

can do any activities outside home as usual = 4

Don’t know = 5

1. Asymptomatic people who just go back to Indonesia/their home after visiting a country/city with local transmission the new coronavirus case should

self-monitoring for 14 days = 1

self-quarantine for 14 days = 2

get RT PCR tested and home-quarantine regardless the result = 3

can do any activities outside home as usual = 4

Don’t know = 5

1. Asymptomatic people who had contact with the new coronavirus confirmed case should:

self-monitoring for 14 days = 1

self-quarantine for 14 days = 2

get RT PCR tested and home-quarantine regardless the result = 3

can do any activities outside home as usual = 4

Don’t know = 5

Q28. If you have a fever or cough and recently visited a country or city with the new coronavirus outbreak, or spent time with someone who did, what would be the best course of action?

Please select one response option only.

Go to referral COVID-19 hospital, such as by taxi or public transport to avoid driving yourself = 1

Have someone drive you to the emergency unit in referral COVID-19 hospital = 2

Call 119 or go to referral COVID-19 hospital with your own transportation = 3

Rest more than usual and then call 119 or go to referral COVID-19 hospital if you still feel ill after 2-3 days = 4

**Perceived benefit of Physical Distancing**

Q29. Please indicate how much you disagree or agree with the following statements

After doing physical distancing:

1. I believe I will not be infected

Strongly disagree = 1

Disagree = 2

Somewhat disagree = 3

Neither agree not disagree = 4

Somewhat agree = 5

Agree = 6

Strongly agree = 7

1. I believe I can protect others

Strongly disagree = 1

Disagree = 2

Somewhat disagree = 3

Neither agree not disagree = 4

Somewhat agree = 5

Agree = 6

Strongly agree = 7

1. I believe I can reduce healthcare workers burden

Strongly disagree = 1

Disagree = 2

Somewhat disagree = 3

Neither agree not disagree = 4

Somewhat agree = 5

Agree = 6

Strongly agree = 7

1. I believe I can stop the transmission

Strongly disagree = 1

Disagree = 2

Somewhat disagree = 3

Neither agree not disagree = 4

Somewhat agree = 5

Agree = 6

Strongly agree = 7

1. I believe I contribute to help government handling this outbreak

Strongly disagree = 1

Disagree = 2

Somewhat disagree = 3

Neither agree not disagree = 4

Somewhat agree = 5

Agree = 6

Strongly agree = 7

**Physical Distancing Support from Employer/School**

Q30. Please indicate for all supports below whether your employer/school have already given to you.

1. Online Class/Online Meeting from home

Supported = 1 Not Supported = 2 Don’t know = 3

1. Food supplies

Supported = 1 Not Supported = 2 Don’t know = 3

1. Paid leave/tuition fee discount

Supported = 1 Not Supported = 2 Don’t know = 3

1. Free shuttle (taxi or employer’s car) from workplace to home/home to workplace (worker only)

Supported = 1 Not Supported = 2 Don’t know = 3 Not applicable = 4

1. Online administration for school (student only)

Supported = 1 Not Supported = 2 Don’t know = 3 Not applicable = 4

1. Protection kits (mask, face shield, gloves, and hand sanitizer)

Supported = 1 Not Supported = 2 Don’t know = 3

1. Others (specify): ____________________________

**Physical Distancing Support from Government**

Q31. How is your monthly income (worker)/allowance (student) before COVID-19? (in Indonesian Rupiah)

Less than 3 million = 1 3 to 6 million = 2 6 to 9 million = 3

more than 9 million = 4

Q32. Please indicate for all supports below whether government (President/Governor/Mayor) have already given to you.

1. Food supplies

Supported = 1 Not Supported = 2 Don’t know = 3

1. Electricity bills discount

Supported = 1 Not Supported = 2 Don’t know = 3

1. Postponing installment for motorbike/home/etc.

Supported = 1 Not Supported = 2 Don’t know = 3

1. Receiving “*Prakerja*” Card

Supported = 1 Not Supported = 2 Don’t know = 3

1. Incentive

Supported = 1 Not Supported = 2 Don’t know = 3

1. Protection kits (mask, face shield, gloves, and hand sanitizer)

Supported = 1 Not Supported = 2 Don’t know = 3

1. Disinfectant spraying at home or private vehicle

Supported = 1 Not Supported = 2 Don’t know = 3

1. Others (specify): ____________________________

**Religious routine activities**

Q33. Are you still perform praying togethers (Daily Salat/Friday Salat/Tarawih/Salat Eid/Good Friday/Weekly Misa/Praying at Pagoda/Praying at Pura/Praying at Temple) with others in usual place (Mosque/Church/Pura/Temple/Pagoda/other places that usually you do for praying)?

Always = 1 Sometimes = 2 Never, I pray at home = 3 Never performed praying = 4

*[If Q33= 1 or 4, skip to Q35]*

Q34. How would you rate for not performing praying togethers during the novel coronavirus outbreak?

Extremely difficult = 1

Difficult = 2

Somewhat difficult = 3

Neither easy to do nor difficult = 4

Somewhat easy to do = 5

Easy to do = 6

Very easy to do = 7

**Tradition Religious activities**

Q35. Did you join “buka puasa bersama”/breaking Ramadan fast together during Ramadan with your Muslim friends/in event that held by your employer/school?

Always = 1 Sometimes = 2 Never, I do it at home (family only) = 3 Not fasting and never joined = 4

*[If Q35= 1 or 4, skip to Q37]*

Q36. How would you rate for not joining “buka puasa bersama” during the novel coronavirus outbreak?

Extremely difficult = 1

Difficult = 2

Somewhat difficult = 3

Neither easy to do nor difficult = 4

Somewhat easy to do = 5

Easy to do = 6

Very easy to do = 7

Q37. Did you join Eid Mubarak Celebration “Halal bi Halal” with your Muslim friend /Easter Celebration with your Christ friend/Your religion celebration with your family and friend in March-May?

Yes, celebrate it as usual = 1 Yes, but the people were limited = 2

No, celebrate it with family only at home = 3 Never joined celebration = 4

*[If Q37= 1 or 4, skip to Q39]*

Q38. How would you rate for not joining Eid Mubarak Celebration “Halal bi Halal” with your Muslim friend /Easter Celebration with your Christ friend/Your religion celebration with your family and friend during the novel coronavirus outbreak?

Extremely difficult = 1

Difficult = 2

Somewhat difficult = 3

Neither easy to do nor difficult = 4

Somewhat easy to do = 5

Easy to do = 6

Very easy to do = 7

Q39. Did you join a mass exodus “Mudik” during Early Ramadan/Eid Celebration/Easter Celebration to your hometown outside Jakarta Metropolitan Area this year?

Yes = 1 No = 2 Never joined “mudik” = 3

*[If Q39= 1 or 3, skip to Q42]*

Q40. How would you rate for not joining a mass exodus “Mudik” during Early Ramadan/Eid Celebration/Easter Celebration to your hometown during the novel coronavirus outbreak?

Extremely difficult = 1

Difficult = 2

Somewhat difficult = 3

Neither easy to do nor difficult = 4

Somewhat easy to do = 5

Easy to do = 6

Very easy to do = 7

**Part III: Qualitative part participation**

Q41. I would like to invite you to participate in the qualitative part of this research later in the next a few weeks. The qualitative part will be conducted by using Photovoice and Online Focus Group Discussion. Would you like to participate?

Yes = 1 No = 0

*[If Q41= 0, skip to End of questionnaire]*

*[Qualitative methods]*

*[Photovoice is a qualitative method that provide an opportunity to describe and share your experience through a photo that is difficult to tell by words only. Online Focus Group Discussions (Online FGDs) is a method to get an understanding about research topic. Like FGD in general, Online FGDs in this study will produce a view and different opinion through interaction between participants in a group that will be moderated]*

Q42. Which qualitative method that you interested to join?

Photovoice = 1

Online FGDs = 2

Q43. Thank you for your intention to participate in qualitative part of this research. I would like to ask your contact information. I will contact you to explain more detail about this qualitative part. Please choose type of contact information that you want to share:

Whatsapp/Line/Email (specify):___________________________________

This is the end of questionnaire. Thank you very much for your time.
