# Supplementary figures and images for: Factors influencing physical distancing compliance among young adults during COVID-19 pandemic in Indonesia: A photovoice mixed methods study
Source: PLOS Glob Public Health. 2022 Jan 13;2(1):e0000035. doi: 10.1371/journal.pgph.0000035 (PMC10021510; doi:10.1371/journal.pgph.0000035)

**S2 Questionnaire. Compliance questionnaire (Indonesian)**

**
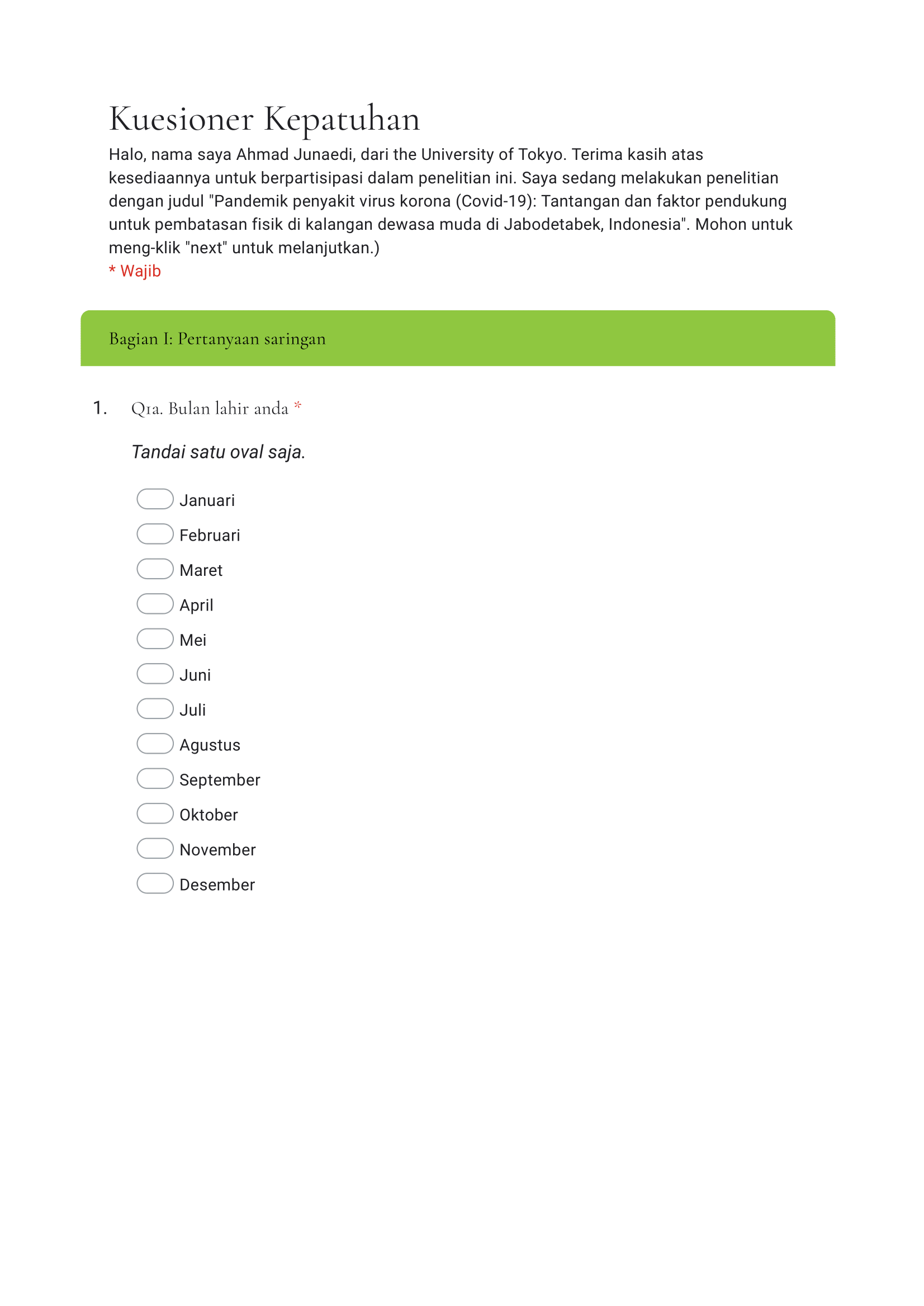
**


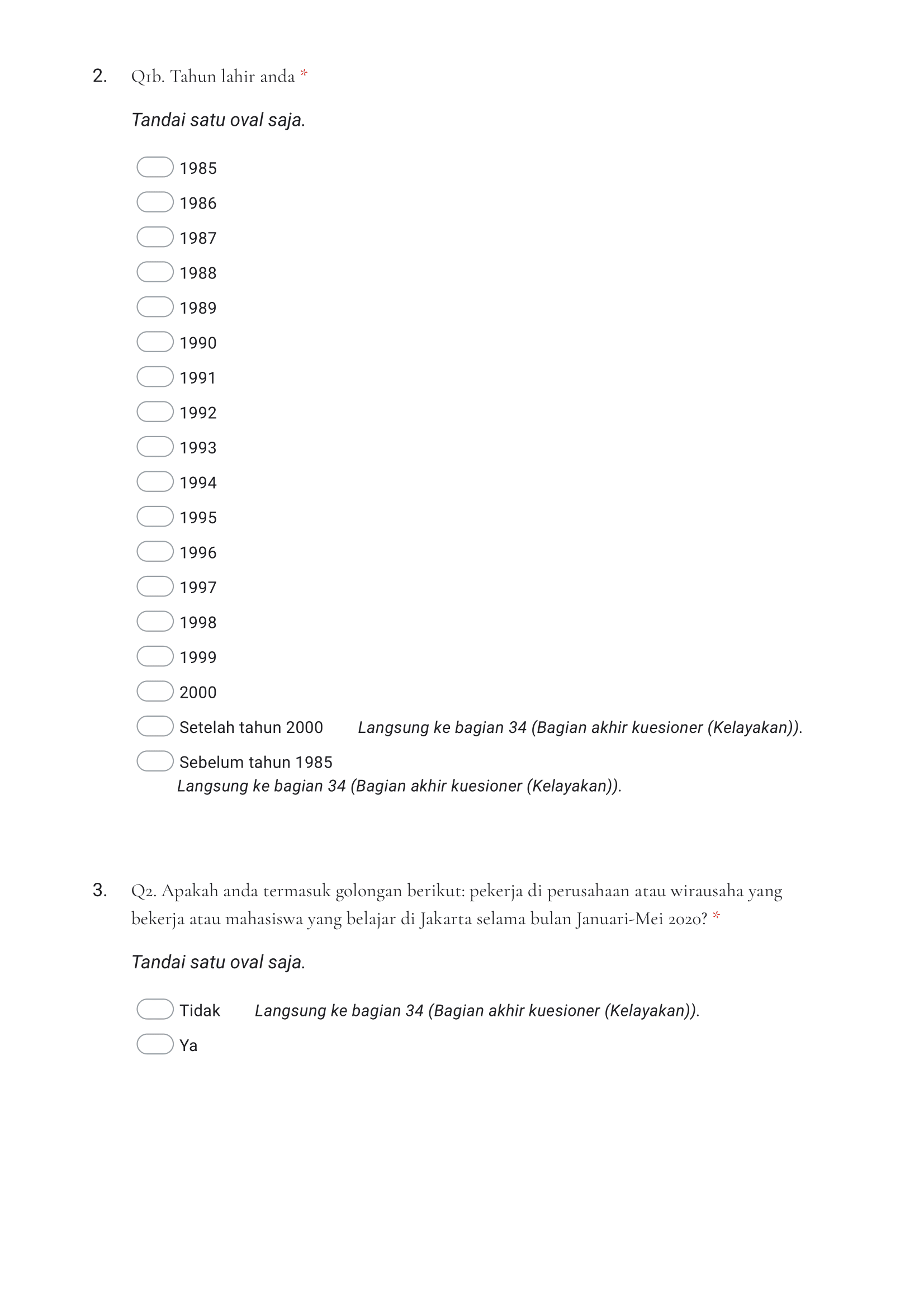


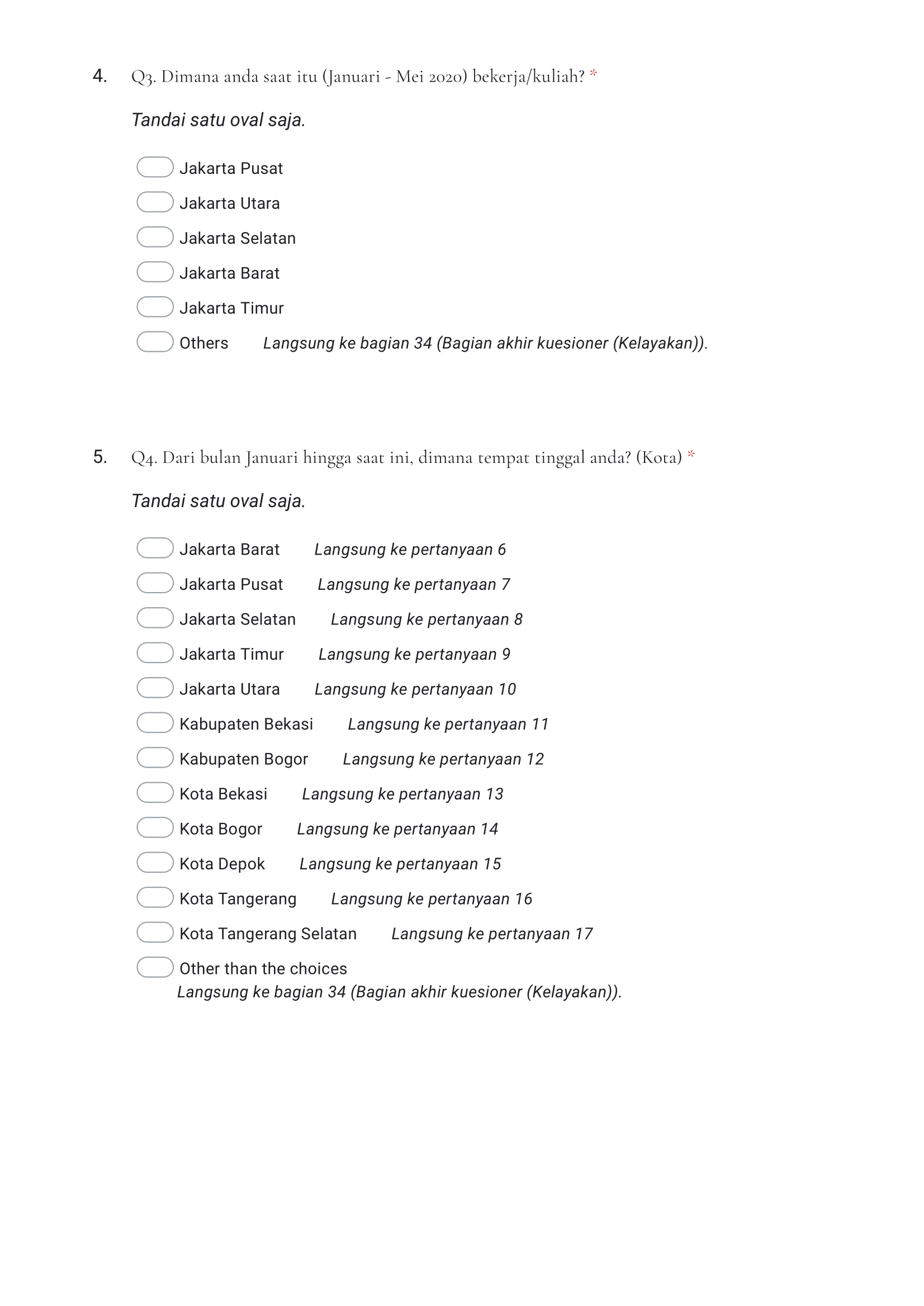


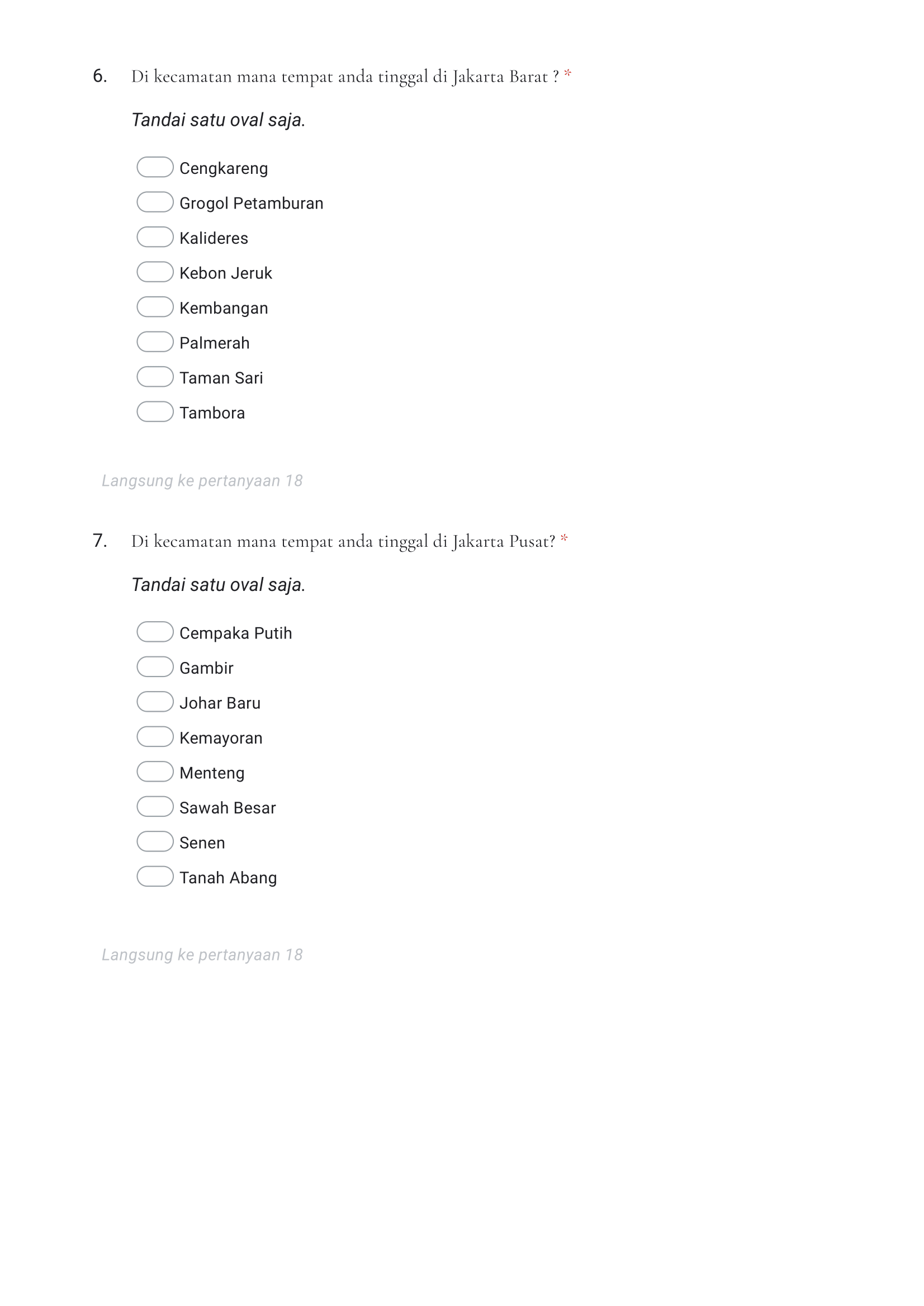


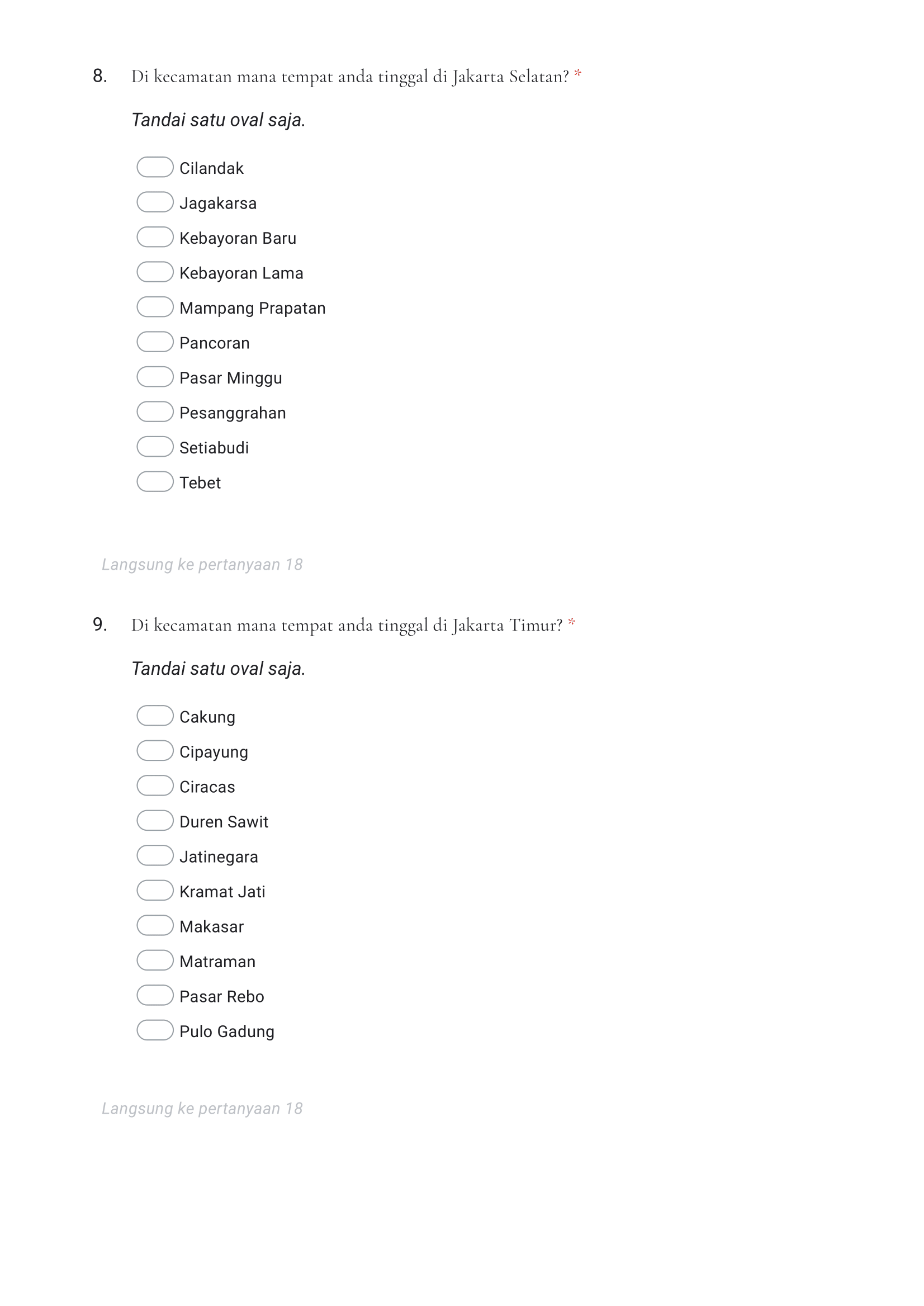


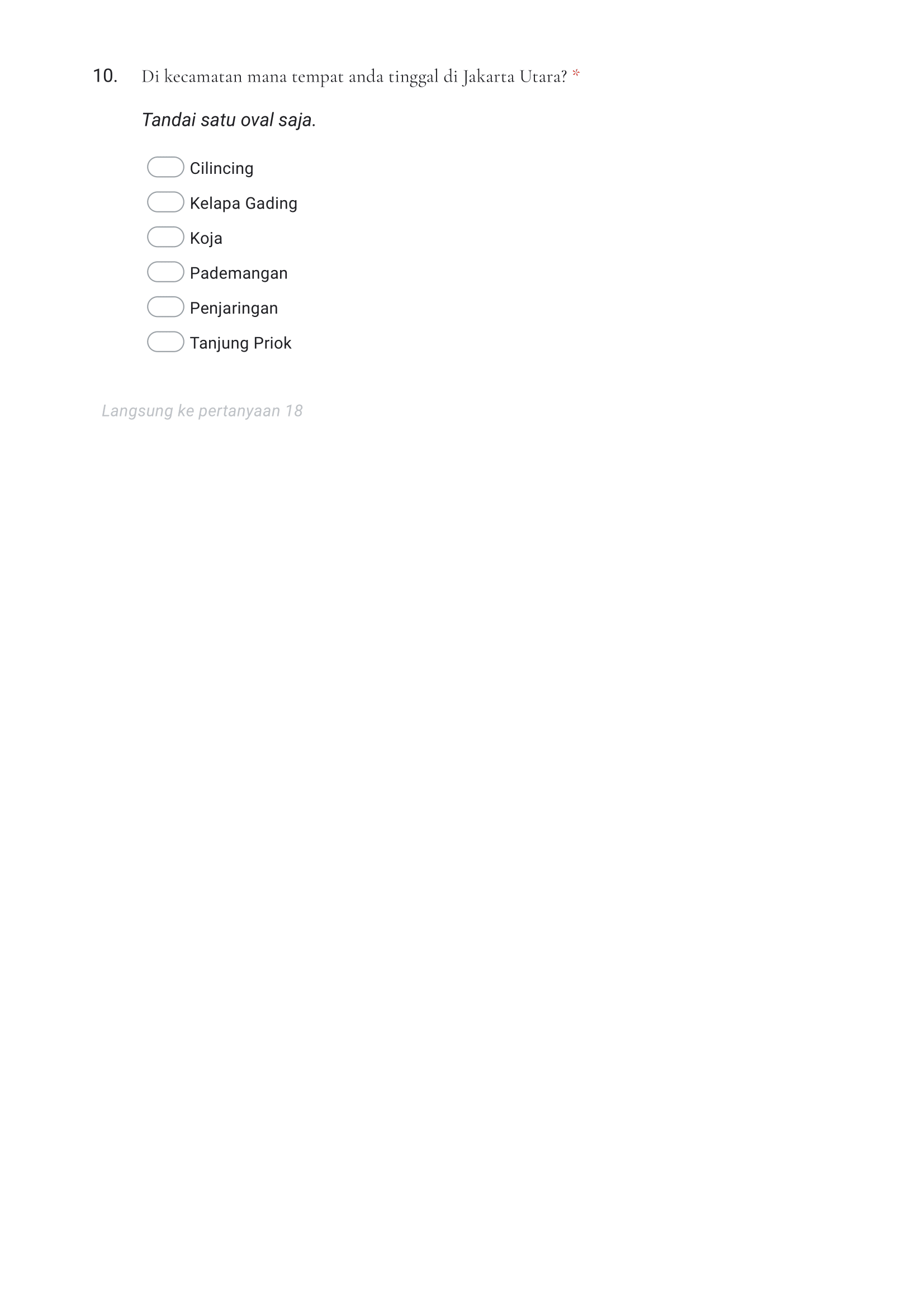

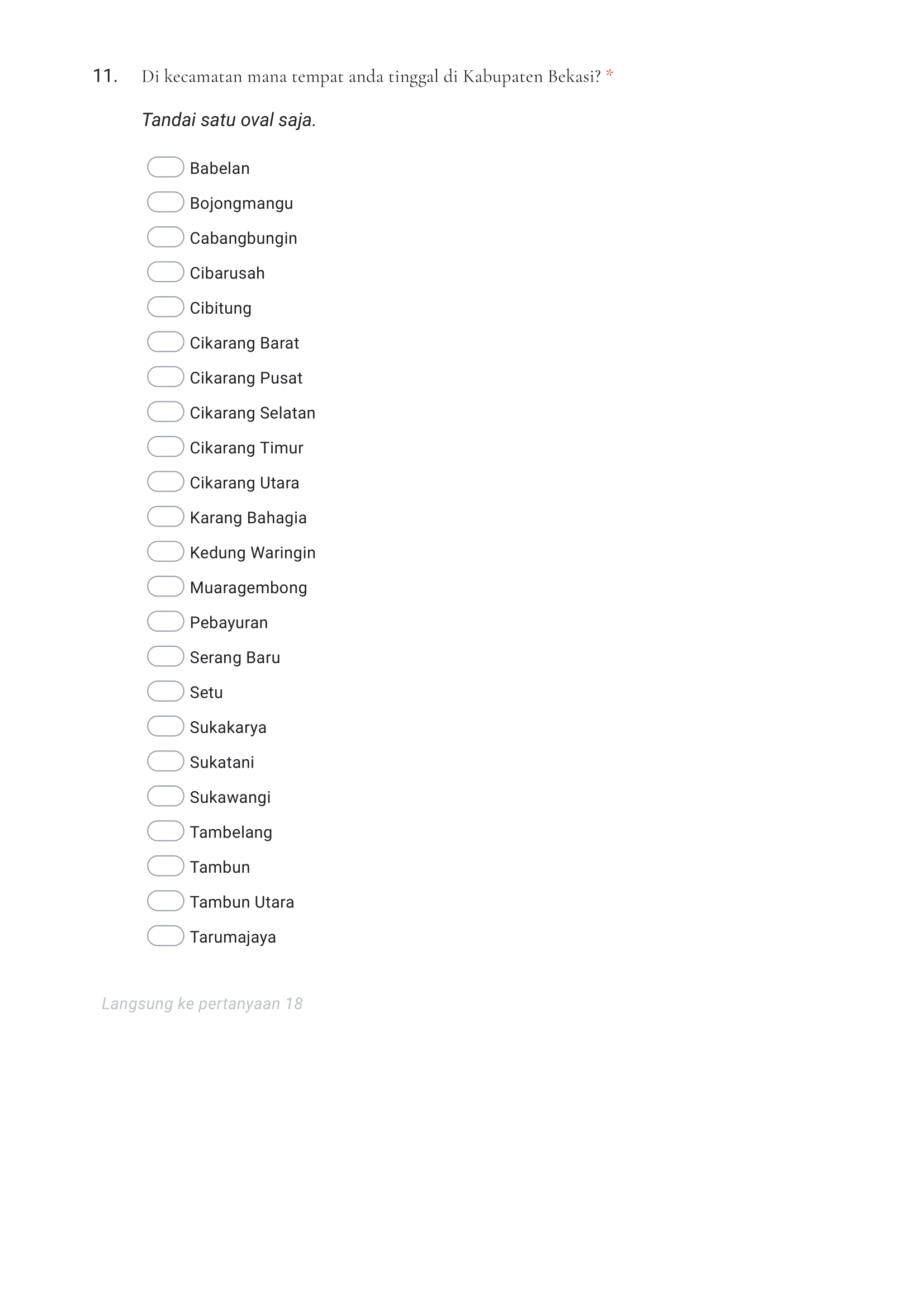

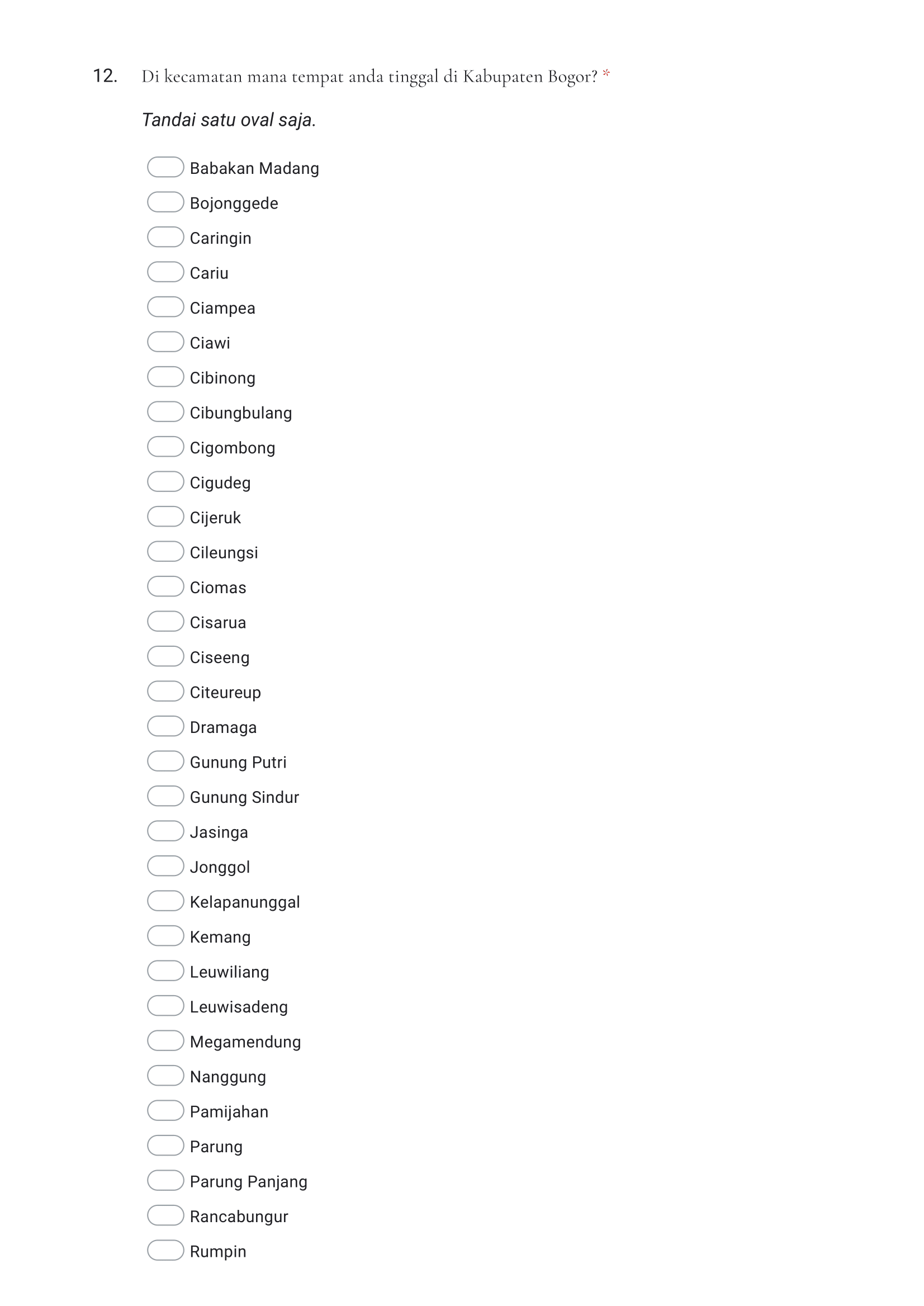

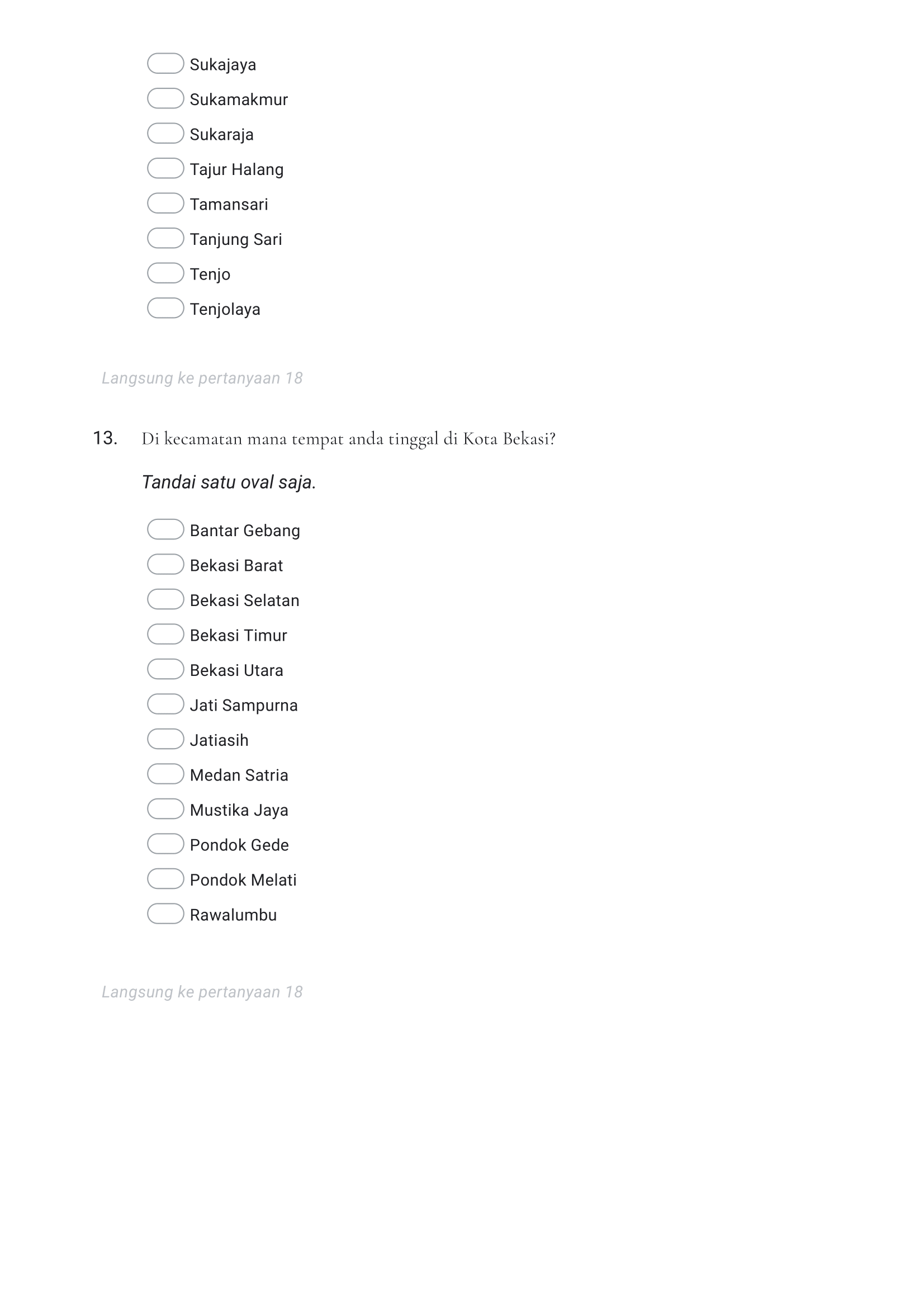

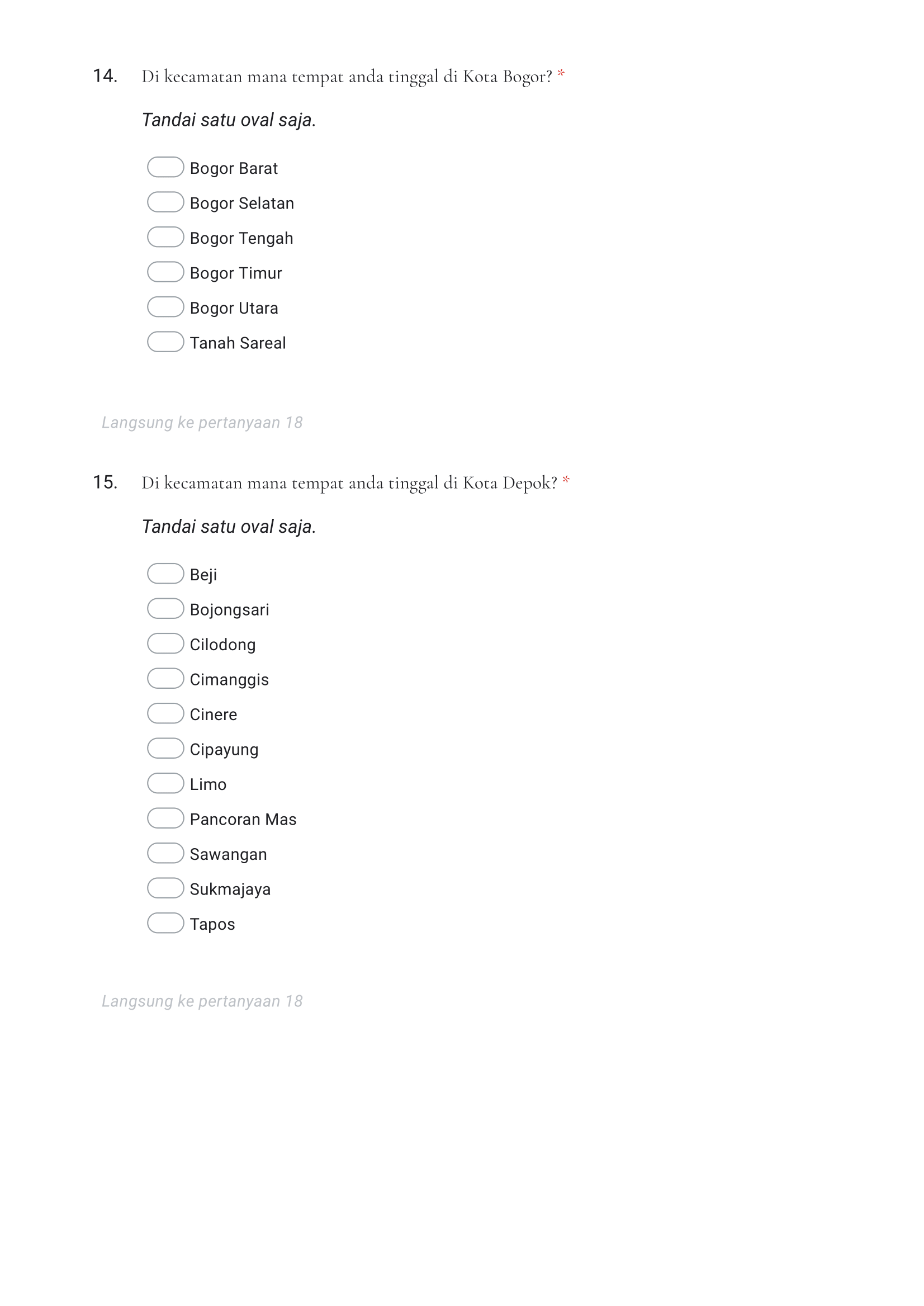

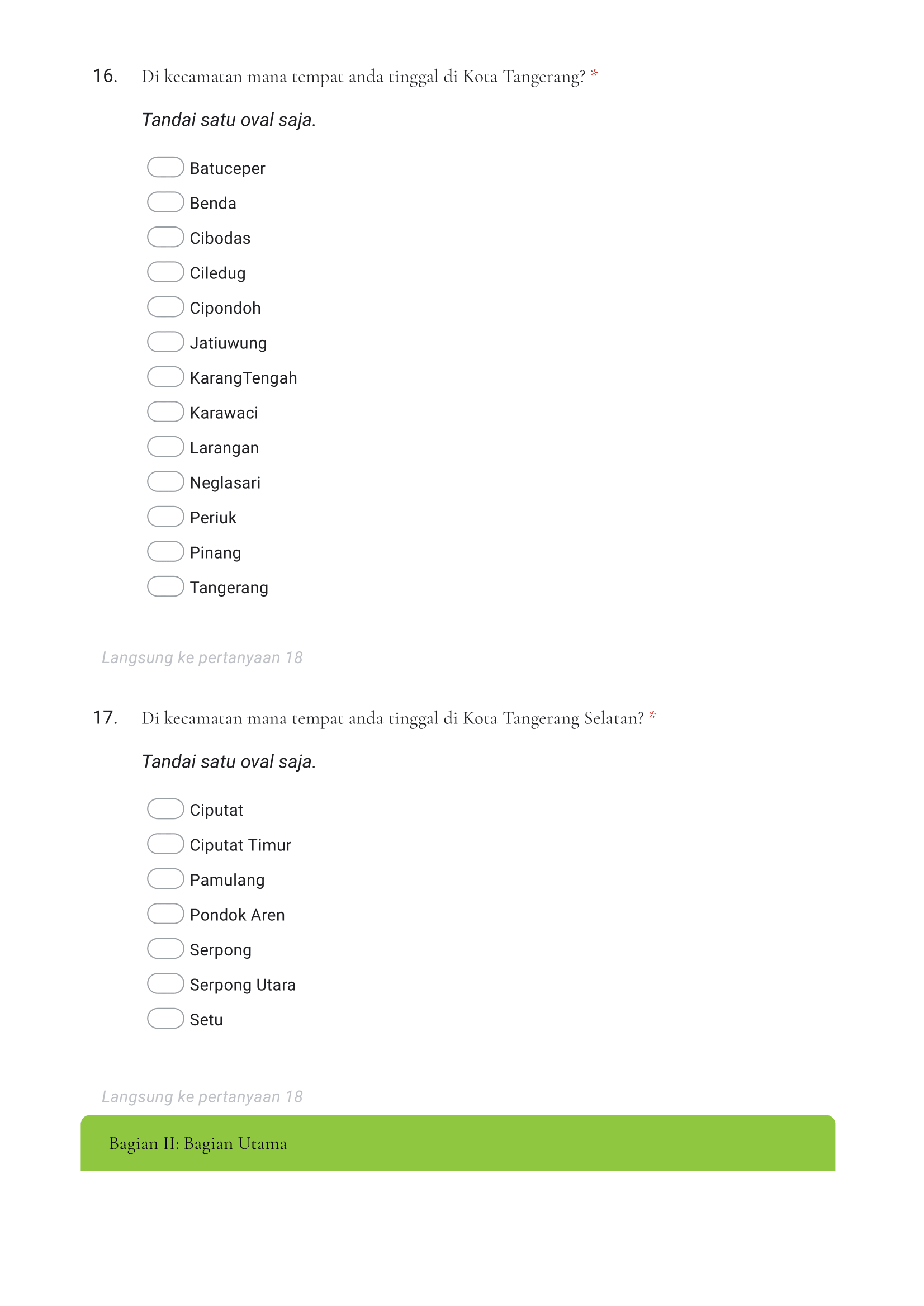

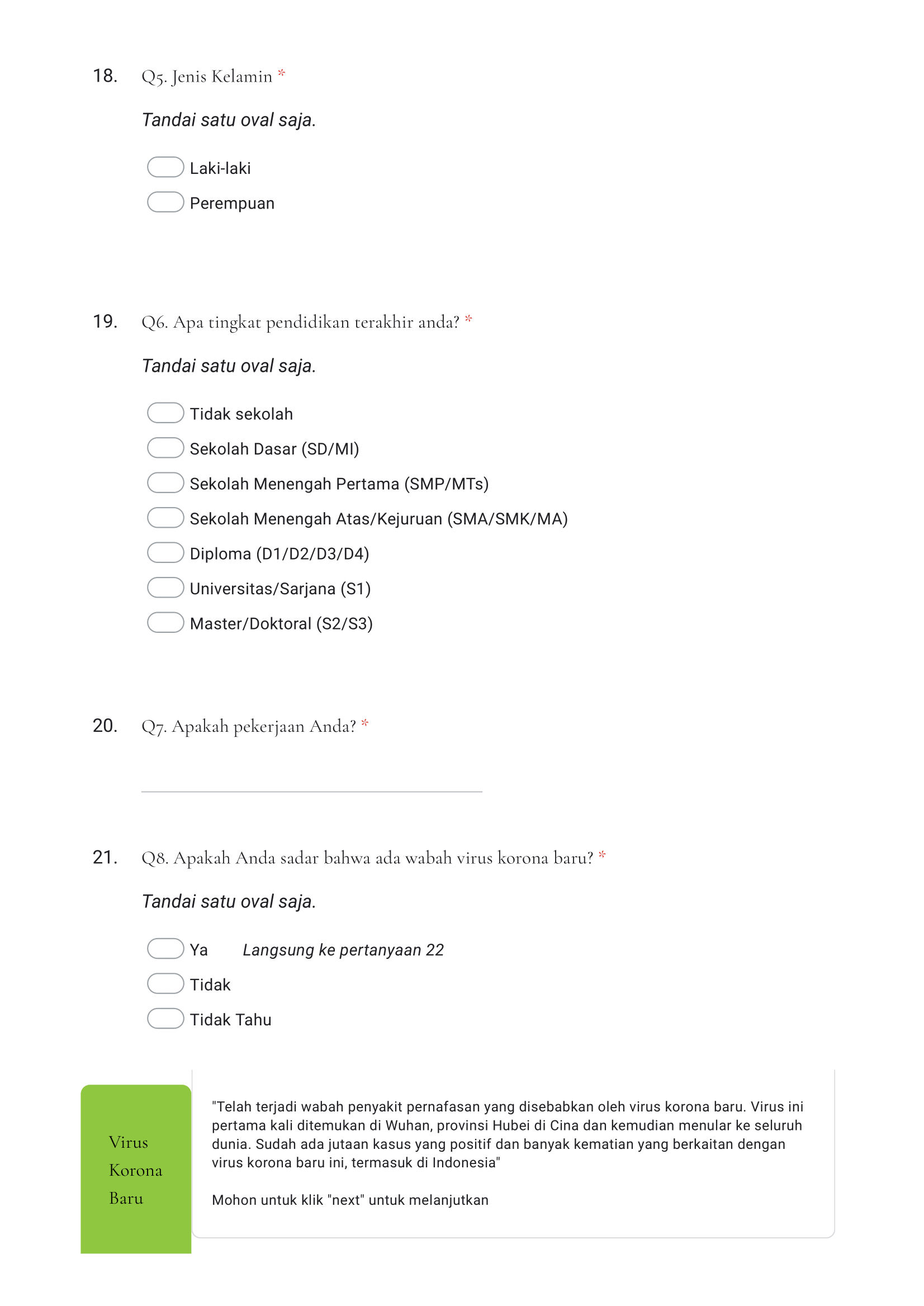

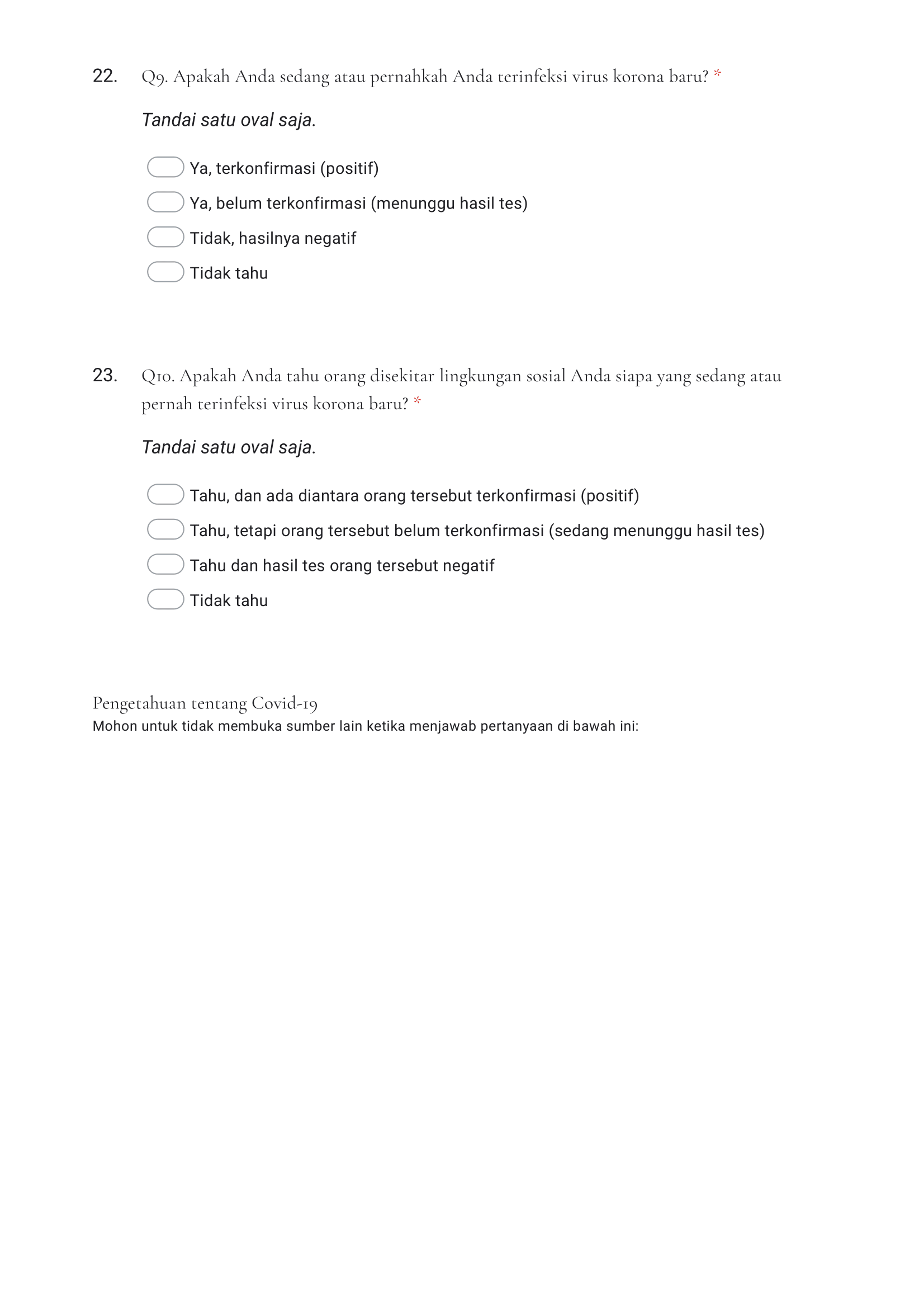

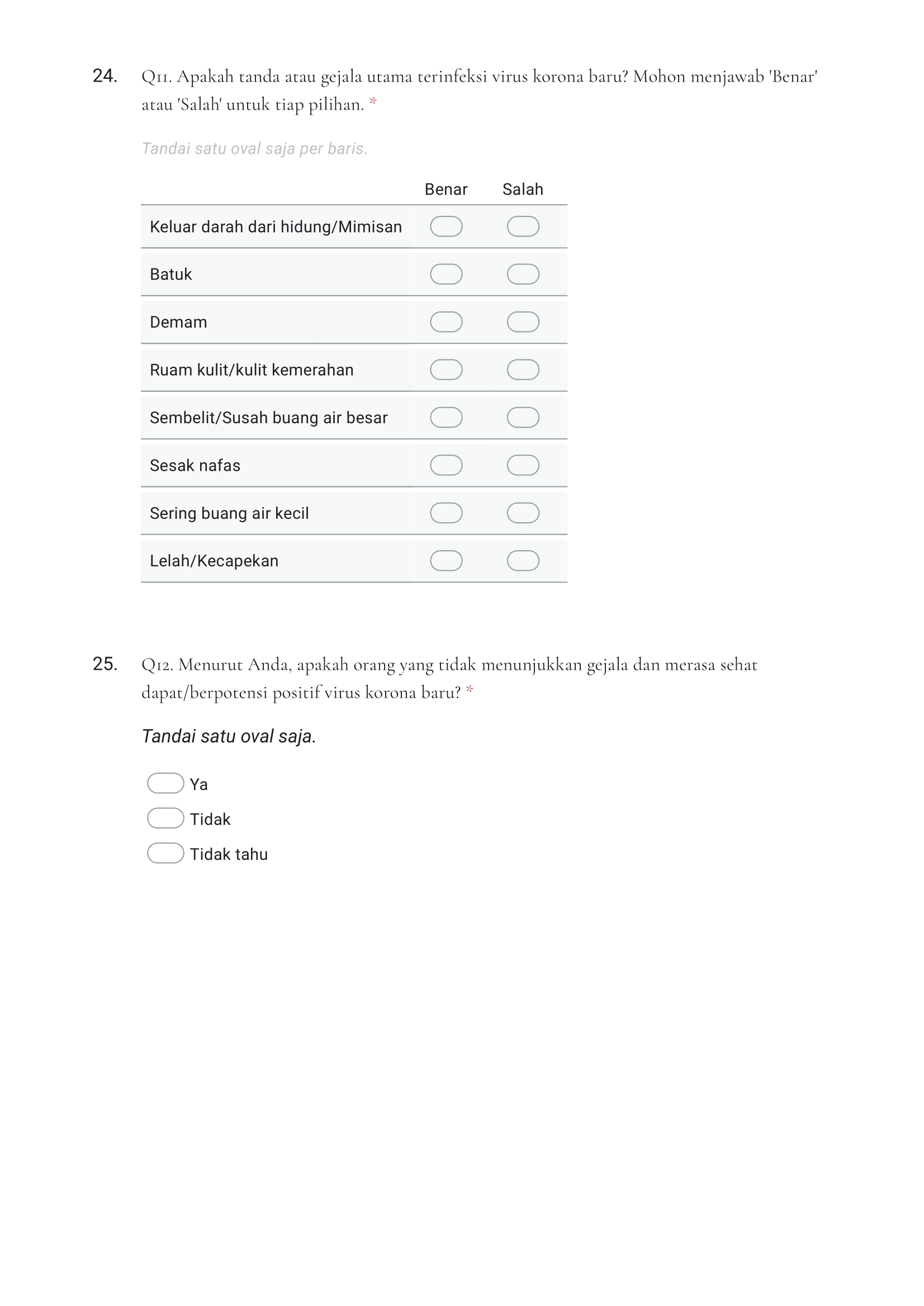

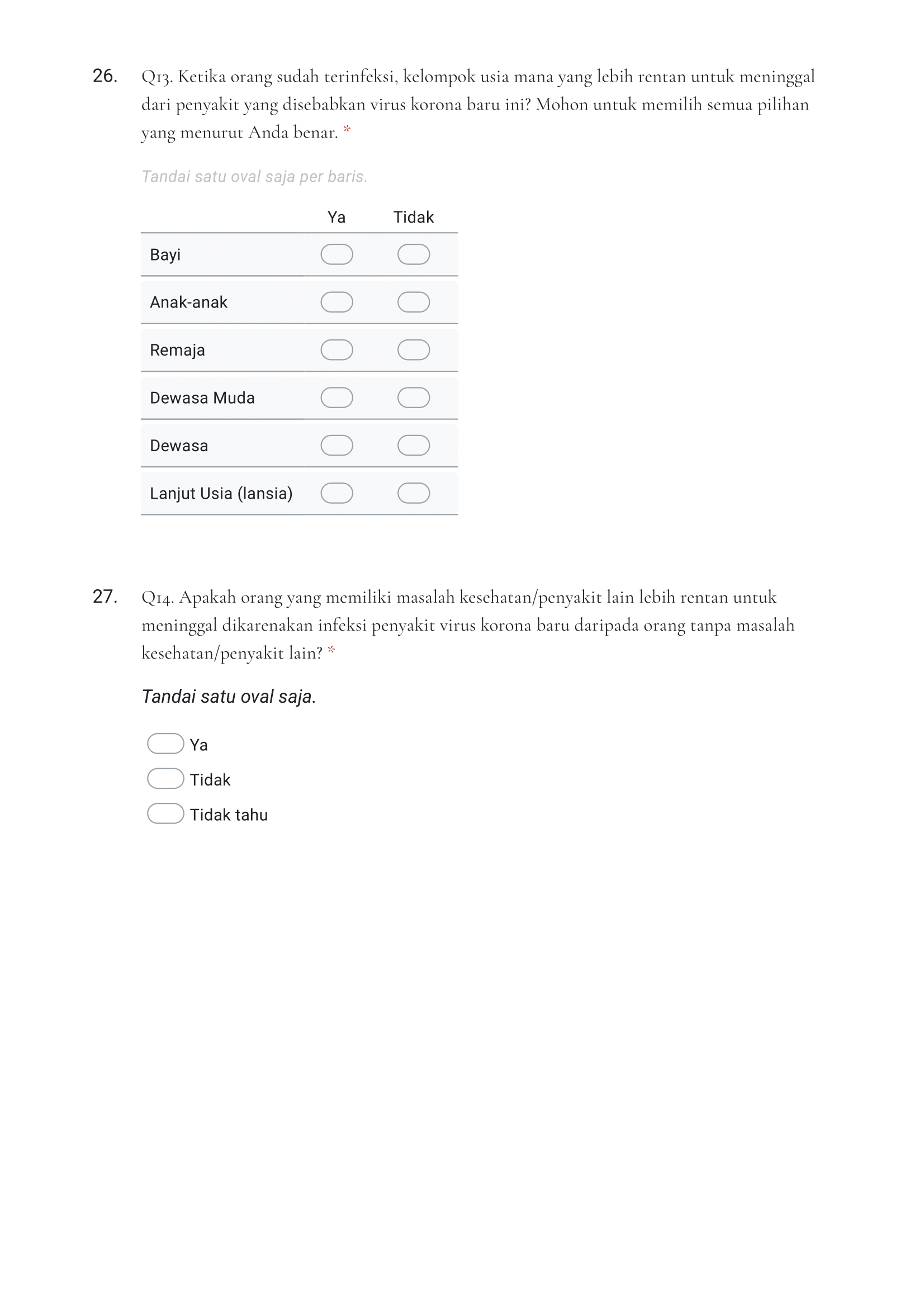

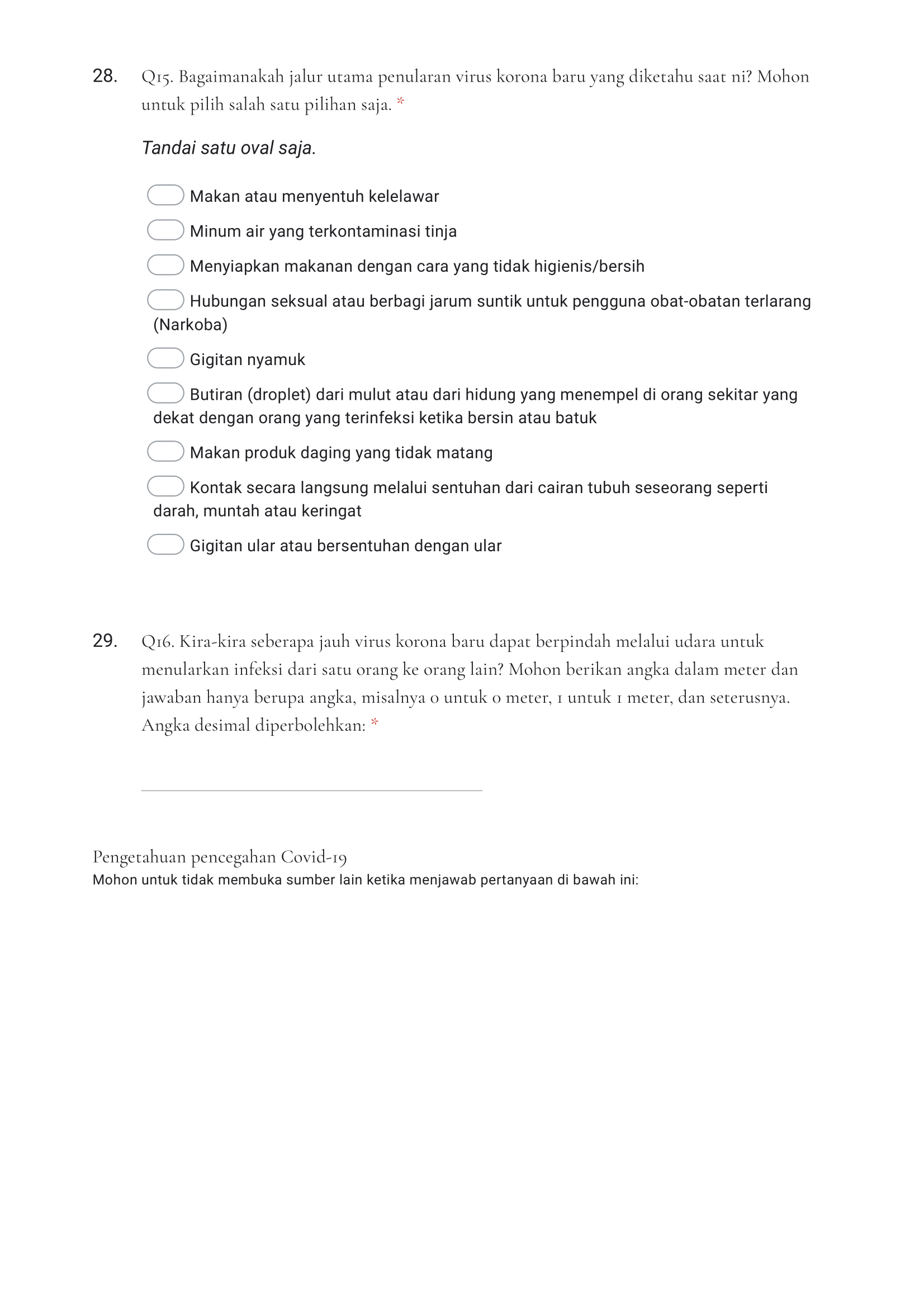

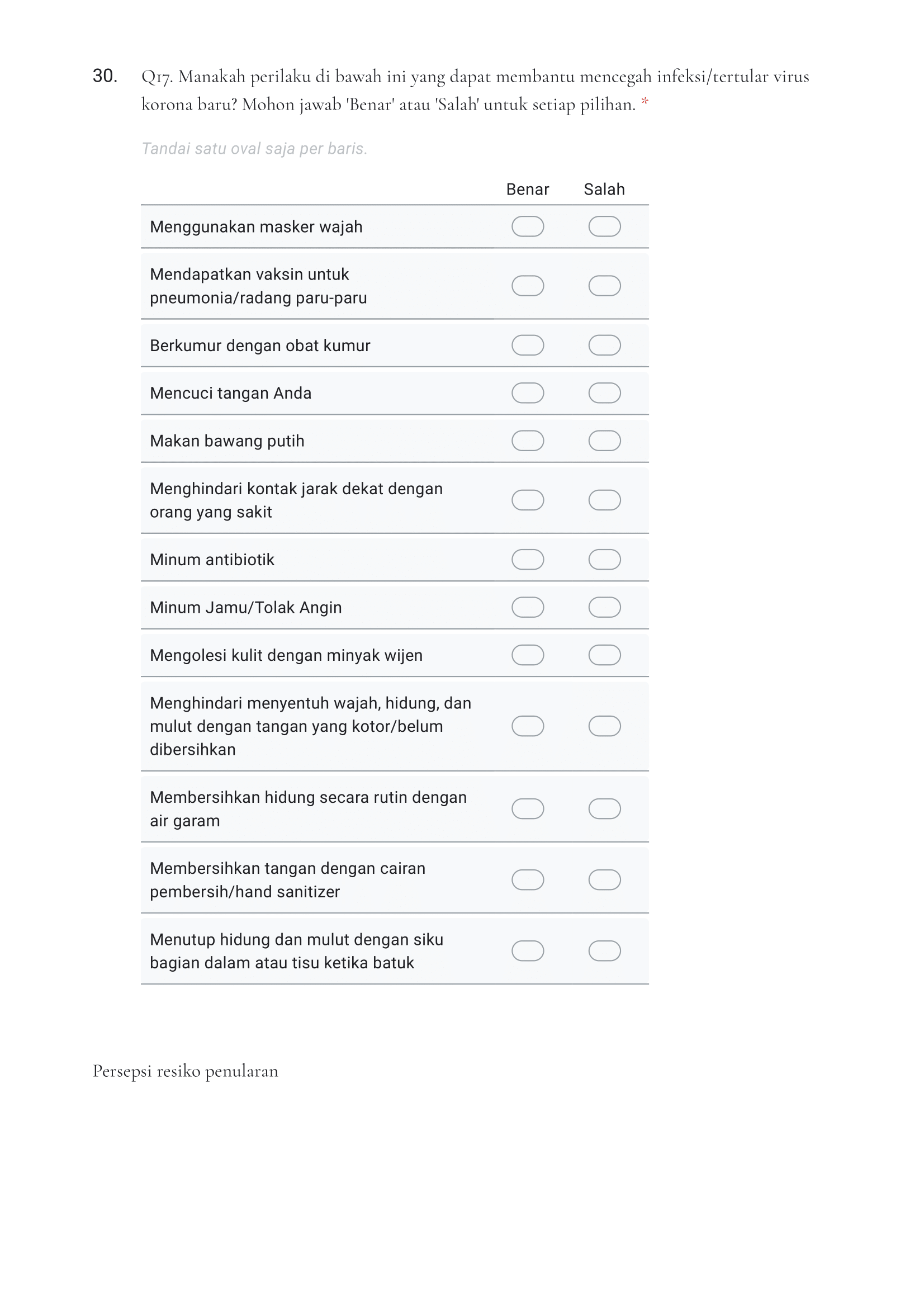

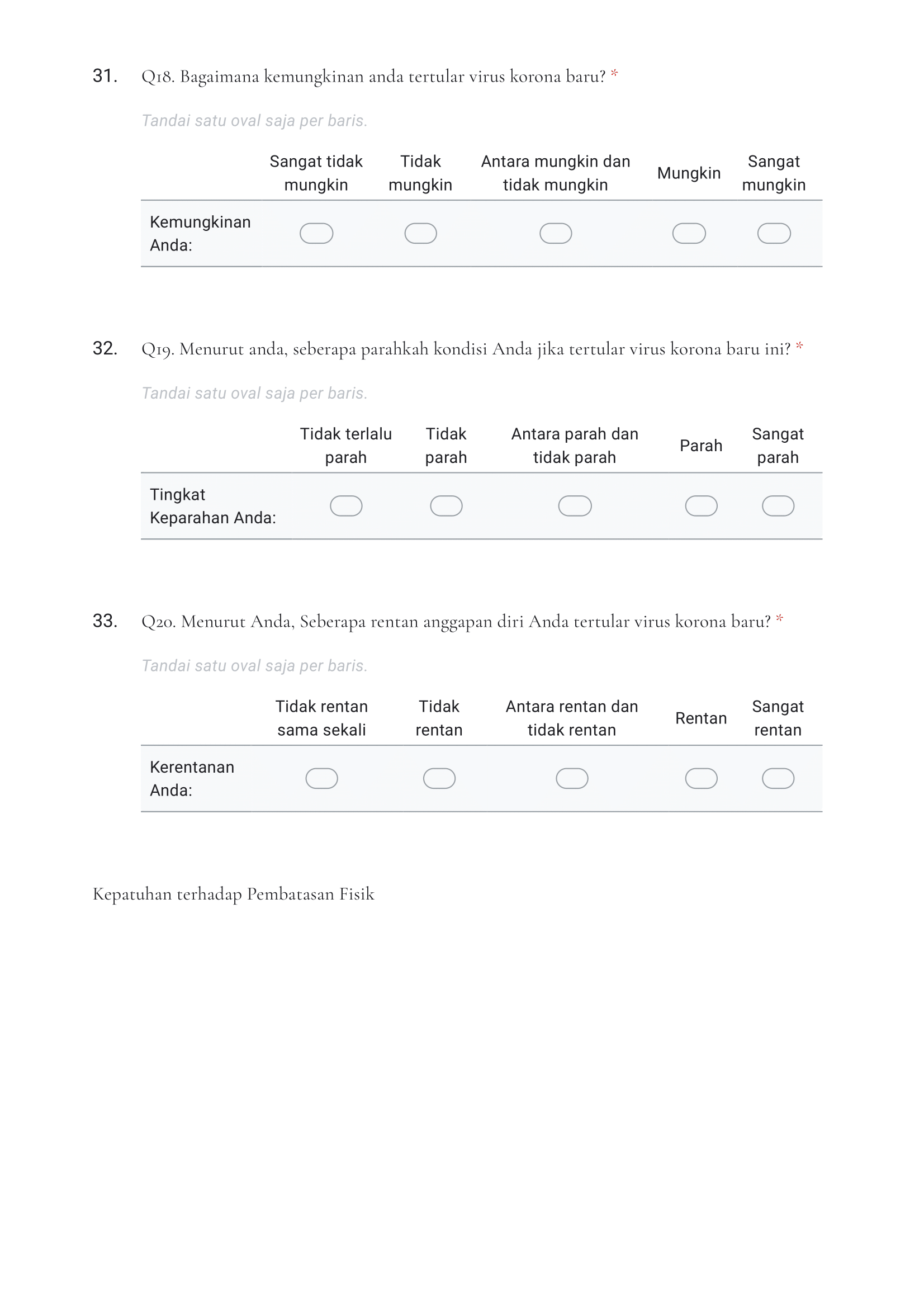

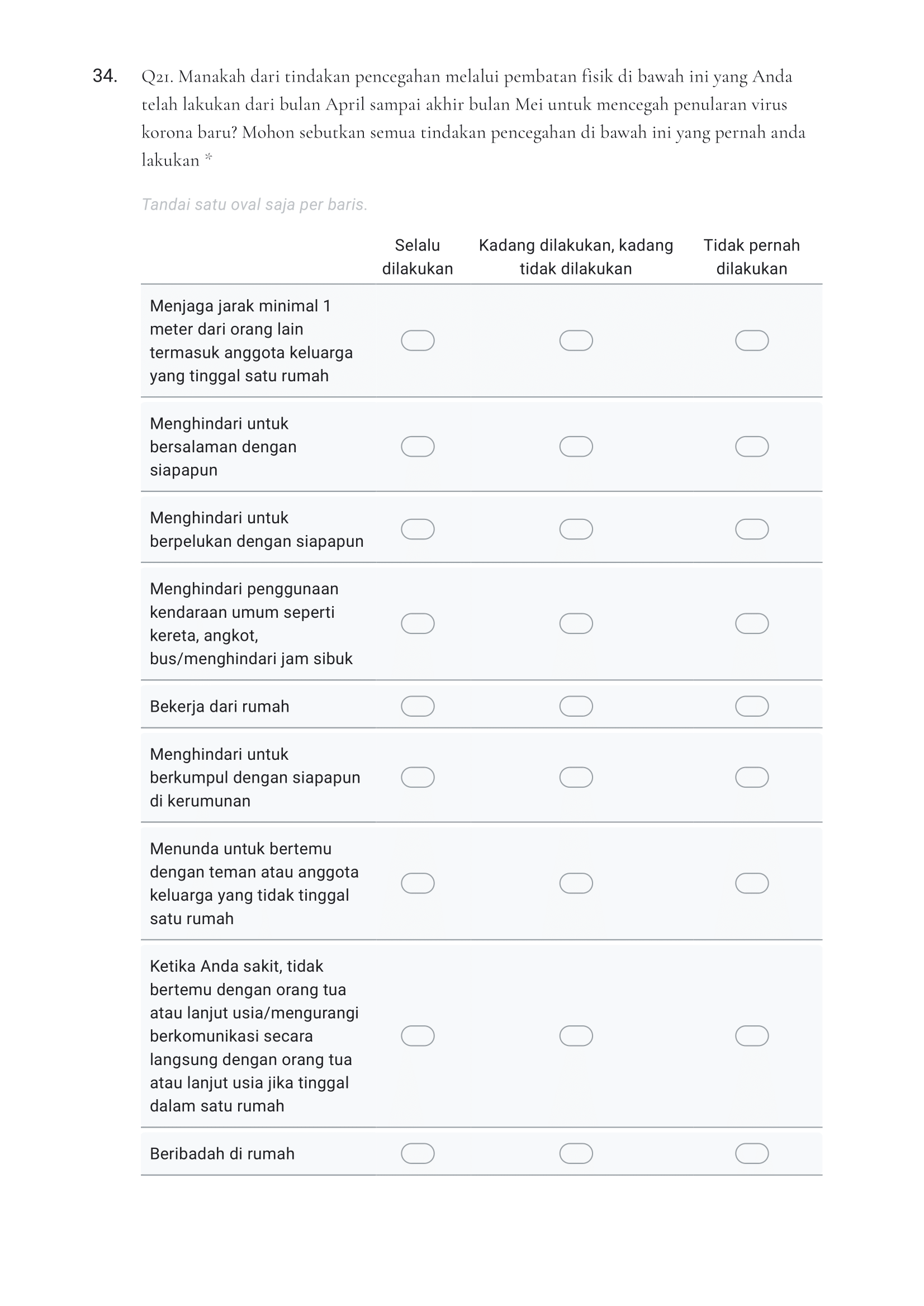

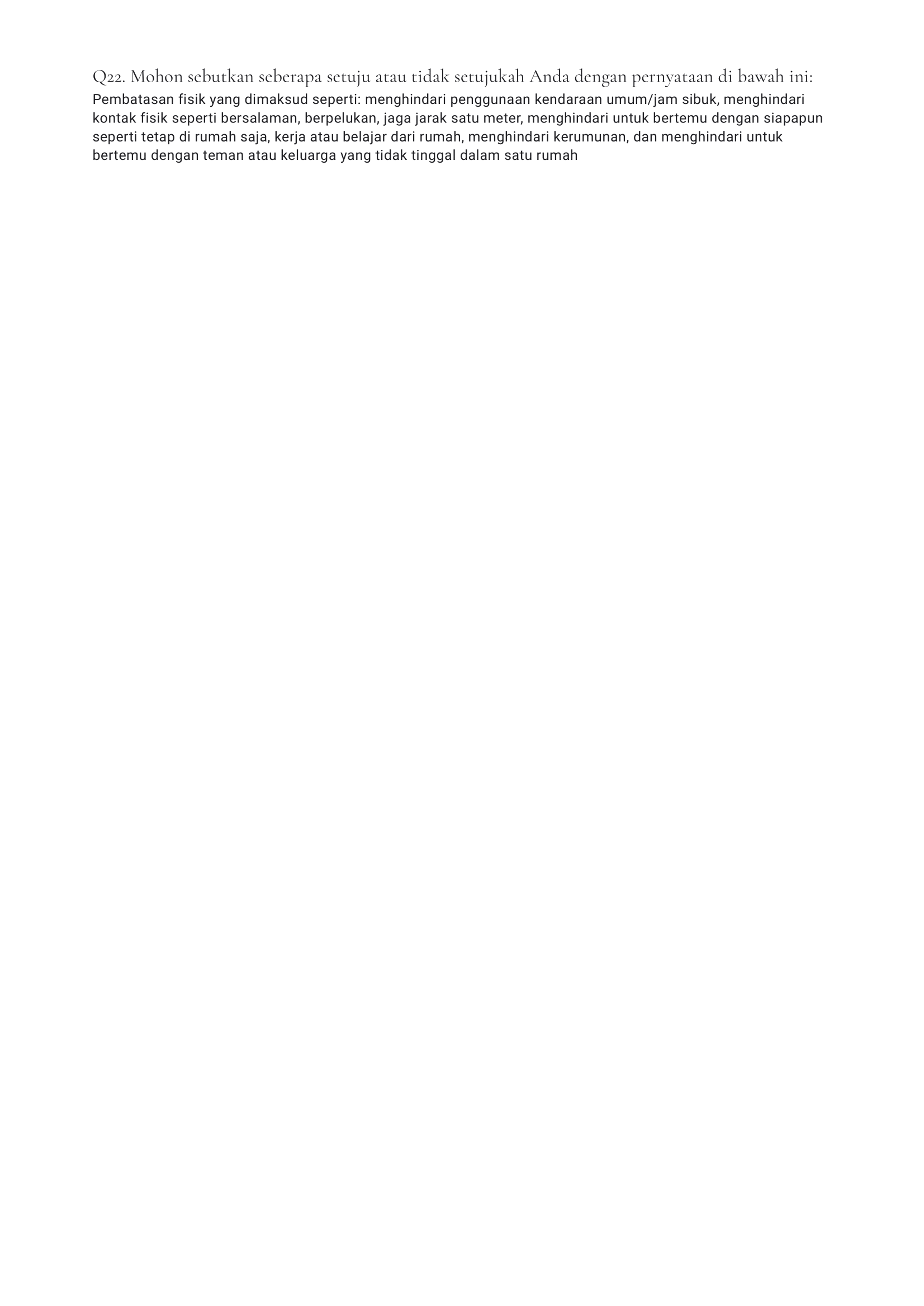

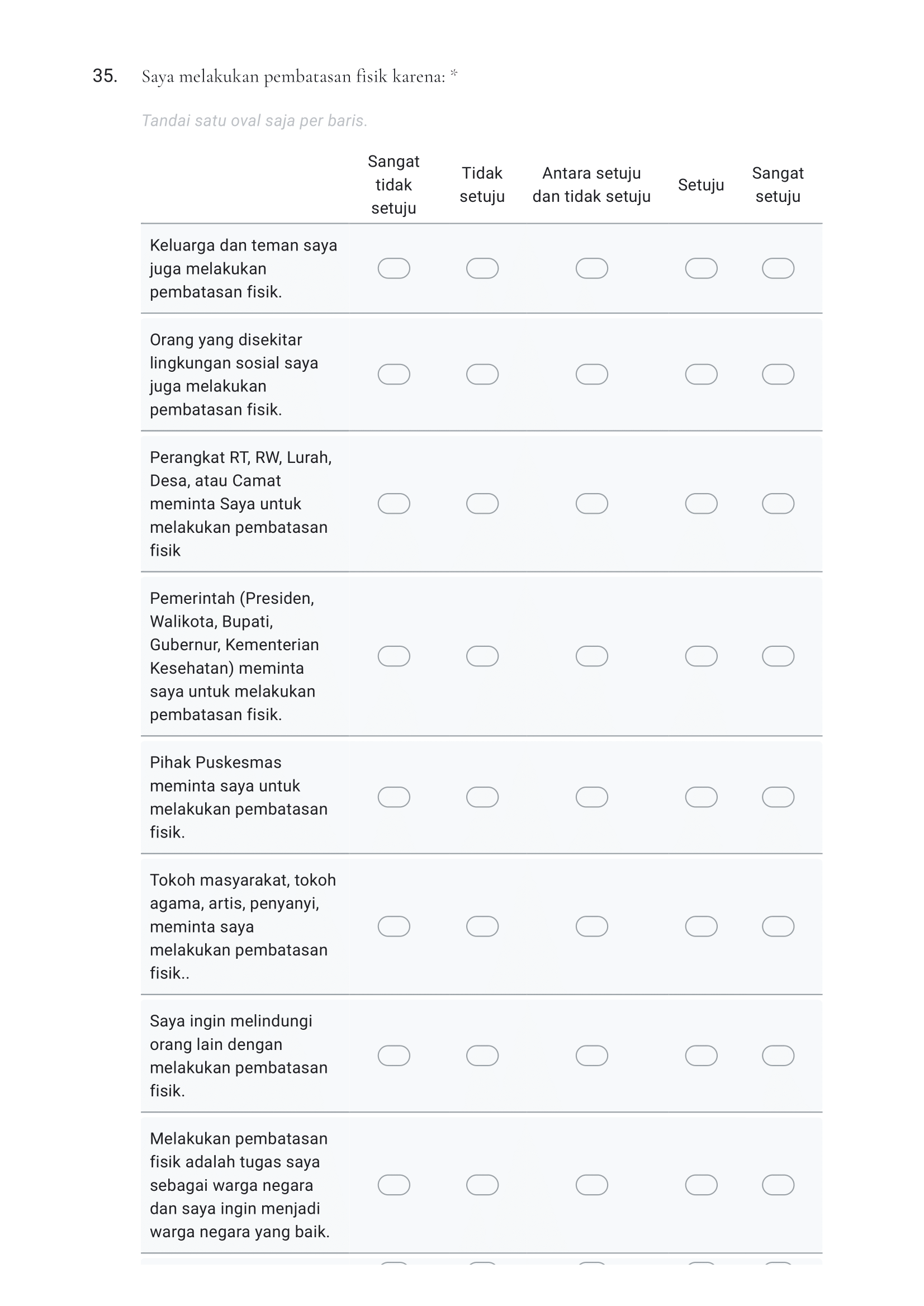

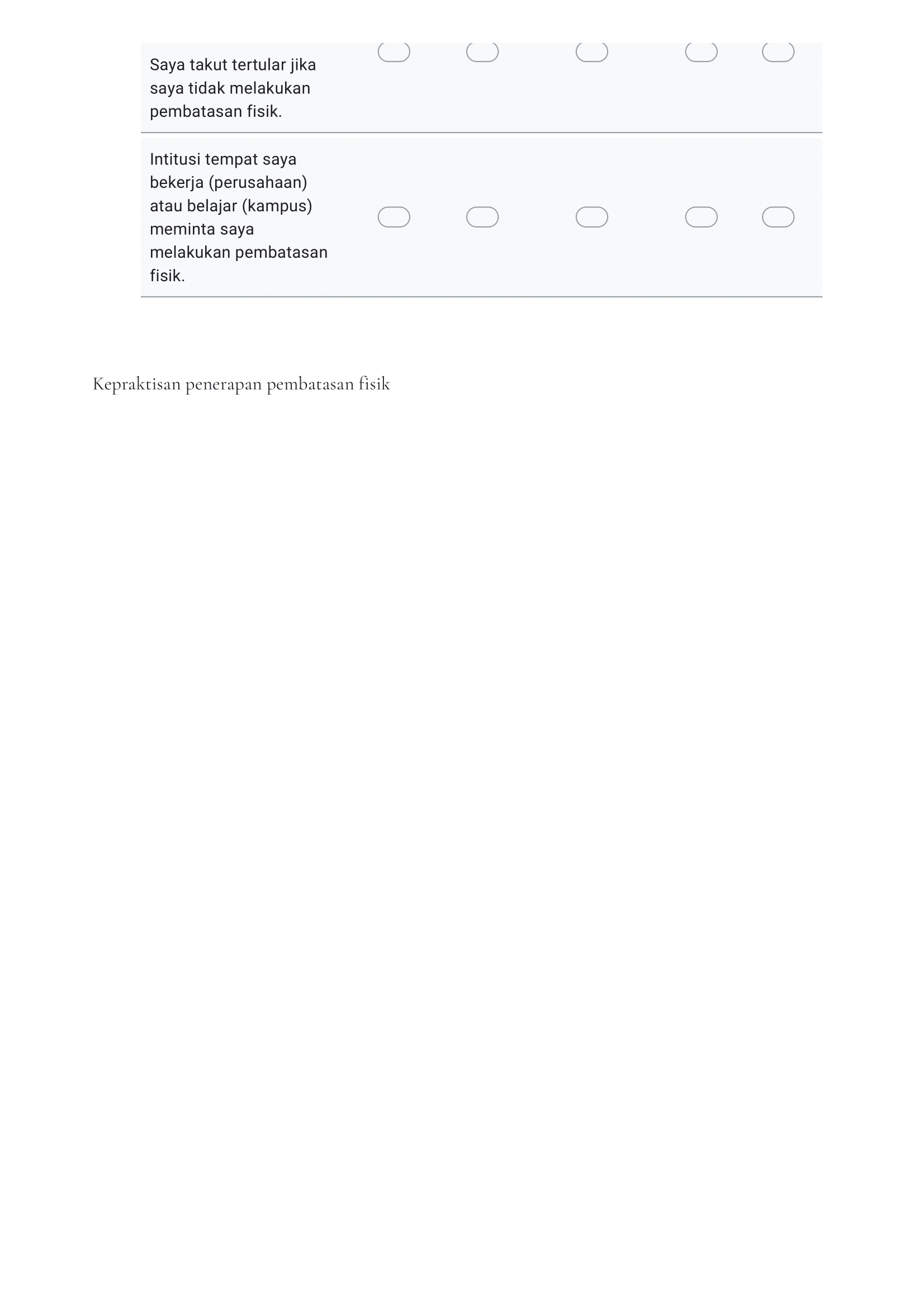

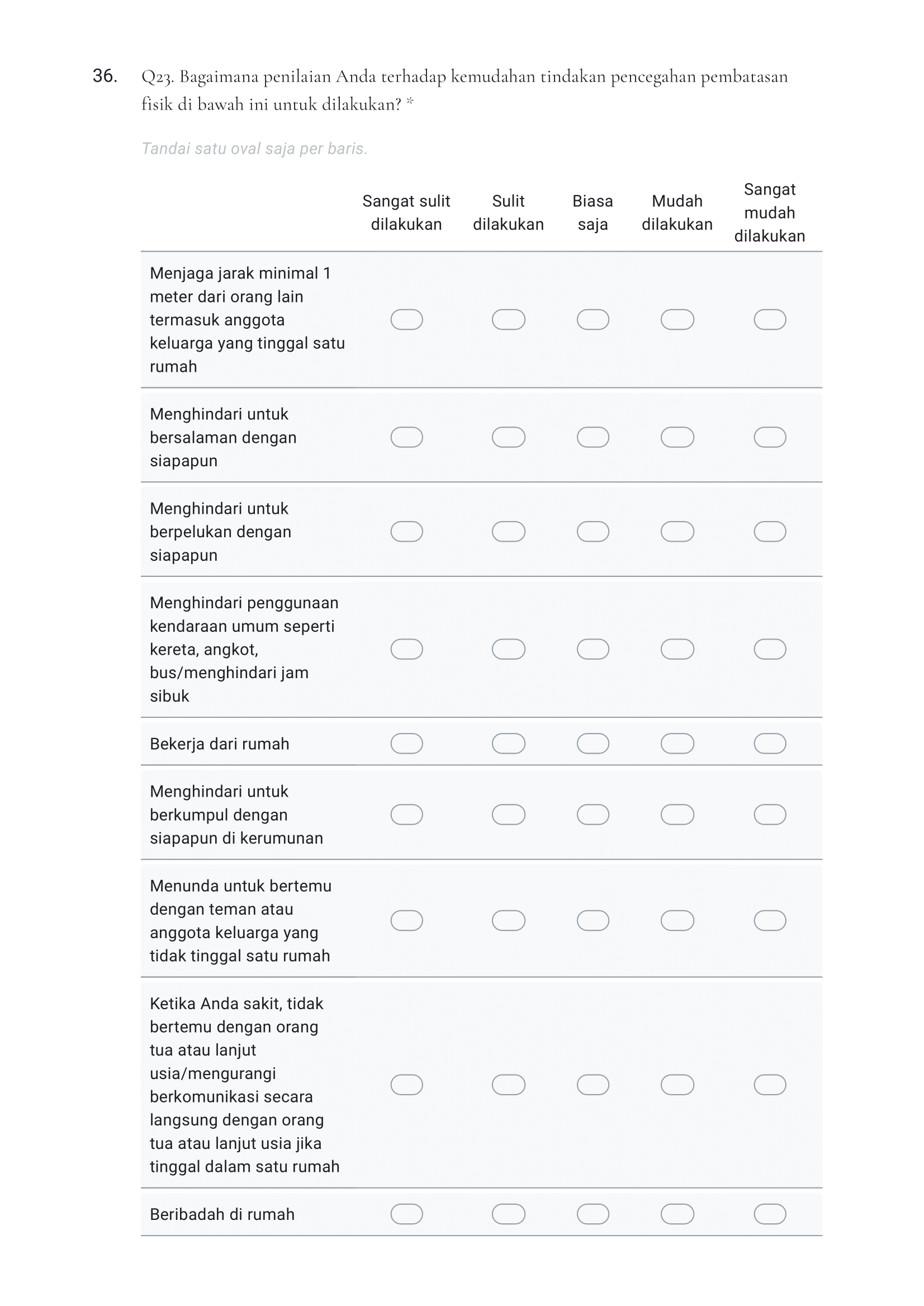

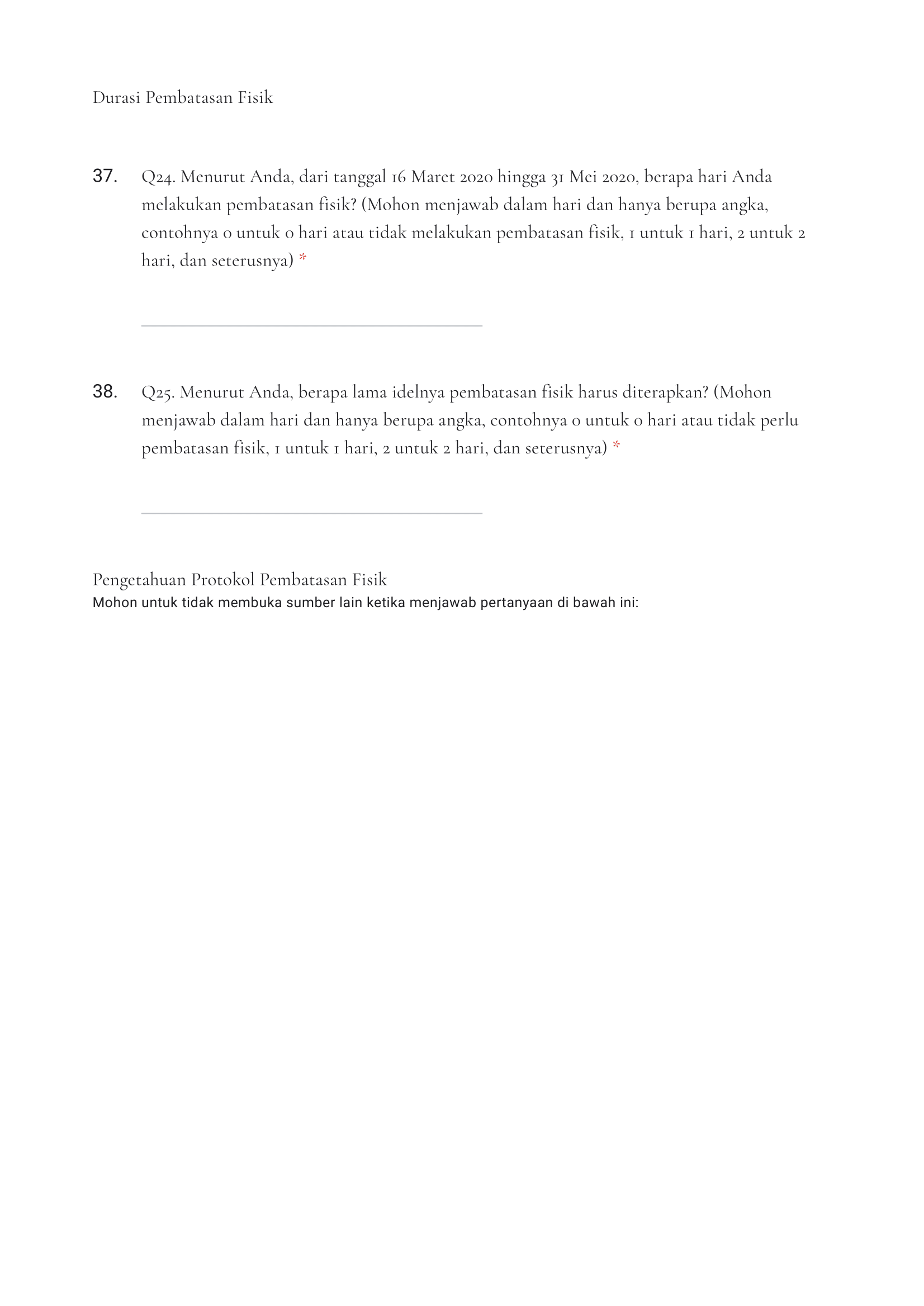

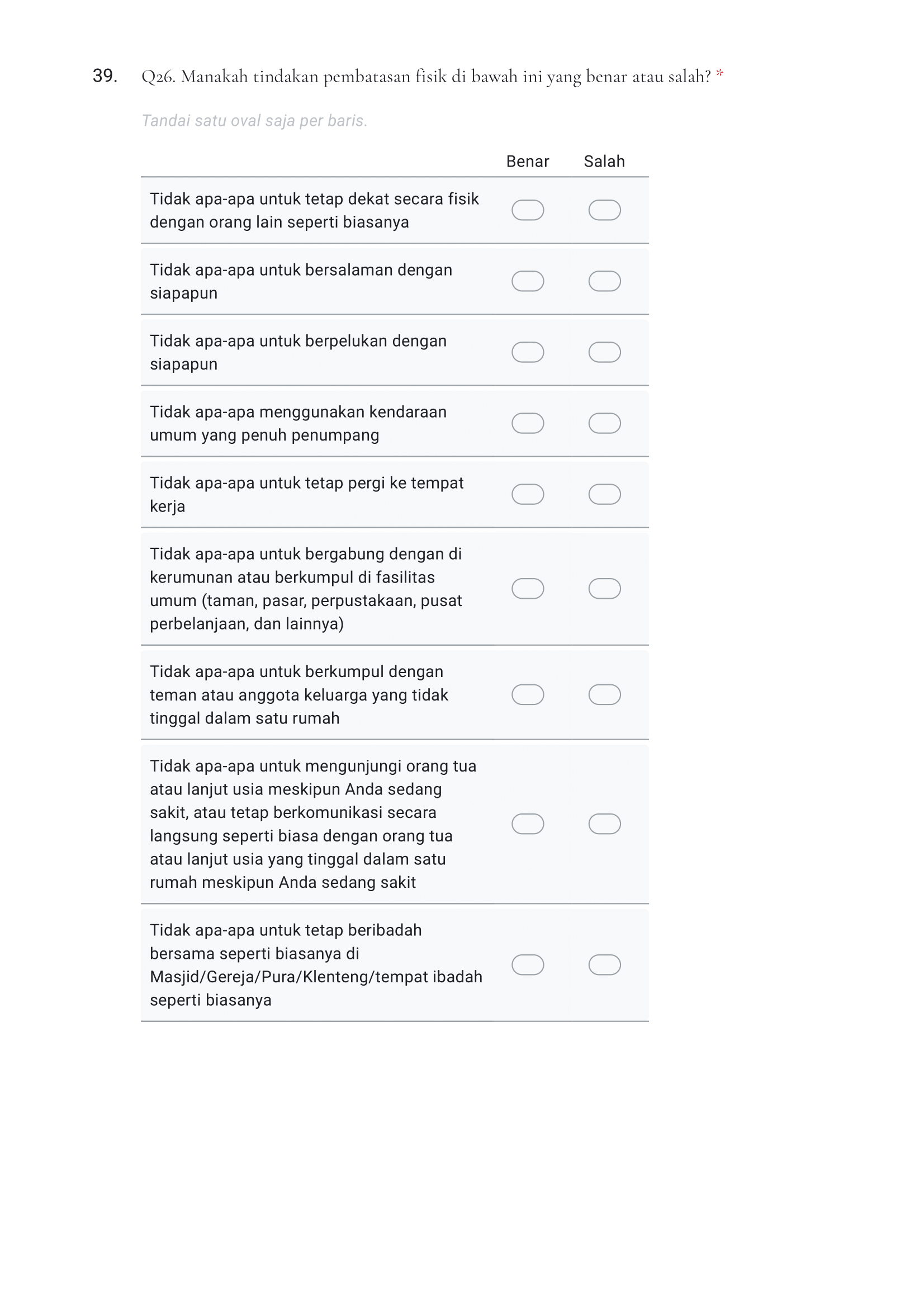

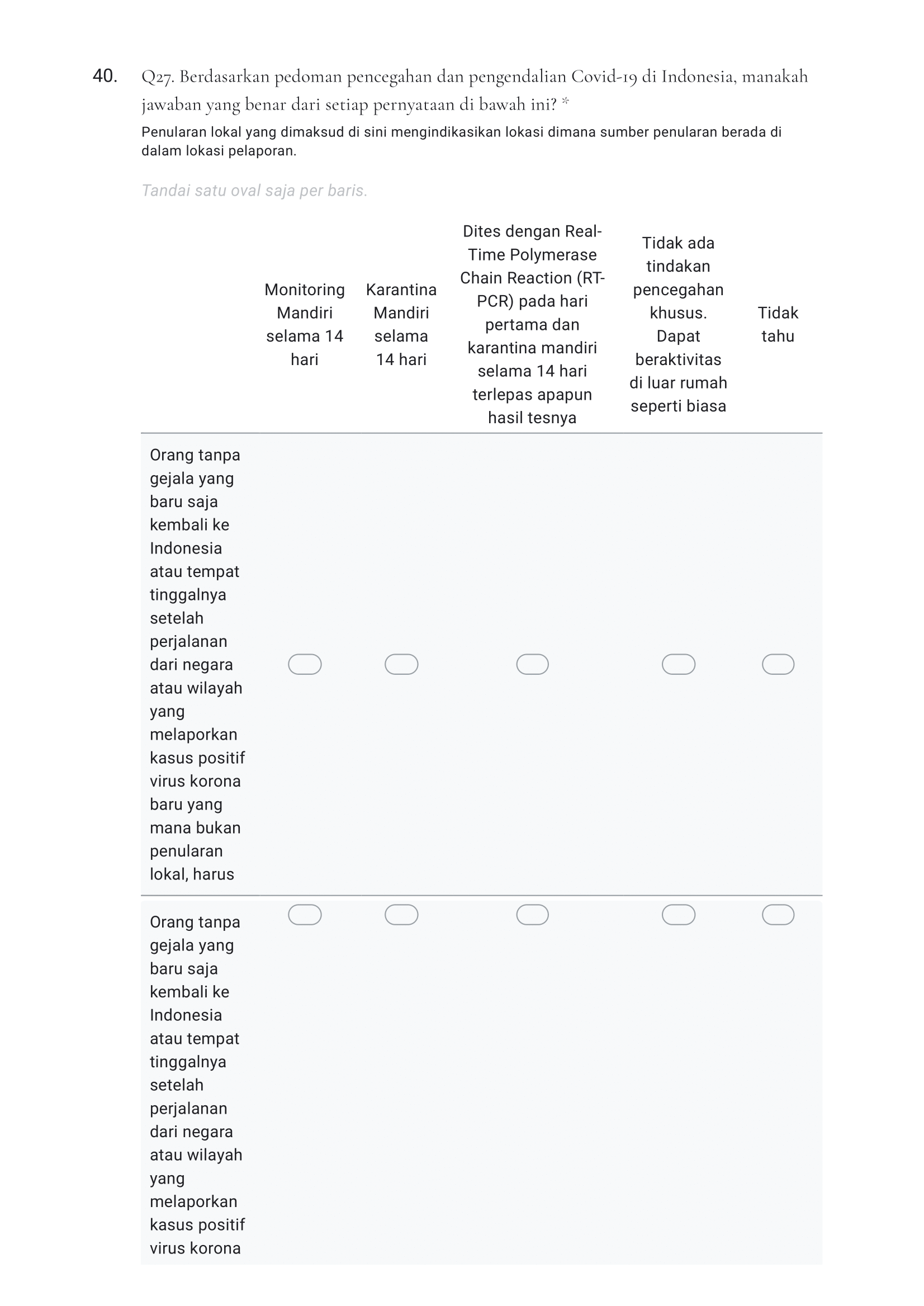

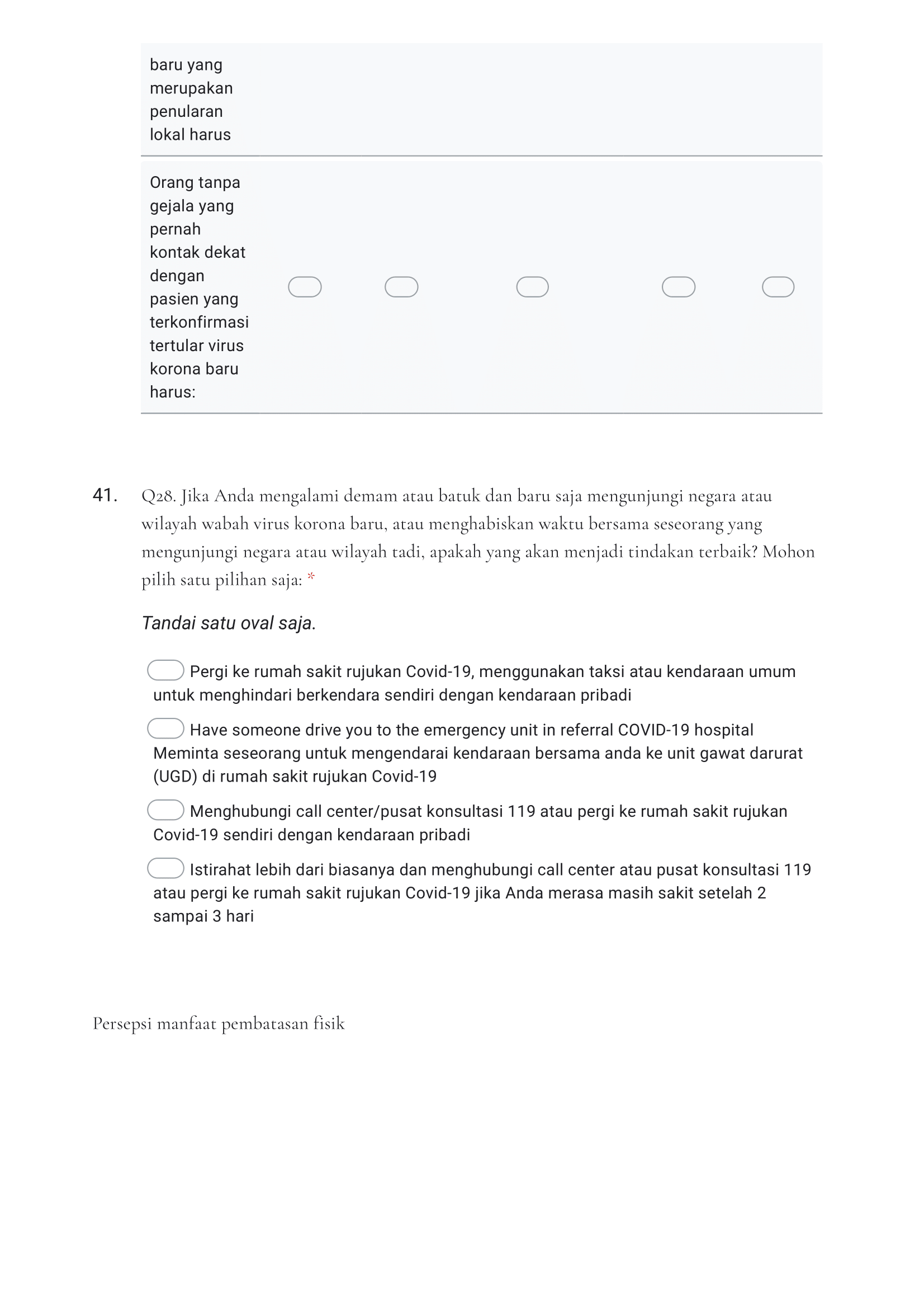

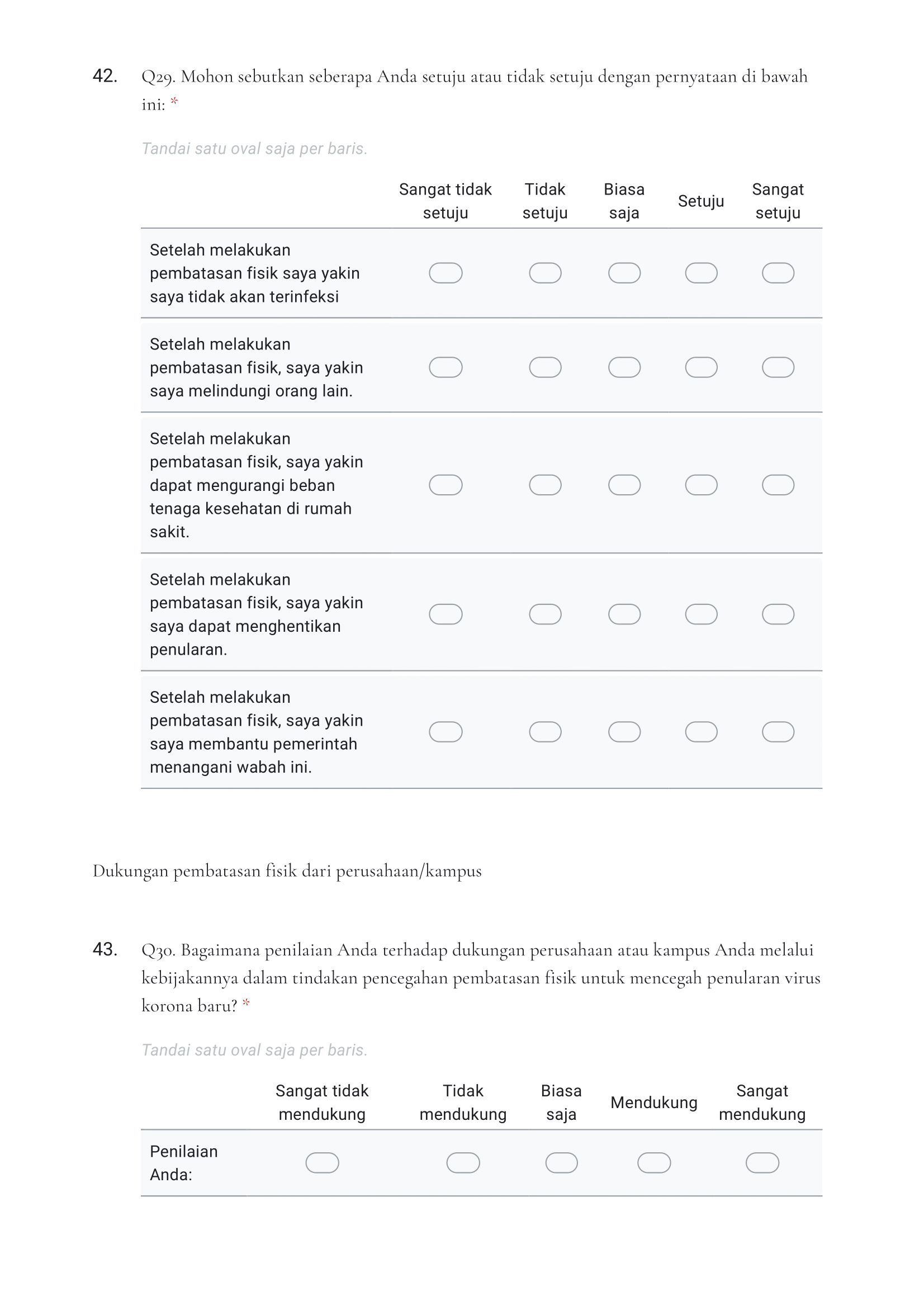

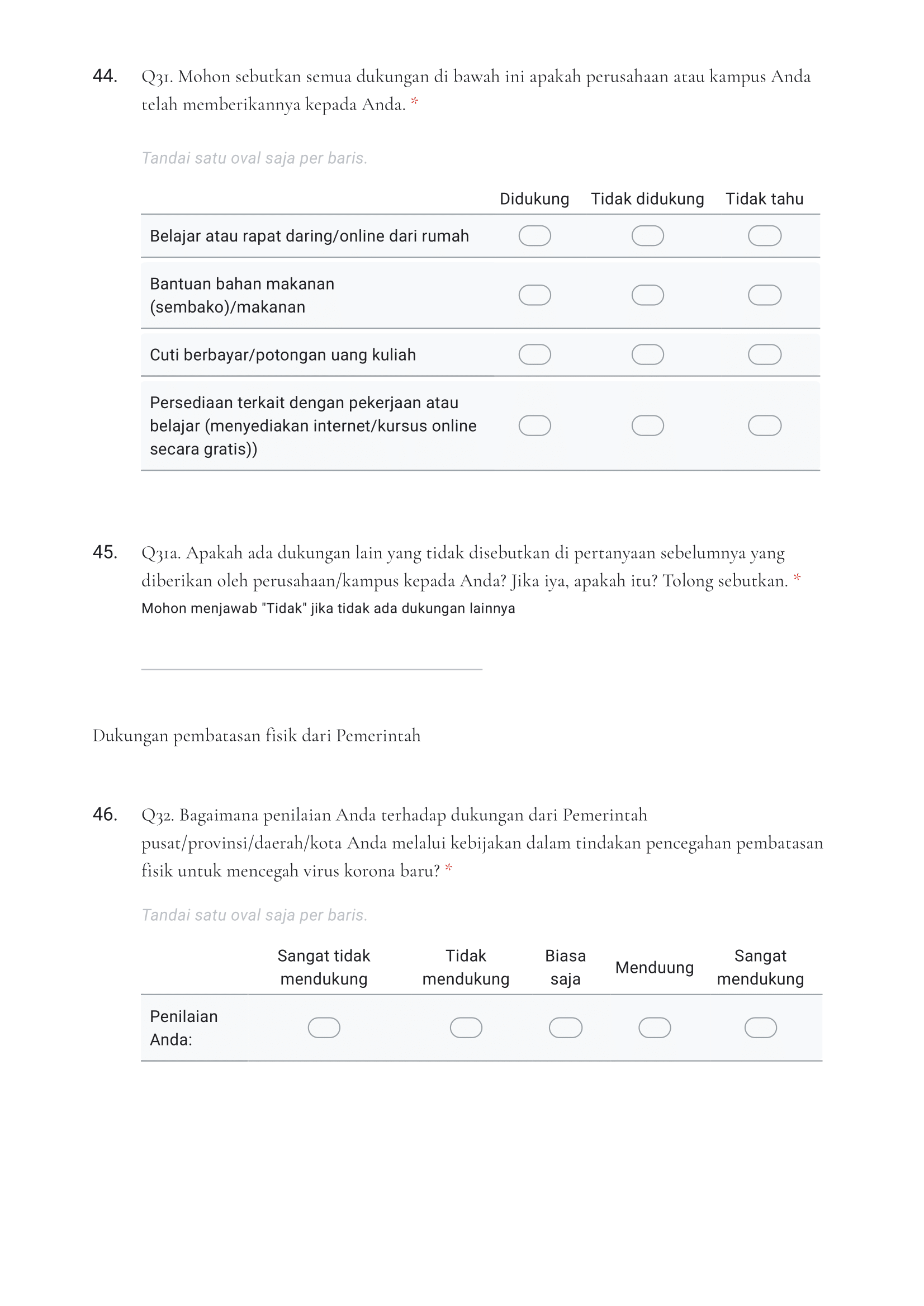

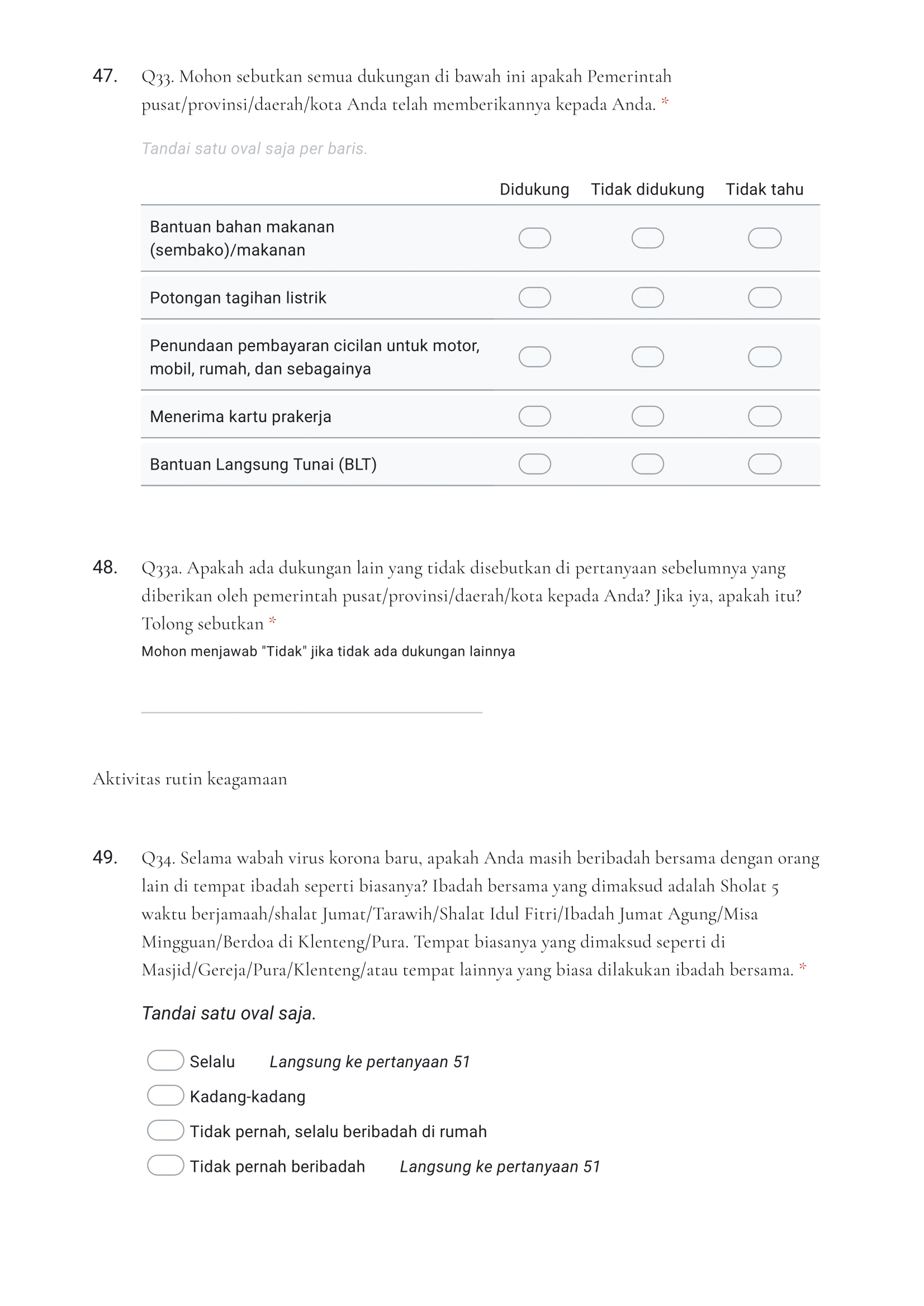

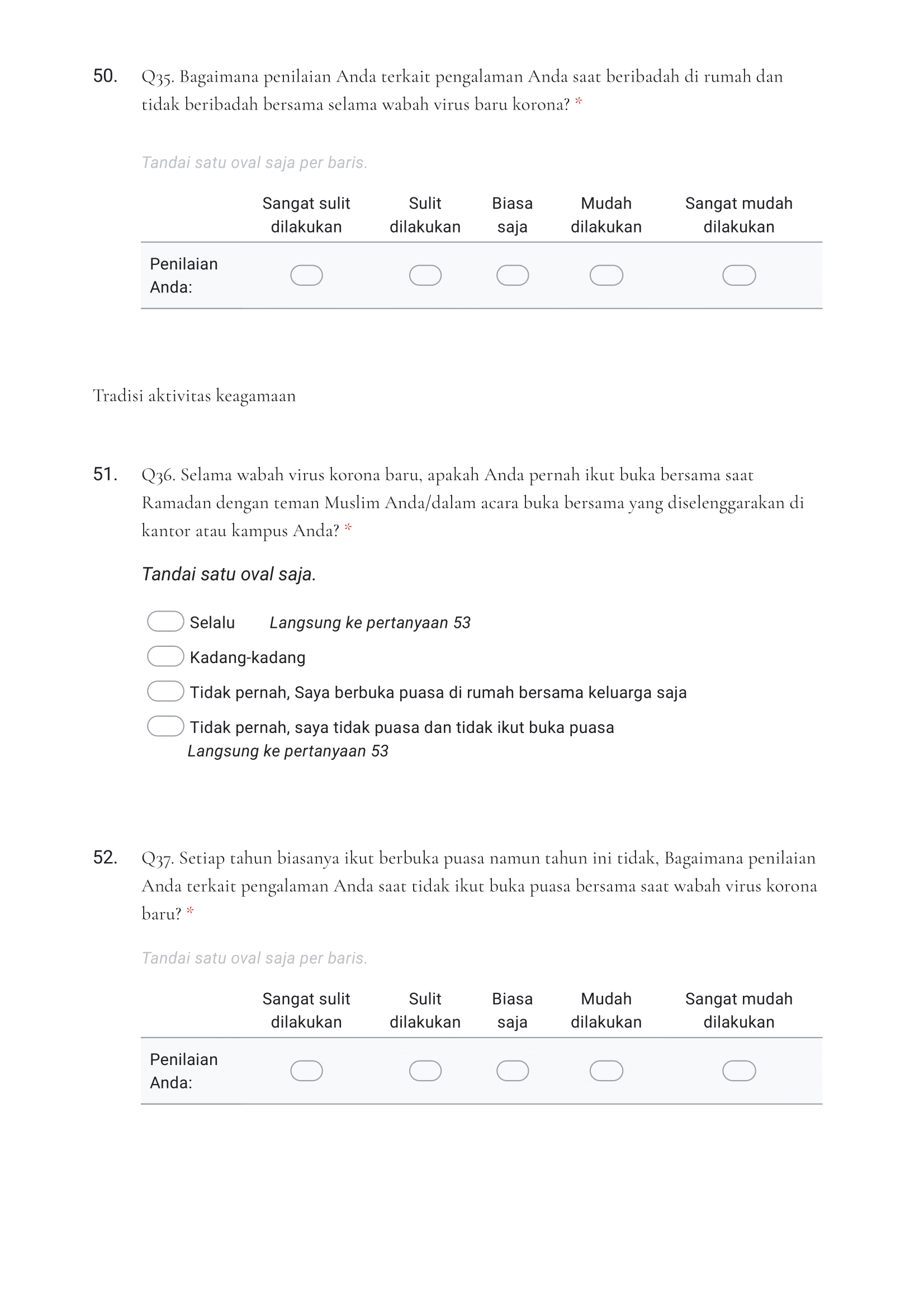

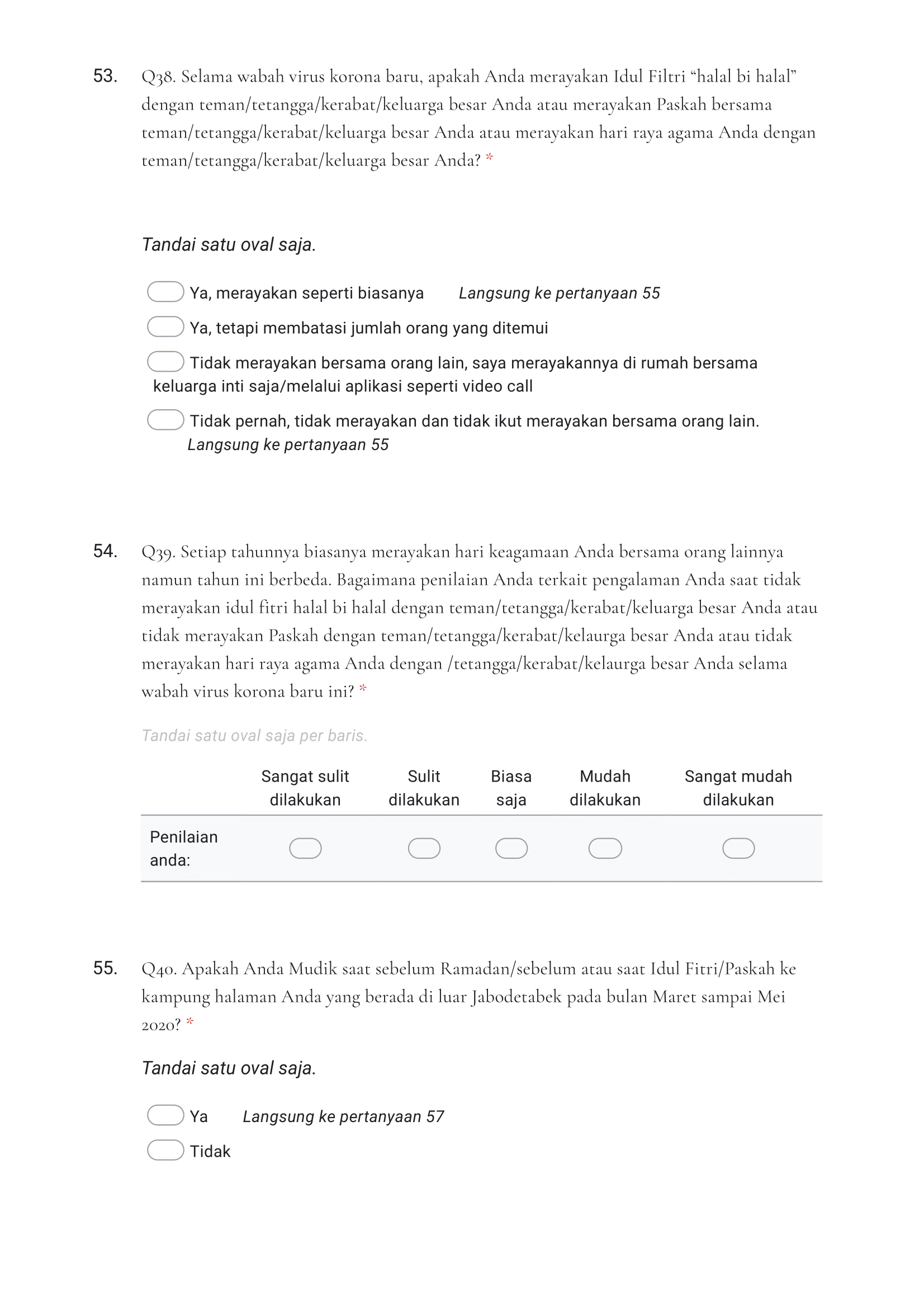

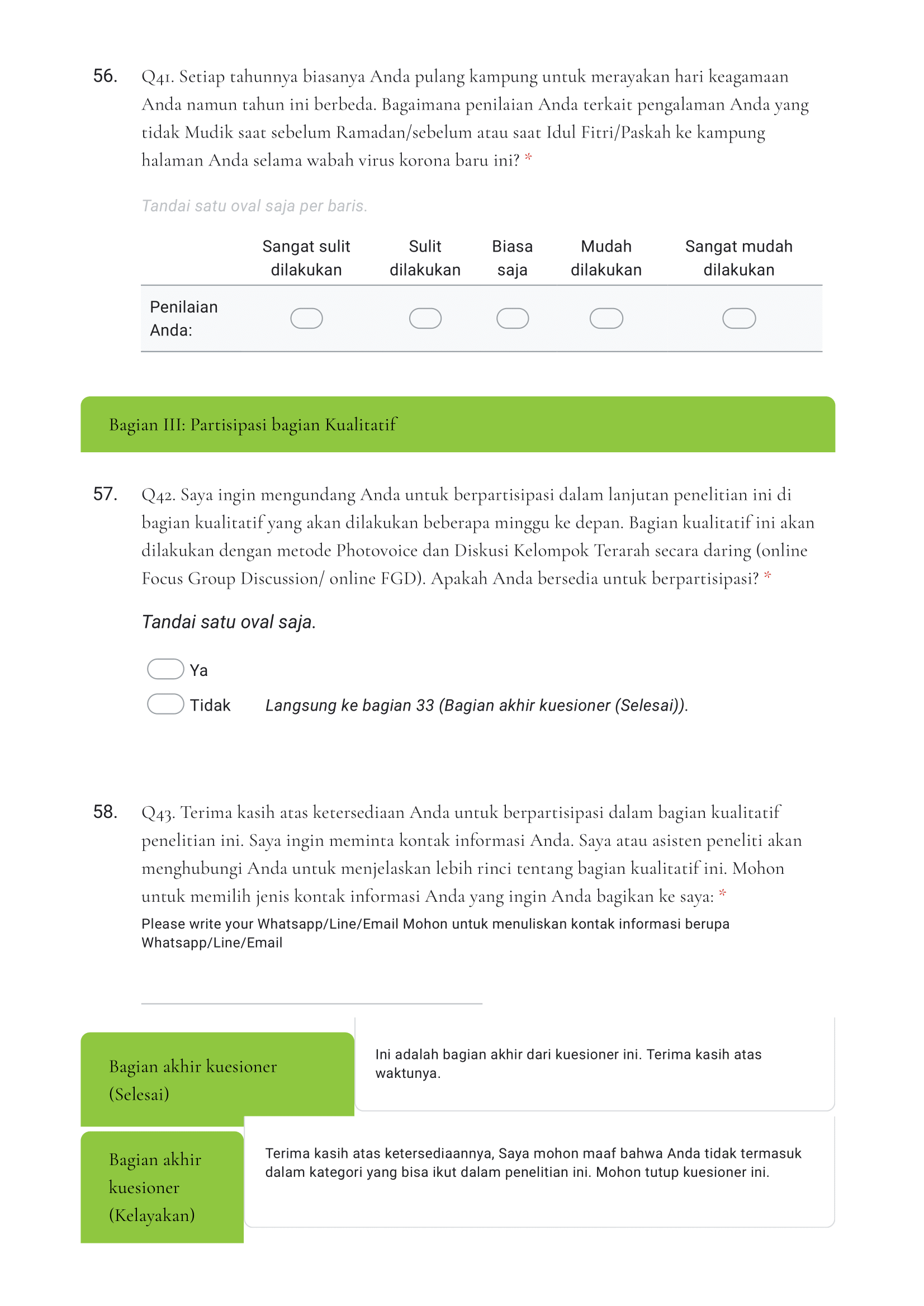

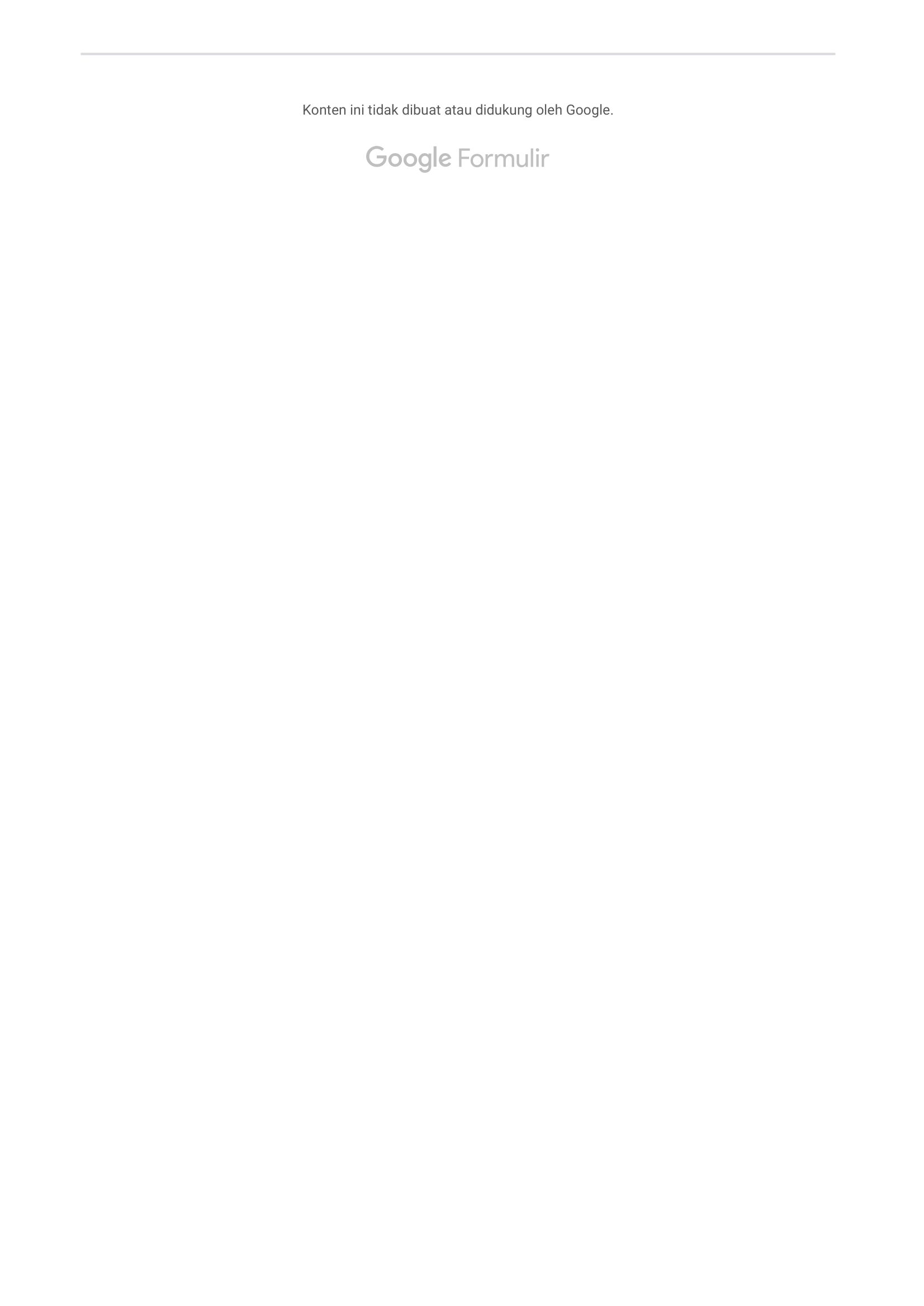

Supplement: S2 Questionnaire — (DOCX) [file pgph.0000035.s006.docx]
