# Supplementary material for: Factors influencing physical distancing compliance among young adults during COVID-19 pandemic in Indonesia: A photovoice mixed methods study
Source: PLOS Glob Public Health. 2022 Jan 13;2(1):e0000035. doi: 10.1371/journal.pgph.0000035 (PMC10021510; doi:10.1371/journal.pgph.0000035)
Supplement: S1 Interview guide — (DOCX) [file pgph.0000035.s007.docx]

**S1 Interview guide. Photovoice interview guide (English)**

Photovoice Guideline

**Coronavirus disease (COVID-19) pandemic: Barriers and facilitators to physical distancing among young adults in the Jakarta Metropolitan area, Indonesia.**

For Participant

This guideline has three parts: Initial Meeting, Online group discussion, and Photovoice ethics.

**Initial Meeting**

In this initial meeting, you will get the brief explanation of photovoice methods, brief ethic of photography explanation, how to take a photo in photovoice, phase of photovoice methods, and informed consent form.

Photovoice is a qualitative method that provides an opportunity to portray and share experience visually that may be difficult to express with words alone.

During taking photo, we have to consider the ethic of photography including privacy. If we want to take a photo that include someone’s face, we need to ask the permission to that person. Your safety is my main concern. Please consider your safety first when you want to take a photo. Photovoice ethic will be provided in the end of this guideline.

For photovoice, you will answer the question from a study by telling a story of your photo. In order to do that, you have to read the question first. You may take as many photos as you can, but you have to choose one up to three photos when you share in the next online group discussion. Since this study can’t provide you a camera to take a photo, you can use a smartphone/digital camera/other kind of cameras that can provide an electronic file.

The phase of photovoice methods are follows:

1. Joining the initial meeting (date)
2. Reading the information sheet and consent form in <insert survey site> (a range of week)
3. Downloading and reading a photovoice guideline
4. Taking photograph session (a range of week)
5. Confirmation after finish taking photograph (during taking photograph session or one to three days after taking photograph session)
6. Self-selection one up to three photos and write a caption and send it to researcher/research assistant
7. Giving an availability date of online group discussion (a range of week)
8. Joining the online group discussion (date)
9. Receiving an incentive of a 100,000-indonesian rupiah gift card

The goal of this study is to **identify barriers and facilitators of physical distance compliance among young adults in the Jakarta Metropolitan area.** There are three main physical distancing measures that are included in this study: avoiding use public transportation/rush hours, avoiding physical contact (handshake, hug, maintaining one-metre distance between person), and avoiding meet in person with anyone (stay at home/study or work from home/avoiding mass gathering, avoiding visit friend/family in other houses).

The theme question of this photovoice study:

**“Based on your experience during COVID-19 pandemic in Jakarta Metropolitan area, what are barriers/facilitators of physical distancing measure?”**

Read information sheet and consent form in this link: [http://survey-covid19.herokuapp.com/](about:blank)

**Online photovoice group discussion**

Online photovoice group discussion is the last part of this photovoice method. After finish taking photograph, you will join online discussion with a group of other participants in a chatroom. In this discussion, you will express your opinion by story-telling the photograph that have you taken in the previous stage.

Here is the flow of the online photovoice group discussion:

1. Researcher/research assistant will invite you to the chatroom by an invitation link through email/WhatsApp/Line.
2. You have to prepare an account of Zoom or Google Hangout and set to anonymous ID. Researcher and research assistant will help you to set anonymous ID prior the discussion.
3. There will be a moderator which is a researcher or research assistant in the discussion. Once you join the chatroom, you can only see moderator’s face and moderator’s screen. You can only see the other participants’ anonymous ID.
4. Moderator will start to introduce himself and ask the participants, including you, to introduce your nickname and where you are from. Then moderator explain about guideline including the rule of discussions.
5. After explaining the guideline, you will see the photographs slideshow in moderator’s screen. Those photographs are the self-selected and taken photographs by participants including you and you also will be asked to remember the photographs’ code in each photograph that showed in the slideshow because you have to mention when you express your opinion in the discussion.
6. Moderator open the discussion by asking the questions to all participants. You also can add your opinion to another participant’s answer. You have to mention your photographs’ ID when you answer the question. Moderator will show the photograph while someone’s answer mentions its ID in the chatroom through moderator’s screen.
7. Moderator will warp-up the discussion and share a link in chatroom. Then, moderator will ask you to click that link to choose one of 100,000-indonesian rupiah gift card as an incentive for joining this photovoice session.
8. Moderator end the discussion by asking participants to leave the chatroom.

If you have further questions about this study, please do not hesitate to contact us:

Ahmad Junaedi (Researcher)

Tel/Whatsapp: +62-812-9026-8627

E-mail: [ajunaedi@m.u-tokyo.ac.jp](about:blank)

Line: @junweasley

Fauzan Rachmatullah (Research Assistant)

Tel/Whatsapp: +62-812-2169-9625

E-mail: [fauzan.rachmatullah23@gmail.com](about:blank)

Line: @fauzanmadkip

**Photovoice ethics**

Photovoice is, by design, intended to include participants in participatory inquiry. They become documentary photographers at their site; their objective is to take pictures of activities, events, symbols, and people (photo subjects) that best respond to the framing (trigger) questions. Potential risks on participants’ safety to photovoice photographers from putting themselves in dangerous settings or situations and photo subject are our concern.

When you want to take a photograph, please remind yourself of these key concerns:

- ‘Shooting smart’ – maintaining your personal safety – is of highest priority. No photo is worth personal danger.
- Take your photos in public spaces (from which participants can photograph without being seen as trespassing) versus private property.
- As a documentary photographer, you must respect the privacy of others. If you want to take a photo of someone, please ask his or her permission before taking it.

Adapted from: Shumba TW, Moodley I. Part 2: The feasibility of utilising photovoice method and the World Health Organization Quality of Life instrument in evaluating the Community Based Rehabilitation programme in Namibia: A pilot study. Afr. J. Disabil. 2018;7(0):a419. doi:10.4102/ajod.v7i0.419

For Researcher/Research Assistant (Interview guide for discussion)

Photovoice online group discussion

Good morning/afternoon/evening.

Thanks for taking the time to join me and to talk about physical distancing during Covid-19 pandemic. My name is **Ahmad Junaedi, a master student at school of International Health, The University of Tokyo/ Fauzan Rachmatullah, research assistant of this research**. The goal of this study is to **identify barriers and facilitators of physical distance compliance among young adults in the Jakarta Metropolitan area**. This is the last phase of photovoice phases. In this discussion today I just want to talk about your experiences at physical distancing, by you using a photograph that you have taken before.

I don’t have physical distancing experience in Jakarta Metropolitan area and am just collecting the information, so I hope that you will feel comfortable to share with me what you really thought about your physical distancing experience. Please don’t feel shy, I want to hear from all of you about your experience. You are the experts because you have been done physical distancing for about two/three months and I am here to learn from you. There are no right or wrong answers, I simply want to hear your thoughts. I have some questions for you but also feel free to add other things you feel are important as we go along.

I will record and save the recording of our discussion in the end of the discussion. Our discussion will stay confidential and only the research team will listen to the recording and read to transcript. Is it OK with everyone?

During our discussion please let everyone share their views, but only one person should answer at a time. Just join in when you have something to say, we will not be going around the group for every question. Remember we want to hear all your views. It’s OK to disagree with others if you have a different opinion but please also respect other people’s views. Also, everything that you hear today should be confidential and not shared with people who are outside the group. This discussion will last about one hour. Are there any questions before we start?

**Let’s start by introducing ourselves**

1. Let’s each share our nicknames and where you are from.

Compliance, Barriers, and Facilitators of Physical Distancing Measure

**There are three main measures: avoiding use public transportation/rush hours, avoiding physical contact (handshake, hug, maintaining one-metre distance between person), and avoiding meet in person with anyone (stay at home/study or work from home/avoiding mass gathering, avoiding visit friend/family in other houses).**

1. Which are physical distancing measures that you find difficult/convenient to be complied?
2. What makes you think those physical distancing measures are difficult/convenient? Please share your answer by showing the taken photography.
3. Based on your photographs, could you answer these following questions:

What do you See here?

What's really Happening here?

How does this relate to Our lives?

Why does this problem, concern, or strength Exist?

What can we Do about it?

1. Are there any photographs that you might have wanted to take but you did not? If yes, can you tell me more about that?

**Are there any other things about barriers and facilitators to physical distancing that you would like to share before we finish?**

That concludes our focus group. Thank you so much for joining and sharing your thoughts and opinions with me. I would like for you to click this a google form link to choose one of 100,000-indonesian rupiah gift card as an incentive for joining this study.
