# Supplementary material for: Factors influencing physical distancing compliance among young adults during COVID-19 pandemic in Indonesia: A photovoice mixed methods study
Source: PLOS Glob Public Health. 2022 Jan 13;2(1):e0000035. doi: 10.1371/journal.pgph.0000035 (PMC10021510; doi:10.1371/journal.pgph.0000035)
Supplement: S2 Interview guide — (DOCX) [file pgph.0000035.s008.docx]

**S2 Interview guide. Photovoice interview guide (Indonesian)**

Panduan Photovoice

**Pandemik penyakit virus korona (Covid-19): Tantangan dan faktor pendukung dalam pembatasan fisik di kalangan dewasa muda di Jabodetabek, Indonesia**

Untuk Peserta

Panduan ini memiliki tiga bagian: Pertemuan Awal, Diskusi Kelompok daring, dan Etika *Photovoice*.

**Pertemuan Awalan**

Dalam pertemuan awalan ini, Anda akan menerima penjelasan singkat tentang metode *photovoice*, etika fotografi, bagaimana cara mengambil foto dalam *photovoice*, fase-fase dalam metode *photovoice*, dan lembar persetujuan penelitian (*informed consent*).

*Photovoice* adalah metode kualitatif yang menyediakan kesempatan untuk menggambarkan dan membagikan pengalaman melalui foto yang mungkin sulit disampaikan hanya melalui kata-kata saja.

Dalam pengambilan foto, kita harus memikirkan etika fotografi, termasuk privasi seseorang. Jika kita ingin mengambil foto yang mengandung wajah seseorang, kita harus meminta izin terlebih dahulu kepada orang tersebut. Keselamatan Anda adalah prioritas saya. Mohon untuk mengutamakan keselamatan Anda, termasuk mematuhi protokol kesehatan terlampir, ketika ingin mengambil foto. Etika *photovoice* tersedia di bagian akhir dari panduan ini.

Dalam *photovoice*, Anda akan menjawab pertanyaan yang diberikan dengan cara menceritakan jawaban Anda melalui foto. Untuk melakukan hal tersebut, Anda harus membaca pertanyaan yang diberikan terlebih dahulu. Anda dapat mengambil foto sebanyak mungkin untuk menjawab pertanyaan yang diberikan, tetapi Anda harus memilih satu sampai tiga foto yang akan Anda bagikan dalam diskusi kelompok yang akan dilakukan secara daring nantinya. Dikarenakan penelitian ini tidak dapat menyediakan kamera untuk pengambilan foto, Anda dapat menggunakan kamera dari ponsel Anda atau kamera digital atau jenis kamera lainnya yang dapat menyediakan file elektronik.

Fase-fase dalam metode *photovoice* adalah sebagai berikut:

1. Mengikuti pertemuan awalan pada tanggal (masukkan tanggal)
2. Membaca lembar informasi dan lembar persetujuan penelitian (*informed consent*) dalam situs [http://survey-covid19.herokuapp.com](about:blank) mulai dari tanggal … sampai tanggal … (masukkan tanggal rentang waktu)
3. Mengunduh dan membaca panduan *photovoice*
4. Sesi pengambilan fotografi yang dilakukan dari tanggal … sampai tanggal …. (masukkan tanggal rentang waktu)
5. Mengkonfirmasi kepada peneliti atau asisten peneliti setelah selesai pengambilan fotografi. Konfirmasi ini dapat dilakukan baik saat masih dalam sesi pengambilan fotografi atau satu sampai tiga hari setelah sesi pengambilan fotografi selesai
6. Pemilihan foto secara mandiri satu sampai tiga foto yang terbaik, yang dapat menjawab dengan baik pertanyaan penelitian. Menuliskan keterangan masing-masing foto dan mengirimkannya kepada peneliti atau asisten peneliti
7. Memberikan ketersediaan waktunya untuk mengikuti diskusi kelompok secara daring pada tanggal yang akan diberitahukan dikemudian hari
8. Menghadiri diskusi kelompok daring pada tanggal yang nanti akan diberitahukan
9. Menerima insentif sebesar Rp100.000 dalam bentuk *voucher*

Tujuan dari penelitian ini adalah untuk mengidentifikasi tantangan dan faktor pendukung dalam kepatuhan pembatasan fisik di kalangan dewasa muda di Jabodebatek**.** Terdapat tiga tindakan utama dalam pembatasan fisik yang termasuk dalam penelitian ini: menghindari penggunaan kendaraan umum/jam sibuk, menghindari kontak secara fisik (salaman, berpelukan, jaga jarak 1 meter) dan menghindari untuk bertemu dengan siapapun (tetap tinggal di rumah/belajar atau bekerja dari rumah/menghindari kerumunan/menghindari mengunjungi teman/keluarga yang tidak tinggal dalam satu rumah).

Tema pertanyaan dalam metode *photovoice* ini yaitu:

**Berdasarkan pengalaman Anda selama wabah Covid-19 di Jabodetabek, apakah tantangan atau faktor pendukung Anda untuk tindakan pembatasan fisik?”**

Lembar informasi dan lembar persetujuan penelitian (*informed consent*) dapat dibaca dalam situs ini: [http://survey-covid19.herokuapp.com](about:blank)

**Diskusi kelompok *photovoice* daring**

Diskusi kelompok *photovoice* daring adalah bagian terakhir dalam metode *photovoice* ini. Setelah sesi pengambilan fotografi selesai, Anda akan bergabung dalam diskusi kelompok dengan peserta lainnya melalui *chatroom*. Dalam diskusi ini, Anda akan menyampaikan opini atau jawaban Anda dengan menceritakannya melalui foto yang sudah Anda ambil dalam tahapan sebelumnya.

Berikut ini adalah alur dari diskusi kelompok *photovoice* daring:

1. Peneliti/asisten peneliti akan mengundang Anda ke dalam *chatroom* melalui tautan undangan yang akan dikirimkan melalui email/WhatsApp/Line.
2. Anda harus menyiapkan akun Zoom atau Google Meeting (Anda diminta untuk membuatnya jika belum memiliki akun tersebut) dan mengaturnya menjadi identitas anonim. Peneliti dan asisten peneliti akan membantu Anda untuk mengatur identitas anonim tersebut sebelum diskusi.
3. Peneliti atau asisten peneliti akan bertindak sebagai moderator pada diskusi ini. Setelah Anda bergabung dalam *chatroom*, Anda hanya akan dapat melihat wajah moderator dan layar komputer moderator. Anda hanya dapat melihat identitas anonim peserta lainnya.
4. Moderator akan memperkenalkan dirinya dan meminta peserta, termasuk Anda, untuk memperkenalkan nama panggilan dan asal tempat tinggal Anda. Lalu moderator akan menjelaskan mengenai panduan dalam diskusi termasuk aturan diskusi.
5. Setelah panduan dijelaskan, Anda akan melihat foto-foto yang ditayangkan dari layar komputer. Foto-foto tersebut adalah foto-foto pilihan peserta sendiri yang diambil oleh semua peserta termasuk Anda. Dalam tayangan foto tersebut, Anda akan melihat juga kode dari masing-masing foto dan Anda diminta untuk menuliskan kode foto Anda tersebut. Anda harus mengingat kode tersebut karena Anda harus menyebutkannya ketika Anda menyampaikan opini atau jawaban Anda dalam diskusi.
6. Moderator membuka diskusi dengan menyebutkan pertanyaan yang ditujukan ke semua peserta. Anda juga dapat menambahkan opini Anda terhadap jawaban peserta lainnya. Anda harus menyebutkan kode foto Anda saat menjawab pertanyaan atau menyampaikan opini Anda. Moderator akan menayangkan foto pada layarnya ketika kode foto tersebut disebut dalam jawaban atau opini yang disampaikan oleh peserta.
7. Moderator akan menutup diskusi dan membagikan sebuah tautan di *chatroom*. Selanjutnya, moderator akan meminta Anda untuk klik untuk memilih salah satu voucher senilai Rp100,000 sebagai insentif mengikuti sesi *photovoice*.
8. Moderator menyudahi diskusi dengan meminta semua peserta meninggalkan *chatroom*.

Seperti yang sudah dijelaskan sebelumnya, dalam penelitian ini Anda akan membutuhkan perangkat elektronik seperti kamera digital atau jenis kamera lainnya atau ponsel untuk pengambilan foto dan perangkat eletronik untuk menghadiri diskusi daring seperti ponsel, komputer, tablet, laptop, dan sejenisnya. Mohon untuk mematuhi protokol kesehatan terlampir.

Jika Anda memiliki pertanyaan lebih lanjut mengenai penelitian ini, mohon jangan sungkan untuk menghubungi kami:

Ahmad Junaedi (Peneliti)

Tel/Whatsapp: +62-812-9026-8627

E-mail: [ajunaedi@m.u-tokyo.ac.jp](about:blank)

Line: @junweasley

Fauzan Rachmatullah (Asisten Peneliti)

Tel/Whatsapp: +62-812-2169-9625

E-mail: [fauzan.rachmatullah23@gmail.com](about:blank)

Line: @fauzanmadkip

**Etika *Photovoice***

*Photovoice*, berdasarkan desain tujuannya, dimaksudkan untuk melibatkan partisipasi dari peserta dalam menjawab sebuah pertanyaan. Para peserta akan menjadi fotografer dokumenter di wilayanya masing-masing; tujuan mereka adalah untuk mengambil foto tentang kegiatan, acara, simbol, dan orang (subyek dari foto) yang merupakan respon terbaik untuk menggambarkan sesuatu keadaan berdasarkan pertanyaan yang diberikan. Keselamatan peserta sebagai fotografer *photovoice* menjadi perhatian khusus bagi kami terhadap adanya potensi risiko yang mungkin muncul saat peserta menempatkan dirinya pada kondisi atau situasi yang berbahaya dalam proses pengambilan foto.

Ketika Anda akan mengambil fotografi, mohon untuk mengingatkan kembali kepada diri Anda sendiri beberapa hal berikut:

- ‘Pengambilan foto yang cerdas’ – menjaga keamanan Anda sendiri – adalah prioritas tertinggi. Tidak ada foto yang setara dengan keselamatan Anda dalam bahaya.
- Ambillah foto di tempat umum (yang mana peserta dapat mengambil foto tanpa terlihat sebagai pelanggaran) daripada di tempat pribadi milik orang lain.
- Mohon untuk mematuhi protokol kesehatan terlampir.
- Sebagai fotografer dokumenter, Anda harus menghormati privasi orang lain. Jika Anda ingin mengambil foto seseorang, mohon meminta izin mereka terlebih dahulu.

Diadaptasi dari:

Shumba TW, Moodley I. Part 2: The feasibility of utilising photovoice method and the World Health Organization Quality of Life instrument in evaluating the Community Based Rehabilitation programme in Namibia: A pilot study. Afr. J. Disabil. 2018;7(0):a419. doi:10.4102/ajod.v7i0.419

Untuk peneliti/asisten peneliti (Panduan wawancara dalam diskusi)

Diskusi kelompok *photovoice* daring

Selamat pagi/siang/sore/malam.

Terima kasih atas waktunya untuk bergabung bersama saya untuk berbagi pengalaman mengenai pembatasan fisik selama Covid-19 pandemik. Nama saya adalah **Ahmad Junaedi, mahasiswa S2 di school of International Health, The University of Tokyo/ Fauzan Rachmatullah, asisten peneliti dalam penelitian ini**. Tujuan dari penelitian ini adalah **untuk mengidentifikasi tantangan dan faktor pendukung dalam kepatuhan pembatasan fisik di kalangan dewasa muda di Jabodetabek**. Ini merupakan fase terakhir dalam metode *photovoice*. Dalam diskusi hari ini, Saya ingin mendengarkan cerita Anda tentang pengalaman Anda selama pembatasan fisik dengan menggunakan foto yang sudah Anda ambil sebelumnya.

Saya tidak memiliki pengalaman dalam pembatasan fisik di Jabodetabek dan saya hanya mengumpulkan informasi mengenai hal itu, oleh karenanya Saya berharap Anda merasa nyaman untuk berbagi cerita kepada saya mengenai apa yang ada dalam pikiran Anda tentang pengalaman pembatasan fisik. Mohon untuk tidak merasa malu, Saya ingin mendengar dari masing-masing orang yang ada di sini mengenai pengalamannya. Anda semua adalah ahlinya karena Anda telah mengalami pembatasan fisik sekitar dua sampai tiga bulan dan saya di sini ingin belajar dari Anda semua. Tidak ada jawaban yang benar atau yang salah, saya hanya ingin mendengar apa yang ada dalam pemikiran Anda. Saya memiliki beberapa pertanyaan untuk Anda semua nanti.

Diskusi ini akan direkam dan pada akhir diskusi saya akan menyimpan rekaman diskusi kita kali ini. Diskusi kita ini akan tetap bersifat rahasia. Hanya tim peneliti yang akan mendengarkan rekaman dan membaca transkripnya. Apakah ada yang keberatan jika diskusi ini direkam?

Selama diskusi, setiap orang dipersilakan untuk membagikan pemikirannya, tetapi hanya satu orang yang menjawab dalam satu waktu. Dipersilakan untuk langsung menjawab atau menambahkan jawaban atau opini jika ada sesuatu yang ingin dikatakan, Saya tidak akan bertanya ke setiap orang satu per satu untuk setiap pertanyaan. Mohon diingat bahwa saya ingin mendengar pemikiran Anda semua. Sangat tidak masalah jika tidak setuju dan berbeda pendapat dengan peserta lain namun mohon juga untuk tetap menghormati pemikiran orang lain. Semua yang Anda dengar pada hari ini harus dirahasiakan dan tidak dibagikan atau diceritakan kepada siapapun diluar kelompok diskusi ini. Diskusi akan berlangsung sekitar 1 jam. Apakah ada yang ingin ditanyakan sebelum kita mulai?

**Mari perkenalkan diri**

1. Mari perkenalkan nama panggilan dan asal tempat tinggal. Bisa dimulai dari *menyebutkan identitas anonim salah satu peserta*

Kepatuhan, Tantangan, dan Faktor pendukung dalam tindakan pembatasan fisik

**Ada tiga tindakan utama dalam pembatasan fisik yaitu yang pertama menghindari penggunaan kendaraan umum/jam sibuk, yang kedua menghindari kontak fisik seperti bersalaman, berpelukan, jaga jarak satu meter, dan yang ketiga yaitu menghindari untuk bertemu dengan siapapun seperti tetap di rumah saja, kerja atau belajar dari ruman, menghindari kerumunan, dan menghindari untuk bertemu dengan teman atau keluarga yang tidak tinggal dalam satu rumah.**

1. Dari tindakan-tindakan pembatasan fisik tersebut, menurut Anda mana yang sulit dan mana yang mudah untuk dipatuhi?
2. Apa yang membuat Anda berpikir seperti itu (bahwa tindakan tersebut sulit atau mudah untuk dipatuhi? Mohon untuk menjawab pertanyaan ini dengan menceritakan pengalaman atau jawaban Anda melalui foto yang Anda sudah ambil. Anda dapat menyebutkan kodenya agar dapat ditayangkan dalam layar komputer moderator sehingga semua peserta dapat melihatnya.
3. Berdasarkan foto Anda, mohon jawab beberapa pertanyaan ini:

Apa yang anda lihat di sini (foto Anda)?

Apa yang sebenarnya terjadi di sini (foto Anda)?

Bagaimana (foto Anda) ini berkaitan dengan kehidupan kita?

Mengapa masalah, perhatian, atau kekuatan (dalam foto) ini ada/terjadi?

Apa yang bisa kita lakukan mengenai hal tersebut?

1. Apakah ada foto yang sebenarnya Anda ingin sekali ambil tetapi Anda tidak jadi ambil? Jika ada, dapatkah Anda menceritakan lebih jauh tentang hal tersbut?

**Apakah ada hal-hal lain tentang tantangan dan faktor pendukung dalam pembatasan fisik yang Anda ingin bagikan ceritanya sebelum kita sudahi diskusinya?**

Inilah akhir dari diskusi kelompok *photovoice* daring. Terima kasih banyak telah bergabung dan berbagi pengalaman dan opini Anda dengan Saya. Saya meminta Anda untuk klik tautan ini [http://survey-covid19.herokuapp.com](about:blank) untuk memilih salah satu *voucher* senilai Rp100.000 sebagai insentif telah terlibat dalam penelitian ini.
