# Supplementary material for: Factors influencing physical distancing compliance among young adults during COVID-19 pandemic in Indonesia: A photovoice mixed methods study
Source: PLOS Glob Public Health. 2022 Jan 13;2(1):e0000035. doi: 10.1371/journal.pgph.0000035 (PMC10021510; doi:10.1371/journal.pgph.0000035)
Supplement: S3 Interview guide — (DOCX) [file pgph.0000035.s009.docx]

**S3 Interview guide. Online FGDs interview guide (English)**

Online Focus Group Discussion Guide

**Coronavirus disease (COVID-19) pandemic: Barriers and facilitators to physical distancing among young adults in the Jakarta Metropolitan area, Indonesia.**

For Participant

**Initial Interview**

In this initial interview, you will get the brief explanation of online focus group discussion (Online FGD) and the flow of Online FGD. You also have to give availability date and time for joining Online FGD.

Focus group discussion is a method to get broaden understanding of research topic. Similar to traditional focus group discussion, Online FGD also can provide different perspective and opinion from interaction of participants in a moderated discussion.

The goal of this study is to **identify barriers and facilitators of physical distance compliance among young adults in the Jakarta Metropolitan area.** During Online FGD, please do not feel shy, this research wants to hear from you about your experience. You are the experts because you have been done physical distancing for about two/three months and researcher/research assistant is here to learn from you. There are no right or wrong answers simply researcher/research assistant want to hear your thoughts and suggestions.

The flow of Online FGD:

1. Researcher/research assistant will invite you to the chatroom by an invitation link through email/WhatsApp/Line.
2. You have to prepare an account of Zoom or Google Hangout and set to anonymous ID. Researcher and research assistant will help you to set anonymous ID prior the discussion.
3. There will be a moderator which is a researcher or research assistant in the discussion. Once you join the chatroom, you can only see moderator’s face and moderator’s screen. You can only see the other participants’ anonymous ID.
4. Moderator will start to introduce himself and ask the participants, including you, to introduce your nickname and where you are from. Then moderator will give a link that contains information sheet and informed consent. You will receive electronic informed consent after you sign it. After reading the information sheet and signing the informed consent, you have to go back to Online FGD’s chatroom.
5. Moderator will explain about guideline including the rule of discussions after all participants read the information sheet and sign the informed consent.
6. Moderator will open the discussion by asking the questions. You also can add your opinion to another participant’s answer.
7. Moderator will warp-up the discussion and share a link in chatroom. Then, moderator will ask you to click that link to choose one of 100,000-indonesian rupiah gift card as an incentive for joining this Online FGD session.
8. Moderator end the discussion by asking participants to leave the chatroom.

During the discussion please let everyone share their views, but only one person should answer at a time. Just join in when you have something to say, moderator will not be going around the group for every question. Remember moderator want to hear all your views. It’s OK to disagree with others if you have a different opinion but please also respect other people’s views. Also, everything that you hear today should be confidential and not shared with people who are outside the group. This chat history of our discussion in the end of the discussion. The chat history will stay confidential and only the research team will read to the chat history. This discussion will last about one hour.

Please do not forget to give your availability date and time for Online FGD to researcher/research assistant during initial interview. You will get notification about your Online FGD schedule from researcher or research assistant through email/WhatsApp/Line.

If you have further questions about this study, please do not hesitate to contact us:

Ahmad Junaedi (Researcher)

Tel/Whatsapp: +62-812-9026-8627

E-mail: [ajunaedi@m.u-tokyo.ac.jp](mailto:ajunaedi@m.u-tokyo.ac.jp)

Line: @junweasley

Fauzan Rachmatullah (Research Assistant)

Tel/Whatsapp: +62-812-2169-9625

E-mail: [fauzan.rachmatullah23@gmail.com](mailto:fauzan.rachmatullah23@gmail.com)

Line: @fauzanmadkip

For Research and Research Assistant (Interview guide for Online FGDs)

Online Focus Group Discussion

Good morning/afternoon/evening.

Thanks for taking the time to join me and to talk about physical distancing during Covid-19 pandemic. My name is **Ahmad Junaedi, a master student at school of International Health, The University of Tokyo**. The goal of this study is to **identify barriers and facilitators of physical distance compliance among young adults in the Jakarta Metropolitan area**. The best way to do this is to talk to people who experienced the physical distancing in Jakarta Metropolitan area, so I are holding these online discussion groups with some young adults this month. In our discussion today I just want to talk about your experiences at physical distancing, what are the difficulties did you have, and what are the enable factors did you had.

As a group, I am going to go over the informed consent form before we start our focus group to be sure that you understand why we are having this focus group and to be sure that you voluntarily want to participate. I provide a google form link <http://survey-covid19.herokuapp.com> that contains the informed sheet and consent question. So, please click the link and read the informed sheet. Then answer the consent question.

I don’t have physical distancing experience in Jakarta Metropolitan area and am just collecting the information, so I hope that you will feel comfortable to share with me what you really thought about your physical distancing experience. Please don’t feel shy, I want to hear from all of you about your experience. You are the experts because you have been done physical distancing for about two/three months and I am here to learn from you. There are no right or wrong answers we simply want to hear your thoughts and suggestions. I have some questions for you but also feel free to add other things you feel are important as we go along.

I will record and save the recording of our discussion in the end of the discussion. Our discussion will stay confidential and only the research team will listen to the recording and read to transcript. Is it OK with everyone?

During our discussion please let everyone share their views, but only one person should answer at a time. Just join in when you have something to say, we will not be going around the group for every question. Remember we want to hear all your views. It’s OK to disagree with others if you have a different opinion but please also respect other people’s views. Also, everything that you hear today should be confidential and not shared with people who are outside the group. This discussion will last about one hour. Are there any questions before we start?

**Let’s start by introducing ourselves**

1. Let’s each share our nicknames and where you are from.

Length of Physical Distancing

**First, I would like to know about how long you did physical distancing such as stay at home, avoiding mass gathering, and avoiding meet with anyone else in person**

1. How long did you do physical distancing? (probe: each physical distancing measure)
2. Did you find any problems? (probe: why?)

Compliance, Barriers, and Facilitators of Physical Distancing Measure

**Now let’s focus on physical distancing measure. There are three main measures: avoiding use public transportation/rush hours, avoiding physical contact (handshake, hug, maintaining one-metre distance between person), and avoiding meet in person with anyone (stay at home/study or work from home/avoiding mass gathering, avoiding visit friend/family in other houses)**

1. Have you complied all of three main physical distancing measures?
2. What are the factors that makes you complied? (probe: each physical distancing measure)
3. Which are physical distancing measures that you find difficult to be complied?
4. What makes you think those physical distancing measures, in question number 6, are difficult?
5. Which are physical distancing measures that you find convenient to be complied?
6. What makes you think those physical distancing measures, in question number 8, are convenient?

Tradition religious activities

**As we know during Covid-19 pandemic in Indonesia, all of you have to adjust to celebrate Indonesia tradition Ramadan activities including *Mudik*, *Buka Bersama*, *Ngabuburit*, *Tarawih* and *Halal bi halal*. Even though not all of you are Muslim, but sometimes you may join those activities with your Muslim friends or in your company/school event.**

1. Do you think avoiding those activities is difficult/convenient?
2. What makes you think avoiding those activities are difficult/convenient?

**Are there any other things about barriers and facilitators to physical distancing that you would like to share before we finish?**

That concludes our focus group. Thank you so much for joining and sharing your thoughts and opinions with me. I would like for you to click this a google form link to choose one of 100,000-indonesian rupiah gift card as an incentive for joining this study.
