# Supplementary material for: Factors influencing physical distancing compliance among young adults during COVID-19 pandemic in Indonesia: A photovoice mixed methods study
Source: PLOS Glob Public Health. 2022 Jan 13;2(1):e0000035. doi: 10.1371/journal.pgph.0000035 (PMC10021510; doi:10.1371/journal.pgph.0000035)
Supplement: S4 Interview guide — (DOCX) [file pgph.0000035.s010.docx]

**S4 Interview guide. Online FGDs interview guide (Indonesian)**

Panduan Diskusi Kelompok Terumpun (DKT) daring

**Pandemik penyakit virus korona: Tantangan dan faktor pendukung dalam pembatasan fisik di kalangan dewasa muda di Jabodetabek, Indonesia.**

Untuk Peserta

**Pertemuan Awalan**

Dalam pertemuan awalan ini, Anda akan mendapatkan informasi mengenai penjelasan singkat tentang Diskusi Kelompok Terumpun (DKT) daring dan alurnya. Anda juga harus memberikan tanggal dan waktu ketersediaan untuk mengikuti DKT daring.

Diskusi kelompok Terumpun adalah sebuah metode untuk mendapatkan pemahaman yang luas dari topik penelitian. Sama halnya dengan DKT pada umumnya, DKT daring ini juga dapat menghasilkan pandangan dan opini yang berbeda melalui interaksi antar peserta dalam diskusi yang dimoderasi.

**Tujuan dari penelitian ini adalah untuk mengidentifikasi tantangan dan faktor pendukung dalam kepatuhan pembatasan fisik di kalangan dewasa muda di Jabodetabek.** Selama DKT secara daring ini, jangan merasa malu untuk berbicara. Penelitian ini mengharapkan keterbukaan Anda mengenai pengalaman Anda saat pandemik. Anda adalah ahli dalam hal ini karena Anda telah melakukan pembatasan fisik selama dua hingga tiga bulan dan peneliti/asisten peneliti di sini ingin belajar mengenai hal tersebut dari Anda. Tidak ada jawaban yang benar atau salah dalam penelitian ini. Peneliti/asisten peneliti sekedar ingin mendengarkan pemikiran dan saran Anda.

Berikut adalah alur dari DKT daring:

1. Peneliti/asisten peneliti akan mengundang Anda ke dalam *chatroom* lewat undangan berupa tautan melalui email/WhatsApp/Line.
2. Anda harus menyiapkan akun Zoom atau Google Hangout (Anda diminta untuk membuatnya jika belum memiliki akun tersebut) dan mengaturnya menjadi identitas anonim. Peneliti dan asisten peneliti akan membantu Anda untuk mengatur identitas anonim tersebut sebelum diskusi.
3. Peneliti atau asisten peneliti akan bertindak sebagai moderator dalam diskusi ini. Setelah Anda bergabung dalam *chatroom*, Anda hanya akan dapat melihat wajah moderator dan layar komputer moderator. Anda hanya dapat melihat identitas anonim peserta lainnya.
4. Moderator akan memperkenalkan diri dan meminta peserta, termasuk Anda, untuk memperkenalkan nama panggilan dan asal tempat tinggal Anda. Lalu moderator akan memberikan sebuah tautan yang mengandung lembar informasi dan lembar persetujuan penelitian (*informed consent*). Anda akan diminta untuk membaca dokumen tersebut dan akan menerima lembar persetujuan penelitian (*informed consent*) dalam bentuk elektronik file setelah Anda menandatanganinya. Setelah membaca lembar informasi dan menandatangani lembar persetujuan penelitian (*informed consent*), Anda diminta untuk kembali ke *chatroom* tadi.
5. Moderator akan menjelaskan mengenai panduan DKT daring ini termasuk aturan dalam berdiskusi setelah semua peserta membaca lembar informasi dan menandatangani lembar persetujuan penelitian (*informed consent*).
6. Moderator akan membuka diskusi dengan memulai bertanya beberapa pertanyaan. Peserta dapat menjawab pertanyaan tersebut dengan cara memberikan jawaban via suara (berbicara). Anda juga dapat menambahkan opini Anda ke dalam jawaban atau opini peserta lainnya.
7. Moderator akan menyatakan diskusi sudah berakhir dan akan membarikan sebuah tautan dalam *chatroom*. Selanjutnya, moderator meminta Anda untuk klik tautan tersebut untuk memilih salah satu *voucher* senilai Rp100.000 sebagai insentif telah bergabung dalam sesi DKT secara daring ini.
8. Moderator menutup diskusi dengan meminta semua peserta untuk meninggalkan *chatroom*.

Selama diskusi, mohon berikan kesempatan bagi setiap orang untuk berbagi pandangannya, tetapi hanya satu orang yang menjawab pertanyaan dalam satu waktu. Langsung saja berikan pandangan Anda ketika Anda merasa ingin menyampaikan sesuatu, moderator tidak akan bertanya satu per satu ke setiap orang untuk setiap pertanyaan. Mohon diingat bahwa moderator ingin mendengar pandangan dari semua peserta. Tidak masalah untuk berbeda pendapat dengan peserta lainnya, namun mohon juga menghormati pendapat orang lain. Semua yang Anda dengar hari ini harus dirahasiakan dan tidak dibagikan atau diceritakan kepada siapapun diluar kelompok diskusi ini. Diakhir diskusi, semua percakapan dalam diskusi kita ini akan disimpan. Percakapan dalam diskusi kita ini akan tetap bersifat rahasia. Hanya tim peneliti yang akan membaca isi percakapan. Diskusi akan berlangsung sekitar satu jam.

Mohon jangan lupa untuk memberikan tanggal dan waktu ketersediaan Anda untuk DKT daring kepada peneliti/asisten peneliti saat pertemuan awalan. Anda akan mendapatkan pemberitahuan tentang jadwal DKT daring Anda dari peneliti/asisten peneliti yang diinformasikan melalui email/WhatsApp/Line. Dalam penelitian ini Anda akan membutuhkan perangkat elektronik untuk menghadiri diskusi daring seperti ponsel, komputer, tablet, laptop, dan sejenisnya. Mohon patuhi protokol kesehatan terlampir.

Jika Anda memiliki pertanyaan lebih lanjut mengenai penelitian ini, mohon jangan sungkan untuk menghubungi kami:

Ahmad Junaedi (Peneliti)

Tel/Whatsapp: +62-812-9026-8627

E-mail: [ajunaedi@m.u-tokyo.ac.jp](mailto:ajunaedi@m.u-tokyo.ac.jp)

Line: @junweasley

Fauzan Rachmatullah (Asisten Peneliti)

Tel/Whatsapp: +62-812-2169-9625

E-mail: [fauzan.rachmatullah23@gmail.com](mailto:fauzan.rachmatullah23@gmail.com)

Line: @fauzanmadkip

Untuk Peneliti dan Asisten Peneliti (Panduan wawancara untuk DKT daring)

Diskusi Kelompok Terumpun daring

Selamat pagi/siang/sore/malam.

Terima kasih atas waktunya untuk bergabung dengan Saya dan untuk berbicara mengenai pengalaman pembatasan fisik selama pandemik Covid-19. Perkenalkan nama saya **Ahmad Junaedi, mahasiswa S2 di school of International Health, The University of Tokyo Fauzan Rachmatullah, asisten peneliti dalam penelitian ini**. Tujuan dari penelitian ini adalah untuk **mengidentifikasi tantangan dan faktor pendukung dalam pembatasan fisik di kalangan dewasa muda di Jabodetabek**. Cara terbaik untuk mencapai tujuan penelitian tersebut adalah berbicara kepada orang yang mengalami sendiri pembatasan fisik di Jabodetabek, jadi saya menyelenggarakan DKT daring ini dengan beberapa orang dewasa muda pada bulan ini, termasuk dengan Anda pada saat sekarang ini. Dalam diskusi kita pada hari ini, Saya hanya ingin berbicara mengenai pengalaman Anda dalam pembatasan fisik, apakah kesulitan yang Anda alami dan faktor pendukung apa saja yang Anda miliki dalam penerapan pembatasan fisik.

Di dalam kelompok, Saya akan membahas lembar persetujuan penelitian (*informed consent*) sebelum kita memulai diskusi kita ini untuk meyakinkan bahwa Anda semua sudah paham mengapa kita saat ini berdiskusi dan juga untuk memastikan bahwa Anda berpartisipasi secara sukarela dalam diskusi ini. Saya menyediakan sebuah tautan situs yang berisi lembar informasi dan lembar persetujuan penelitian (*informed consent*). Jadi mohon untuk klik tautan yang saya bagikan di *chatroom* ini dan baca lembar informasi dan lembar persetujuan penelitian (*informed consent*). Setelah itu mohon untuk menandatangani lembar persetujuan penelitian (*informed consent*).

Saya tidak memiliki pengalaman dalam pembatasan fisik di Jabodetabek dan saya hanya mengumpulkan informasi mengenai hal itu, oleh karenanya Saya berharap Anda akan merasa nyaman untuk berbagi cerita kepada saya mengenai apa yang ada dalam pikiran Anda tentang pengalaman pembatasan fisik. Mohon untuk tidak merasa malu, Saya ingin mendengar dari Anda semuanya yang ada di sini mengenai pengalaman Anda selama pembatasan fisik. Anda adalah ahlinya karena Anda telah mengalami pembatasan fisik sekitar dua sampai tiga bulan dan saya di sini ingin belajar dari Anda semua. Tidak ada jawaban yang benar atau yang salah, saya hanya ingin mendengar apa yang ada dalam pemikiran dan saran dari Anda. Saya memiliki beberapa pertanyaan untuk Anda nanti tetapi seiringan dengan ini Anda juga bebas untuk menambahkan hal lain yang mungkin menurut Anda penting.

Diskusi ini akan direkam dan pada akhir diskusi saya akan menyimpan rekaman diskusi kita kali ini. Diskusi kita ini akan tetap bersifat rahasia. Hanya tim peneliti yang akan mendengarkan rekaman dan membaca transkripnya. Apakah ada yang keberatan jika diskusi ini direkam?

Selama diskusi, setiap orang dipersilakan untuk membagikan pemikiran kalian semua, tetapi hanya satu orang yang menjawab dalam satu waktu. Dipersilakan untuk langsung menjawab atau menambahkan jawaban atau opini jika ada sesuatu yang ingin dikatakan, Saya tidak akan bertanya ke setiap orang satu per satu untuk setiap pertanyaan. Mohon diingat bahwa saya ingin mendengar pemikiran Anda semua. Sangat tidak masalah jika tidak setuju dan berbeda pendapat dengan peserta lain tetapi mohon juga untuk tetap menghormati pemikiran orang lainnya. Semua yang Anda dengar hari ini harus dirahasiakan dan tidak dibagikan atau diceritakan kepada siapapun diluar kelompok diskusi ini. Diskusi akan berlangsung sekitar 1 jam. Apakah ada yang ingin ditanyakan sebelum kita mulai?

**Mari perkenalkan diri**

1. Mari sebutkan nama panggilan dan asal tempat tinggal. Bisa dimulai dari *menyebutkan identitas anonim salah satu peserta*

Lamanya pembatasan fisik

**Pertama-tama, Saya ingin tahu mengenai berapa lama Anda melakukan pembatasan fisik seperti tetap tinggal di rumah, menghindari kerumunan, dan menghindari untuk bertemu dengan orang lain secara langsung**

1. Berapa lama Anda telah melakukan pembatasan fisik?

(*probing*: menyebutkan setiap tindakan pembatasan fisik)

1. Apakah Anda mengalami masalah atau kesulitan (untuk melakukan pembatasan fisik)?

(*probing*: Mengapa?)

Kepatuhan, Tantangan, dan Faktor pendukung dalam tindakan pembatasan fisik

**Sekarang mari kita fokus dalam tindakan pembatasan fisik. Ada tiga tindakan utama dalam pembatasan fisik yaitu yang pertama menghindari penggunaan kendaraan umum/jam sibuk, yang kedua menghindari kontak fisik seperti bersalaman, berpelukan, jaga jarak satu meter, dan yang ketiga yaitu menghindari untuk bertemu dengan siapapun seperti tetap di rumah saja, kerja atau belajar dari rumah, menghindari kerumunan, dan menghindari untuk bertemu dengan teman atau keluarga yang tidak tinggal dalam satu rumah.**

1. Apakah Anda mematuhi tiga tindakan utama pembatasan fisik tersebut?
2. Tindakan pembatasan fisik yang mana yang menurut Anda sulit untuk dipatuhi?
3. Apa yang membuat Anda berpikir bahwa tindakan pembatasan fisik tersebut, yang ditanyakan pada pertanyaan sebelumnya (pertanyaan 6), sulit untuk dilakukan?
4. Tindakan pembatasan fisik yang mana yang menurut Anda mudah untuk dipatuhi?
5. Apa yang membuat Anda berpikir bahwa tindakan pembatasan fisik tersebut, yang ditanyakan pada pertanyaan sebelumnya (pertanyaan 6), mudah untuk dilakukan?

Tradisi aktivitas keagamaan

**Seperti yang kita ketahui selama pandemik Covid-19 di Indonesia, kita semua harus menyesuaikan dalam perayaan tradisi aktivitas Ramadan di Indonesia termasuk Mudik, Buka Bersama, Ngabuburit, Tarawih, dan Halal bi halal. Meskipun diantara kalian semua ada yang bukan Muslim, tetapi kadang-kadang Anda juga ikut bergabung dalam aktivitas tersebut dengan teman Muslim Anda atau di acara yang diadakan oleh kantor atau sekolah Anda.**

1. Menurut Anda, apakah menghindari aktivitas tersebut (Mudik, Buka Bersama, Ngabuburit, Tarawih, dan Halal bi halal) sulit atau mudah?
2. Apa yang membuat Anda berpikir bahwa menghindari aktivitas tersebut (Mudik, Buka Bersama, Ngabuburit, Tarawih, dan Halal bi halal) sulit atau mudah?
3. Seperti yang baru-baru ini diberitakan bahwa ada kluster baru penularan Covid-19 ini justru dari tempat bekerja, Apa ada tanggapan atau harapan yang ingin disampaikan ke pemerintah/tempat bekerja/kuliah untuk mendukung teman-teman?

**Apakah ada hal-hal lain tentang tantangan dan faktor pendukung dalam pembatasan fisik yang Anda ingin bagikan ceritanya sebelum kita sudahi diskusinya?**

Inilah akhir dari DKT daring. Terima kasih banyak telah bergabung dan berbagi pengalaman dan opini Anda dengan Saya. Saya ingin meminta Anda untuk klik tautan ini untuk memilih salah satu *voucher* senilai Rp100.000 sebagai insentif telah terlibat dalam penelitian ini.
