# Supplementary material for: Factors influencing physical distancing compliance among young adults during COVID-19 pandemic in Indonesia: A photovoice mixed methods study
Source: PLOS Glob Public Health. 2022 Jan 13;2(1):e0000035. doi: 10.1371/journal.pgph.0000035 (PMC10021510; doi:10.1371/journal.pgph.0000035)
Supplement: Attachment — Submitted filename: Response to Reviewers.docx [file pgph.0000035.s011.docx]

Responses to Reviewers

Reviewer 1

|  | Reviewer’s comment | Author’s response |
| --- | --- | --- |
| Abstract | | |
| 1 | Please consider clarifying the first sentence of the abstract, similarly to the beginning of the Introduction section. When was the spreading of the coronavirus disease out of control? | Thank you very much for your reviews and comments. We have addressed your comments below:  In the abstract, we revised accordingly in lines 19-20:  *The spreading of the coronavirus disease (COVID-19) is growing out of control in Indonesia since the first two confirmed cases were announced in March 2020.*  Also, in the introduction, we have revised the text accordingly in lines 65-67:  *The spreading of the COVID-19 infection is not under control in Indonesia. As of September 1, 2021, COVID-19 infection has been reported in every province with a total of more than 4.1 million cases and 133,000 deaths nationwide.* |
| Introduction | | |
| 2 | “These studies showed that several factors were associated with quarantine adherence such as social and psychological factors, knowledge of the disease, value…”  What kind of value? Please clarify the sentence. | We have clarified the sentence as you suggested in line 58:  *knowledge of the disease, cultural value, characteristics of the quarantine, and quarantine support* |
| Methods | | |
| 3 | Why did the authors decide to focus on the population aged 20-35 years? Other age groups were also heavily affected by the pandemic, and the workforce includes other age groups as well. Please consider explaining this choice in the text. Also, this could lead to issues with representativeness since the driving force behind the behavior – in this case, physical distancing compliance – of young adults might differ from that of, e.g., older adults. Please consider discussing this among the other limitations | This study employed an online survey with photovoice and focus group discussions. These methods require the ability to use the internet and devices such as smartphone, tablet PC, computer, or camera. We assumed that young adults are more familiar with them than older adults. In addition, the composition of young adult workers is about 38.6% in 2020 in Jakarta and the risk of infection is also high among them because the proportion of total COVID-19 cases for this age group was 24.8% since 2020 until now. So, young adults have a high risk to be infected during their daily commuting from home to workplace. Based on these reasons, we targeted the population aged 20-35 in our study.  We addressed this in the manuscript lines 65-68:  *As of September 1, 2021, COVID-19 infection has been reported in every province with more than 4.1 million cases and 133,000 deaths nationwide [13]. Among those infected, 24.8% of confirmed cases were among the 19–30 age group [13].*  And lines 75-77:  *The risk of local transmission is high in Jakarta. Jakarta is the second-largest metropolitan area in Asia after the Tokyo Metropolitan Area, and 38.6% of the workforce are young adults who commute daily in this city [16,17].*  The sampling method could affect the representativeness. We have clarified this limitation in lines 418-419:  *First, online recruitment through social media could only target active users which might have selection bias.* |
| 4 | “The level of physical distancing was evaluated as a total score from nine physical distancing measures where a higher score indicates higher compliance.”  What were the potential answers to the physical distancing measure questions? How were the answers scored? What is the total score? The total score is first mentioned in the results section, while Figure 1 shows the possible answers to the questions. However, this should be explained as part of the methodology. | The potential answers to each component of physical distancing measures were “never”, “sometimes”, and “always” as shown in Figure 1. Then, we scored the answer into 1 for never, 2 for sometimes, and 3 for always.  The total score is 27 (with 9 as the minimum score) as we have explained in line 199:  *The mean compliance score was 23.2 points out of 27.0 points.*  In lines 109-114, we have explained the total score, and we have added how we scored as you suggested:  *The level of physical distancing was evaluated as a total score from nine physical distancing measures. These nine measures were 1) maintaining a 1-meter distance, 2) avoiding handshakes, 3) avoiding hugs, 4) avoiding public transportation, 5) working/studying from home, 6) avoiding gatherings and crowds, 7) postponing meetings, 8) avoiding visiting elderly people, and 9) praying at home. For each measure, “never” was scored as 1, “sometimes” was scored as 2, and “always” was scored as 3.* |
| 5 | “The exposure variables in this study were sociodemographic characteristics; COVID-19-related variables such as COVID-19 testing, knowledge of COVID-19, etc.; and religious and tradition-related activities such as breaking fast during Ramadan, joining Mudik tradition, etc.”  “Model 1 was adjusted for sociodemographic characteristics. Model 2 was adjusted for both sociodemographic and COVID-19-related variables. Model 3 was additionally adjusted for religious and tradition-related variables.”  Please make a detailed list of the main exposure variables and covariates in the text. Consider creating a separate sub-section (or sub-sections) to provide this information. It should be clear which variables were included in each model, so categorizing the factors (e.g., sociodemographic characteristics, COVID-19-related variables, and religious and tradition-related variables) when listed might be reasonable as well. | For better readability in the manuscript, we decided to only mention general variables such as COVID-19 related variables and religious and tradition-related activities. We listed all the variables in the Methods section to explain how we conducted statistical analyses. We also changed the term of exposure to predictor and explained briefly how these variables were constructed. All changes can be found in lines 114-124:  *The predictor variables were COVID-19-related variables such as COVID-19 testing, knowledge of COVID-19, etc.; and religious and tradition-related activities such as breaking Ramadan fast together, joining mudik tradition, etc. Mudik is a traditional homecoming exodus in Indonesia that happens during Eid, the biggest Muslim holiday celebration. The question related to COVID-19 variables were adapted from the WHO European region COVID-19 survey tool and previous COVID-19 survey in the United States and the United Kingdom [21,22]. These variables were constructed based on composite questions and scored based on its answer. For example, for the knowledge of COVID-19 variables, each question under this variable was scored “1 for true answer” and “0 for false answer”. Covariate variables were sociodemographic characteristics. Details about the COVID-19-related variables and religious and tradition-related activities are shown in Table S1.*  For better readability in the main manuscript, we only listed all variables in the method (data analyses section) in lines 133-144:  *Hierarchical linear regression analyses were performed to identify facilitators and barriers to physical distancing compliance. In step 1, Model 1 was adjusted for sociodemographic characteristics such as age, place of working/studying, place of living, sex, education level, occupation, resuming work at office/study at school, living situation, and monthly income/allowance. In step 2, Model 2 was adjusted for both sociodemographic and COVID-19-related variables such as COVID-19 testing, knowledge of COVID-19, knowledge of COVID-19 prevention, perceived severity, perceived susceptibility, reasons to compliance, feasibility to compliance, duration of restriction compliance, understanding physical distancing guideline, perceived benefit, receiving workplace/school support, and receiving government support. In step 3, Model 3 was additionally adjusted for religious and tradition-related variables such as special prayer together outside during pandemic, breaking Ramadan fast together outside during pandemic, religious celebration during pandemic, and mudik tradition during pandemic.* |
| 6 | Please explain how the hierarchical models were defined. E.g., is this a two-level model where individuals are nested within living areas? | Analysis of nested data is used by hierarchical linear modeling while this study used hierarchical linear regression. Hierarchical linear regression deals with how predictor (independent) variables are selected and entered into the model. We have explained it in our response to your comment in point 5. |
| 7 | Please consider including a short explanation of the content of the topic guide for online FGDs, maybe as supplementary material. | Per your suggestion, we have added one sentence to explain the topic guide briefly in lines 157-161:  *The content of the topic guide includes: 1) how long the participants complied with physical distancing for, 2) what kind of physical distancing measures and religious tradition activities were difficult or easy to comply during the pandemic and the reason behind it, and 3) how their photos supported their explanation of their experience on physical distancing compliance.* |
| Results | | |
| 8 | “A graph of physical distancing compliance among young adults for each measure”  What type of measures are shown on the graph? Please clarify the Figure title. | We have added the type of measures in line 204:  *A graph of compliance among young adults for each physical distancing measure* |
| 9 | “As shown in Fig 1, the young adults had lower compliance for maintaining a 1-meter distance, working/studying from home, avoiding gatherings and crowds, and postponing meetings.”  Lower compliance than what? Also, what does it mean that it was lower? Please consider quantifying the presented results (e.g., x% of the participants never maintained a distance of 1-meter from others). | We have added the comparison of lower compliance. It means that young adults were less compliant to those measures than the rest of the physical distancing measures in lines 199-200:  *“As shown in Fig 1, compared with other physical distancing measures…,”* |
| 10 | Please consider describing the Table 2 Model 1 results using actual effect estimates in the text, similarly to the description of Model 3. | As our study used hierarchical regression, we think that our current explanation is enough. |
| 11 | Please consider including a short explanation or footnote on the Mudik tradition for the sake of international readers, especially since it is used as a separate risk factor from “religious celebration during pandemic”. It is first mentioned in the Discussion section in more detail, but an earlier explanation – maybe in the Methods section – might help readers understand the main results. | We have added a sentence about the Mudik tradition in lines 116-117 as it was first mentioned:  *Mudik is a traditional homecoming exodus in Indonesia that happens during Eid, the biggest Muslim holiday celebration.* |
| 12 | In Table 3, please consider reporting percentages as well – consistent with Table 1. | Based on your suggestion, we have added a “Total” column that includes actual numbers and percentages in Table 3. |
| 13 | Please consider using actual numbers and percentages to describe the results presented in Table 3 – similarly to Table 1 results. | Based on your suggestion, we have added actual numbers and percentages to describe the results presented in Table 3, lines 240-242:  *Most of them lived in the Jakarta area (n = 20, 42.5%), worked in Jakarta Selatan (n = 23, 48.9%), and had returned to work at an office and study at a school after the PSBB transition on June 5, 2020 (n = 30, 63.8%) (Table 3).* |
| 14 | Grammatical errors in the quoted texts of the qualitative findings are intended to reflect the style of the original Indonesian voice recording (e.g., “did not affect to my work”)? If not, please carefully correct the translated text. | Yes, all the quoted texts are intended to reflect the style of the original Indonesian voice recording. |
| 15 | Figure 3: “Understanding the content of the physical distancing guideline was an essential driver in increasing the awareness to comply with physical distancing.”  In the results section, the following statement could be found: “From the qualitative result, young adults mentioned that some actions that led to non-compliance, such as using crowded elevator and joining social gathering, should be avoided, as written in the guidelines.” If the FGD participants mentioned the guidelines, a more fitting quote could be used for Figure 3 under the “qualitative results”. Otherwise, self-awareness and fear might not necessarily be related to the guidelines. | There was no FGD participants that mentioned the guideline directly. Since the integration and joint display are the combination between the quantitative results and the qualitative results, we inferred our finding based on this combination. In the quantitative results, understanding the guideline was significantly associated with compliance. Whereas in the qualitative results, no one literally mentioned the guideline, but their actions showed their understanding and following the guideline (avoiding the crowded elevator, following the sign inside the elevator). This could result from self-awareness and fear as a reason to comply. We have revised the explanation as suggested in Figure 3:  *Figure 3: “Understanding the guideline was an essential driver in increasing the awareness to comply with physical distancing.”* |
| Discussion | | |
| 16 | “Second, its barriers were celebrating the religious day, having hometown in the Jakarta Metropolitan Area, resuming work…”  I believe here the authors meant “resuming work at the office”. Please clarify the sentence. | We have clarified it as you suggested in lines 340-342:  *Second, its barriers were celebrating the religious day, having hometown in the Jakarta Metropolitan Area, resuming work at the office, and social pressure to celebrate religious days with family.* |
| 17 | “Moreover, this study found that becoming an entrepreneur was an alternative after losing a job due to the pandemic. Consequently, hindering physical distancing compliance due to the need to start a business.”  This is first mentioned in the Discussion section. If this is an important finding of the qualitative part of the study, please consider explaining it first in the Results section with the relevant quotes. | We have added explanation as you suggested in lines 279-281:  *Young adults also explained that having support from their workplace encouraged their compliance. However, a young adult who work in informal sector had difficulties to comply with physical distancing measures. Workplace policies and supports can be both facilitator and barrier.*  And added relevant quote in lines 289-292:  *“I just quit the company in February 2020, so for the workplace policy I didn't know very well, to be honest I just started a business... ...because I just started my business so I need to meet people, so I had difficulties to maintain distance, uh.. to avoid to meet with others…” – Man, 23 years old, entrepreneur, Kota Tangerang Selatan, Online FGD.* |
| 18 | Similarly, a difference in physical distancing compliance between those who work in the formal vs. the informal sector was first introduced in the Discussion section. Please consider explaining this as well in the Results section with the relevant quotes. | We have responded to your suggestion as explained in point 17 |
| 19 | Snowball sampling has several limitations, from sampling bias to issues with representativeness. Please consider discussing this among the limitations. | We have addressed this important point in lines 418-419:  *First, online recruitment through social media could only target active users which might have selection bias.* |

Reviewer 2

|  | Reviewer’s comment | Author’s response |
| --- | --- | --- |
| Introduction | | |
| 1 | Line 76: “Moreover, various cultural and religious activities take place from time to time” – authors might want to clarify/justify the relevance of these activities to the scope of the study. | Thank you very much for your reviews and comments. We have addressed your comments below.  Indonesia recognizes at least 633 ethnic groups and 6 official religions across the archipelago. It has routine activities, for example, Muslims pray five times a day at a mosque and other religions pray once a week at prayer houses. Based on these situations, we justified that Indonesia may have unique barriers and facilitators to physical distancing compliance.  We have revised per your suggestions in lines 77-79:  *Moreover, as Indonesia has 633 ethnic groups and 6 official religions across the archipelago, various cultural and religious activities take place from time to time* *that can make it difficult for people to keep physical distance with others [18.19].* |
| Methods | | |
| 2 | I suggest including all “exposure variables” and stating clearly how they were scored | For better readability in the main text, we decided to only mention general variables such as COVID-19 related variables and religious and tradition-related activities. We listed all the variables in the Methods section when explaining how we did statistical analyses. We also changed the term of exposure to predictor and explained briefly how these variables were constructed. All changes are in lines 114-124:  *The predictor variables in this study were COVID-19-related variables such as COVID-19 testing, knowledge of COVID-19, etc.; and religious and tradition-related activities such as breaking Ramadan fast together, joining mudik^1^ tradition, etc. Mudik is a traditional homecoming exodus in Indonesia that happens during Eid, the biggest Muslim holiday celebration. The question related to COVID-19 variables were adapted from the WHO European region COVID-19 survey tool and previous COVID-19 survey in the United States and the United Kingdom [21,22]. These variables were constructed based on composite questions and scored based on its answer. For example, for the knowledge of COVID-19 variables, each question under this variable was scored “1 for true answer” and “0 for false answer”. Covariate variables were sociodemographic characteristics. Details about the COVID-19-related variables and religious and tradition-related activities are shown in Table S1.* |
| 3 | I suggest including the scoring of the main outcome measure, physical distancing | In lines 109-114, we have explained the scoring of the main outcome measure as you suggested:  *The level of physical distancing was evaluated as a total score from nine physical distancing measures. These nine measures were 1) maintaining a 1-meter distance, 2) avoiding handshakes, 3) avoiding hugs, 4) avoiding public transportation, 5) working/studying from home, 6) avoiding gatherings and crowds, 7) postponing meetings, 8) avoiding visiting elderly people, and 9) praying at home. For each measure, “never” was scored as 1, “sometimes” was scored as 2, and “always” was scored as 3.* |
| 4 | I think it is very important that the authors differentiate between predictor, covariate (currently called exposure variables, I believe incorrectly) and outcome measures- and at a minimum state what construct each variable is measuring and how it was scored and used in analyses. | We have addressed your concern based on your comment point 2.  The main outcome was revised based on your comment point 3. |
| 5 | For the quantitative data analysis – I recommend authors state clearly which variables were included in each model, in what order. It is unclear from the method (and also from the results) how many blocks/steps were included in each regression model.  Eg, In model one, we enter variables XX, YY, and ZZ in step 1, AA, BB, CC in step 2, and (if relevant) AA and BB in step 3. | For better readability, we decided to keep our explanation concise. There were three steps in hierarchical linear regression analyses: first step, we included all sociodemographic characteristics and main outcome. Then, we included all COVID-19 related variables in the second step. Finally, the third step, we included all religious and tradition-related variables. In the final model, all variables were included because we believed that these variables were important to physical distancing compliance. We have clarified in lines 133-144:  *Hierarchical linear regression analyses were performed to identify facilitators and barriers to physical distancing compliance. In step 1, Model 1 was adjusted for sociodemographic characteristics such as age, place of working/studying, place of living, sex, education level, occupation, resuming work at office/study at school, living situation, and monthly income/allowance. In step 2, Model 2 was adjusted for both sociodemographic and COVID-19-related variables such as COVID-19 testing, knowledge of COVID-19, knowledge of COVID-19 prevention, perceived severity, perceived susceptibility, reasons for compliance, feasibility for compliance, duration of restriction compliance, understanding physical distancing guideline, perceived benefit, receiving workplace/school support, and receiving government support. In step 3, Model 3 was additionally adjusted for religious and tradition-related variables such as special prayer together outside during pandemic, breaking Ramadan fast together outside during pandemic, religious celebration during pandemic, and mudik tradition during pandemic.* |
| Results | | |
| 6 | Paragraph above Table 2 (lines 177-183) – it appears that one of predictors included in Model 2, i.e., working in the health sector, was never mentioned in the method. Another predictor for the same model, never working/studying from home, it looks like this item is one of the 9 items/domains included in the outcome measure. Using the same items as both predictor and outcome is not an appropriate approach. Authors should clarify in the method which measures were used as predictors and which were used as outcomes (see comment 5 above). | Working in the health sector is under occupation variable (covariate: sociodemographic characteristics). We have addressed your point and mentioned it in the Methods section as we responded to your comment point 5.  Never working/studying from home is not the same as one of the outcome items. Never working/studying from home is under covariate: sociodemographic characteristics: resuming work at office/study at school.  It is different from work/study from home as one of the outcome items. They were obtained from two different questions. |
| Quantitative Results | | |
| 7 | In several parts of the results (quantitative), there is information pertaining to the method which should be deleted from the results and added to the method, data analysis section.  Eg., line 193: “In model 3, after adjusting for religious and tradition-related variables, in addition to the variables in models 1 and 2..”…this part should be clearly described in the method, in data analysis for Model 3, in the results, authors should simply report the results of the regression. | We have mentioned the information in the Methods section as you suggested and have addressed your comment point 5.  In the Results section, we modified the sentences just to report the results. |
| 8 | Table 2 – authors should report all variables included in the regression with the respective Beta coefficients and some other measure of significance as opposed to reporting solely the statistically significant predictors. This is a serious error in reporting and should be necessarily fixed!!! | We appreciate this very important comment.  We have reported all variables included in the regression on S2 Table (Supplementary), but for better readability in the main manuscript we decided to show only Table 2 because Table S2 is too large to put in the manuscript. |
| 9 | Some of the measures in Table 2 were never described in the method. Authors should describe all measures/constructs used in analyses in the method section. | For better readability in the main text, we did not describe it, but we revised it as you suggested in comment point 4:  *These variables were constructed based on composite questions and scored based on its answer. For example, for the knowledge of COVID-19 variables, each question under this variable was scored “1 for true answer” and “0 for false answer”. Covariate variables were sociodemographic characteristics. Details about the COVID-19 related variables and religious and tradition-related activities are shown in Table S1.* |
| Qualitative Results | | |
| 10 | Line 209 authors mention data saturation. It would be good to include the criteria to establish data saturation in the method, qualitative data analysis section. | As you suggested, we have added two sentences to describe how data saturation is reached, in lines 169-171:  *Data saturation is reached when no new information was obtained from additional FGDs. This was confirmed by referring to the fieldnotes and the themes in the codebook.* |
| 11 | Qualitative findings – In general, I recommend including more detail and contextualization of each theme to make it more meaningful for the reader. Overall for all themes, it would be useful to phrase the name of the theme to reflect a barrier or a facilitator (or both, if relevant to their data) to physical distancing. The first theme appears to be a barrier whereas the second appears to be a facilitator. | Based on your suggestion, we have arranged the order of the themes to be more meaningful for the readers; we mentioned barriers, then facilitators, and finally themes that can be both barrier and facilitator.  Lines 247-250:  *Implementation of the government policy*  *The first theme described a barrier to comply with physical distancing measures. Young adults expressed their views on the implementation of the government policy. The implementation hindered their compliance with physical distancing measures.*  Lines 254-256:  *Having social pressure*  *The second theme also described a barrier. Young adults also expressed their uneasiness about complying with physical distancing because it could go against social norms.*  Lines 265-270:  *Having clear and easy-to-understand indicators of physical distancing at public facilities*  *The third theme described a facilitator to compliance. One of the young adults mentioned the ease of maintaining distance during Friday prayer in a mosque. Another young adult also shared her experience at a coffee shop that had clear indicators to help her to comply with physical distancing measures.*  Lines 278-281:  *Workplace policies and supports*  *Young adults also explained that having support from their workplace encouraged their compliance. However, a young adult who work in informal sector had difficulties to comply with physical distancing measures. Workplace policies and supports can be both facilitator and barrier.*  Additional relevant quote in Lines 289-292:  *“I just quit the company in February 2020, so for the workplace policy I didn't know very well, to be honest I just started a business... ...because I just started my business so I need to meet people, so I had difficulties to maintain distance, uh.. to avoid to meet with others…” – Man, 23 years old, entrepreneur, Kota Tangerang Selatan, Online FGD.* |
| 12 | While the citations are great to include to support the claims made about the topic, I recommend authors conduct a more in-depth analysis and at a minimum try to summarize what the participants are saying and then support their argument/claims with the qualitative data. | We have conducted an in-depth analysis of the qualitative data. We have also summarized what the participants said as you recommended in the Discussion section in lines 356-359: *Having awareness fostered physical distancing compliance among young adults—being aware of what is going on around them increased their physical distancing compliance [29]. In this study, young adults were mindful of keeping themselves away from crowds, including avoiding unimportant gatherings.*  Also, in lines 361-364:  *Poor public awareness was one of the barriers to physical distancing, especially when using mass transportation. Even though young adults generally adhere to the physical distancing measures, however they cannot influence other people’s behavior.*  Also, in lines 394-396:  *The Work-at-Office policy introduced after the government lifted the restriction, acted as a barrier to physical distancing compliance. Young adults found it difficult to comply with physical distancing when they had to return to work at the office.* |
